# A fully integrated smart ring for daily biochemical monitoring

**DOI:** 10.1038/s41467-026-75980-z

**Published:** 2026-07-23

**Authors:** Tamoghna Saha, Shichao Ding, Siyu Qin, Hannah Fishman, Rosie Pham, Te Gu, Muyang Lin, Yushan Xian, Barak Sabbagh, Abdulhameed Abdal, Gaoyuan Ji, Kittiya Sakdaphetsiri, Yi Fung Winsten Lam, Muhammad Inam Khan, Hao Luan, Zongnan Wang, Selene Tang, Omeed Djassemi, Samar Singh Sandhu, Sheng Xu, Joseph Wang

**Affiliations:** 1 Aiiso Yufeng Li Family Department of Chemical and Nanoengineering, University of California San Diego, La Jolla, CA USA; 2 Department of Electrical and Computer Engineering, University of California San Diego, La Jolla, CA USA; 3 Department of Bioengineering, University of California San Diego, La Jolla, CA USA; 4 Department of Mechanical Engineering, University of California San Diego, La Jolla, CA USA

**Keywords:** Biomedical engineering, Diagnostic markers, Bioanalytical chemistry, Type 1 diabetes

## Abstract

Biochemical analysis complements biophysical measurements by capturing the underlying molecular and metabolic changes that drive physiological health. Here, we introduce a compact, fully integrated smart ring capable of simultaneously monitoring multiple chemical biomarkers in real-time — marking a true biochemical counterpart to existing smart rings. Our ring can continuously measure glucose, ketone, ascorbic acid, uric acid, lactate, and alcohol from passive osmotically extracted sweat, is powered by custom-built high-capacity flexible Zn-AgO batteries, and comprises ultra-compact, energy-efficient electronics for data processing and wireless transmission. It can support ~12 hours of continuous monitoring and preserves personalized calibration stability for ~2 months. The simultaneous biomarker monitoring was validated under diverse daily settings. Reliable, continuous monitoring of glucose and ketone levels showed strong agreement with commercial blood meters and continuous glucose monitors in both healthy and type 1 diabetic individuals, confirming the ring’s accuracy even for at-risk population. Overall, our ring extends beyond commercial smart rings focusing on only biophysical signals, by enabling continuous assessment of biochemical changes and advancing the development of next generation personalized and miniaturized healthcare technologies.

## Abstract (web summary)

Commercial smart rings lack biochemical sensing. Here, the authors present a fully integrated ring that measures multiple chemical biomarkers from osmotically extracted sweat without iontophoresis, validated for daily analysis in both healthy and type 1 diabetic individuals.

## Introduction

Wearable rings have recently gained significant traction, with rising sales driven by their compact design, comfortable wearability, and customizable features that seamlessly integrate into one’s daily life. Companies, such as Oura, Samsung, Ultrahuman, and Circular, are building smart rings that can simultaneously monitor several biophysical signatures, such as skin temperature, sleep patterns, heart rate variability, calories, and blood oxygen levels^1,2^. These smart rings are primarily marketed as wellness and fitness trackers, which can flag potential health anomalies. However, these rings provide only physiological information and lack the features needed to capture the body’s underlying biomolecular dynamics. Few attempts have been made to measure chemical biomarkers in sweat from the ring site (Table S1)^3,4^. However, these platforms are not “*true rings*” as they rely on only wrapping the device around the ring area, comprise bulky parts, and use electrical stimulation to access sweat for periodic measurements. Thus, unlike commercial smart rings, a “*true biochemical integrated counterpart*” that can readily access biofluids without any exertion or stimulation, support long-term dynamic molecular monitoring at the ring location, and seamlessly integrate into the users’ daily routines, remains unaddressed.

**Fig. 1:**
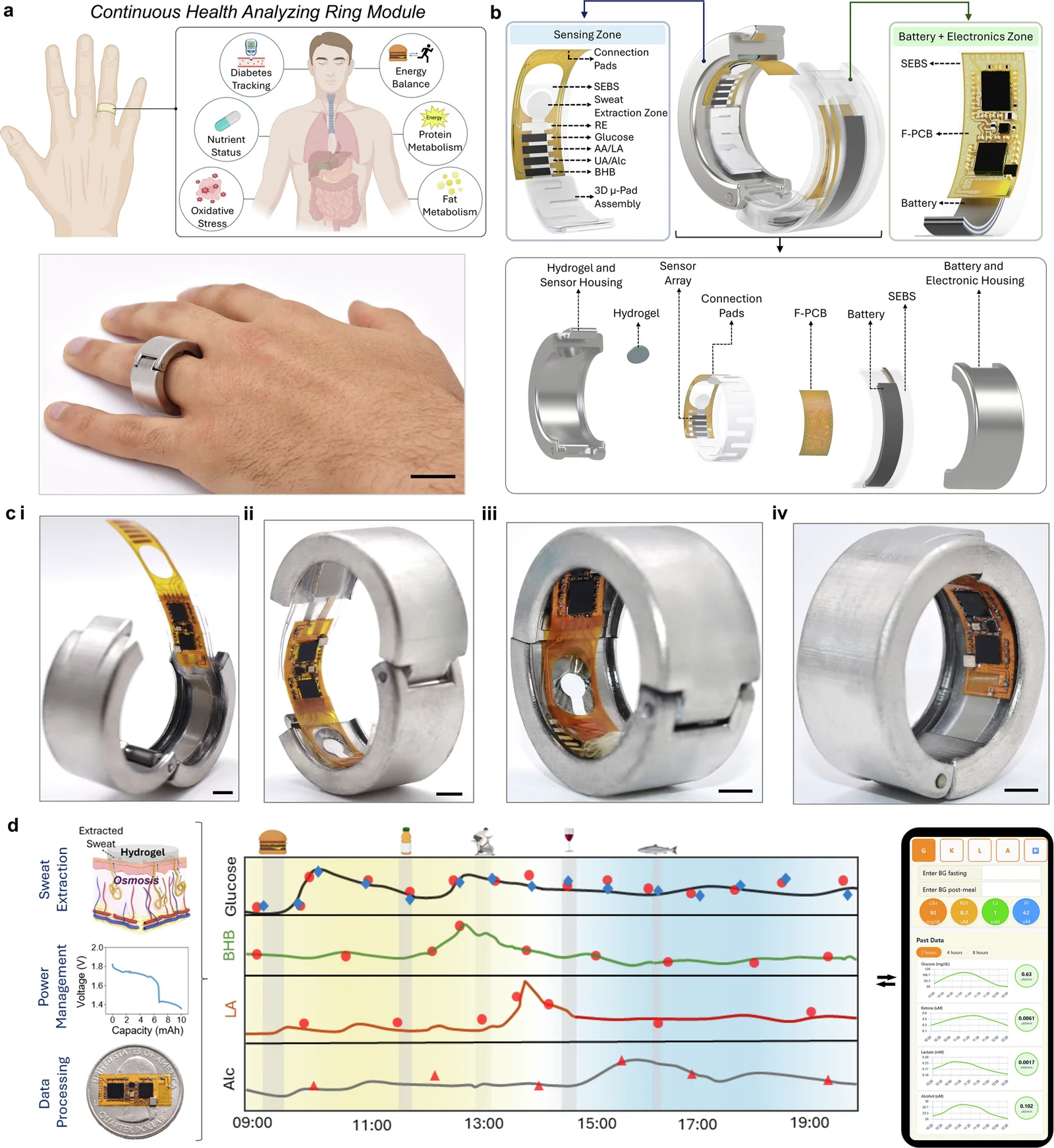
The overall vision of CHARM. **a** Schematic highlighting the types of health information that can be acquired from CHARM in daily life (top), and how it looks once worn (bottom). Scale bar: 1 cm. Schematic is created using BioRender (https://BioRender.com/ivti20s*)* and annotated in MS PowerPoint. **b** CHARM consist of two connected halves – Left: Comprising the sweat extraction, sensor array, and microfluidic assembly. Four electrochemical sensors are interfaced to the fluidic channel for real-time and simultaneous multiplexed biomarker monitoring from sweat. Right: Comprises the flexible battery and electronic assembly. Both halves are bridged by the connection pad, which connects to the sensor array from one end and the battery from the other. CHARM is designed to be worn on the finger. Scale bar: 1.5 cm. Schematic was prepared using Autodesk Fusion 360. **c** Optical images of CHARM’s i battery connecting the F-PCB with connection pads, and ii precise alignment of the connection pad with the hydrogel site opening. iii Image of the fully assembled device focusing the hydrogel slot; iv Image of the fully assembled device focusing the battery and electronics. Scale bar: 0.5 cm. **d** CHARM uses osmotically extracted sweat and flexible Zn–AgO batteries to power its miniaturized F-PCB, enabling continuous simultaneous sensing of multiple biomarkers and wireless data transmission and display to a smart device. Red dot: Blood biomarker concentration; Blue dots: Commercial CGM readings. Schematics were prepared using Autodesk Fusion 360 and MS PowerPoint.

To demonstrate the practical utility of a chemical biomarker ring analogue, we present here CHARM — A Continuous Health Analysing Ring Module. CHARM is a compact (5.1 gm, outer diameter = 3.0 cm), fully-integrated wearable ring platform that continuously monitors four biomarkers from sweat in real time and delivers actionable daily insights (Fig. 1a). Continuous biomarker monitoring is crucial because episodic measurements, such as food logs or occasional blood tests, miss rapid physiological changes, hydration shifts, and adherence lapses, limiting timely and personalized insights^5,6^. Amongst all categories, diabetes represents a key area that requires vigilant monitoring to enable informed daily decision-making, such as during hyperglycemia, hypoglycemia, or diabetic ketoacidosis (DKA). The onset of these phases is greatly affected by daily meals, lifestyle, exercise, and inter-personal factors^7,8^. Diabetic biomarkers are also linked to several critical health issues like obesity, cardiovascular diseases, gout, muscle and bone strength, neuropathy, kidney and pancreas functions, and fatigue^9,10^. Glucose has been the widely used biomarker for guiding diabetic related decisions through continuous glucose monitors (CGMs)^9,11^. Companies like Dexcom, Abbott, Medtronic, and Senseonics deliver insights by solely tracking glucose levels, either through epidermal (typically needle-based form factors penetrating skin) or implantable (surgically implanted under skin) form factors^12^. Despite the advantages of CGMs, glucose alone cannot serve as a universal biomarker for guiding daily lifestyle decisions and therapeutic interventions due to the complexity of the diabetes spectrum (type I, II, gestational, and prediabetes)^7,13^. Complex effects, such as time-in-range, postprandial spikes, dawn phenomena, and nocturnal hypoglycemia, cannot be fully explained by glucose alone^14^. Similarly, closed-loop insulin dosing systems require inputs from multiple biomarkers (beyond glucose) for determining accurate dosage amounts^15^. Currently, there are no commercial CGM models that can simultaneously monitor multiple diabetes biomarkers. While Abbott is pursuing continuous ketone monitoring due to its connection to DKA in type 1 diabetes (T1D) patients, no commercial products are yet released^16,17^. While commercial CGMs designed for interstitial fluid (ISF) sensing are widely available, sweat-based devices are limited, despite being relatively a more easy biofluid to access on skin, as they still face key challenges: (a) typically fail to operate continuously under fully passive (stimulus-free) conditions^18,19^; (b) often lack compact, wearable form factors that ensures user comfort and encourages consistent, at-home use; and (c) miss capturing long-term personalized signatures to enable actionable health decisions without blood calibration^20^. Thus, there is a clear need for wearable systems that measure multiple biomarkers beyond glucose, and miniaturised sweat-based platforms can offer an attractive user-friendly route for real-time daily multi-biomarker monitoring across all age groups.

CHARM is engineered to fit within a standard ring casing and be worn comfortably at the ring location (Fig. S1 and Supplementary Video 1). It can simultaneously monitor glucose, beta-hydroxybutyrate (BHB, ketone), ascorbic acid (AA), uric acid (UA), lactate (LA), and alcohol (Alc) (maximum four at a time to prevent excessive voltage drop between electrodes), from passively harvested sweat, supporting both diabetes and nutritional-related tracking (Fig. 1b)^21^. Thus, CHARM enables assessment of how multiple biomarkers collectively influence the glucose-ketone index (GKI). All these biomarkers are chosen as: a) BHB is a key indicator for ketosis^17^; b) AA fluctuates with diet and influences oxidative stress response during diabetes^22^; c) UA fluctuations are related to insulin resistance, gout, and cardiovascular risk^23^; d) lactate fluctuations are linked to anaerobic metabolism and exercise routine^24,25^; and e) alcohol consumption can lower blood glucose, mask hypoglycemic symptoms, and even increase chances for DKA^26,27^. CHARM comprises two semicircular 3D printed half rings, joined by a hinge on one end, allowing it to open and close from the opposite end for easy wearability. It features a dual zone design: a) a left compartment dedicated for sweat extraction and multiplexed biochemical sensor integration, and b) a right compartment housing the flexible arc-shaped batteries and electronic circuitry. The left zone integrates a hydrogel, a 3D stacked paper microfluidic channel assembly, and an electrochemical sensor array. The hydrogel is housed within a compact circular slot to enable controlled swelling and drying, while the circular portion of the channel remains sandwiched between the bottom hydrogel surface and skin. The right zone hosts an in-house developed printed and flexible Zn-AgO battery pack, which is connected to a highly miniaturized F-PCB for electronic readout and wireless data transmission (Fig. 1ci, ii). The F-PCB connects to the sensor array using the connection pads. The Zn-AgO battery pack is designed to meet the power demands of CHARM within its limited space while maintaining excellent mechanical properties. Leveraging the intrinsically safe aqueous Zn-AgO chemistry and high theoretical cathode capacity (423 mAh g⁻¹), the battery can deliver stable and efficient power to the integrated F-PCB. Figure 1c iii, iv shows the miniaturized electronics connecting the sensors and battery. Overall, the circular part of the paper channel, F-PCB, and connector pads interface the skin. Unlike conventional sweat patches that operate on physical activity, chemical stimulation or with high current densities (iontophoresis) to induce sweating, CHARM utilizes a hydrogel-initiated osmotic pressure difference to passively draw sweat from underneath the skin over extended periods (Fig. 1d)^28^. In this work, we also evaluate the viability of the unexplored finger-ring site for sweat measurements. The extracted sweat is directed through the stacked channel assembly, containing the electrochemical sensor arrays, enabling simultaneous and real-time tracking of four biomarkers at a time. The flexible battery can synergistically power the F-PCB for >12 hours, enabling continuous operation of four sensors. The embedded ultra-compact F-PCB contains the multichannel potentiostat (1.3 × 0.67 cm, ~90 mg) that combines low-power electrochemical sensing and wireless data transmission into a fully self-contained circuit. Its design offers high sensitivity and stability while significantly minimizing size and energy use, fitting seamlessly on the right half of the ring to detect multiple biomarkers and wirelessly relay their real-time profiles to a smartphone.

## Results

### Sensor and fluidic assembly performance with osmotic sweat sampling

**Fig. 2:**
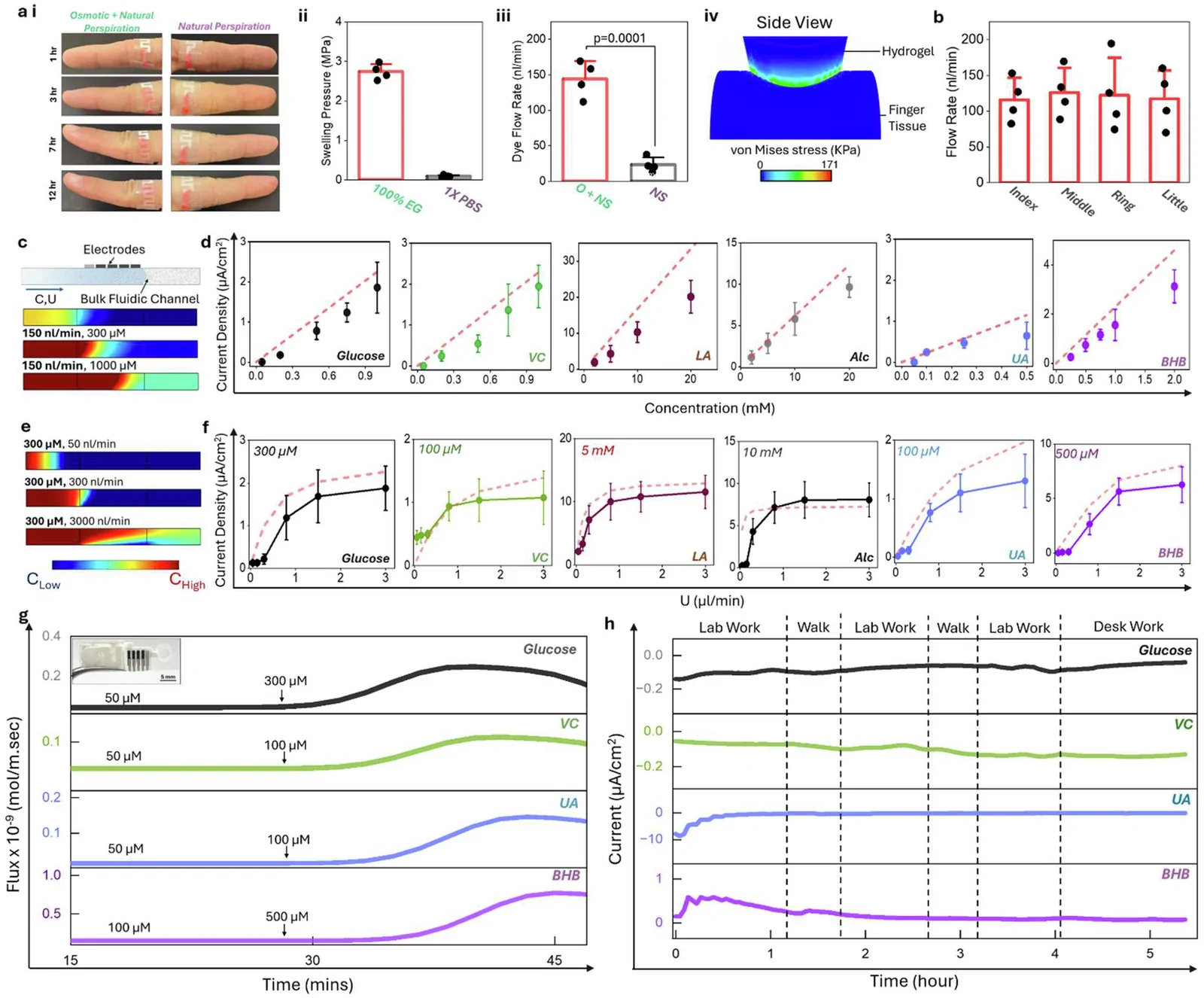
Osmotic sweat extraction and sensor performance with fluidics. **a** i) Optical image demonstrating enhanced dye transport in the fluidic channel from osmosis compared to natural perspiration alone at the ring site. ii) The resulting enhanced flow rate arises from higher (by ~*50X*) swelling pressure of the osmotic hydrogel vs. the non-osmotic gel. Bar denotes mean ± standard deviation (SD) from *n* = *4* data points (black dot). iii) Plot demonstrating that osmotic-driven sweat extraction yields 5-6 times higher flow rate vs. natural perspiration alone. Bar denotes mean ± SD from *n* = *4* data points (black dot). Significance was tested using one-sided unpaired two-sample t-test. iv) The von Mises stress profile shows that hydrogel swelling pressure far exceeds (by ~*35X*) finger-induced compressive stress, confirming extracted sweat as the channel fluid. **b** The osmotic flow rate also remains uniform across the ring site of all fingers throughout 10 h of testing period. Bar denotes mean ± denotes SD from *n* = *4* data points (black dots). **c** Current density contour profiles from FEA simulations performed at a constant flow rate with varying inlet concentrations. The schematic represents sensor-fluidic arrangement. **d** Plots comparing experimental average value (dot) and simulated (dashed) current density profiles under an inlet flow rate of 150 nL/min across varying concentrations of glucose, AA, lactate, alcohol, UA, and BHB. Error bar denotes SD from *n* = *3* measurements. **e** Current density contour profiles from FEA simulations performed at a constant concentration with varying inlet flow rates. **f** Plots showing a comparison between experimental and simulated current density profiles at different flow rates for 300 µM glucose, 100 µM AA, 5 mM lactate, 10 mM alcohol, 100 µM UA, and 500 µM BHB. Each dots denotes average value and error bars denote SD from *n* = *3* measurements. **g** Plots showing the estimated lag time from FEA simulations in reaching peak signals across multiple spikes of all four biomarkers running parallelly at 150 nl/min. Inset: Four sensor array on the fluidic channel. **h** Plots illustrating the long-term stability of simultaneous amperometric signals from four biomarkers during a stimulus-free on-body study, showing minimal effects of sweat-rate fluctuations during daily activities.

We first assessed the feasibility of osmotic sweat extraction and quantified the resulting sweat rate generated from the finger ring site (stained with red dye). For this, we used two patches comprising a PVA-PAAm hydrogel disk, where each was treated with pure ethylene glycol (EG) solution (for osmotic strength elevation) and 1X phosphate buffer solution (PBS, isotonic with sweat and mimicking natural perspiration extraction) (Fig. 2a i). The fluidic channel length was designed such that it took ~10 h for the liquid to reach the channel end at 150 nl/min (Fig. S2). The sampled sweat and dye moved forward through the channel via capillary action, which also prevented mixing of the old and new sweat. Pure EG-treated hydrogel possessed higher osmotic strength ( ~ 2800 kPa) vs. PBS hydrogel ( ~ 55 kPa) and subsequently led to greater dye (model biomarker) flow in the channel (Fig. 2a ii, S3, Supplementary Note 1)^5^. The total dye diffusion length achieved with the pure EG hydrogel ranged ~3-4 times greater than PBS hydrogel, which corresponded to a sweat rate of ~100–150 nL/min (Fig. 2a iii and Fig. S4). Von Mises stress analysis showed that pressures on the bottom hydrogel surface from the skin (bottom) and casing (top) ranged 85–90 kPa, confirming the channel fluid to be solely extracted sweat (Fig. 2a iv, Supplementary Note 2, Fig. S5, and Supplementary Video 2). The flow rate stayed constant across all fingers and ranged ~85% higher than natural perspiration and about 50–70% lower than the typical fingertip sweat rate, reflecting the lower sweat gland density at the ring site (Fig. 2b)^5^. Sweat rate variations are primarily driven by interpersonal differences rather than environmental factors, as osmosis is a colligative property. Such behaviors make the ring location attractive for sustained long-term sweat harvesting without compromising channel saturation, and an alternative, feasible location with greater compliance for daily monitoring. As a result, the net sampling time could prolong ~10 h, as seen from Fig. 2a i and Fig. S4. The sensor–fluidic assembly included a four-sensor array positioned in between the hydrogel slot and the onset of the 3D-stacked fluidic channel structure (Fig. 2c). All sensors were screen-printed on a flexible SEBS substrate, allowing them to withstand mechanical deformations. The in-vitro electroanalytical performance of all sensors is shown in Figs. S6–S11. To assess how sweat rate at the ring site influences the electrochemical response of all four sensors with the fluidic assembly, we conducted experimental tests and finite element analysis (FEA) simulations under two conditions: a) varying concentration at a constant flow rate, and b) varying flow rate at a constant concentration (Fig. 2c–f and Figs. S12–S14, Supplementary Note 3, and Table S2). At a flow rate of 150 nl/min, the sensor displayed a linear relationship with analyte concentration, aligning well with FEA simulation results (Fig. 2c, d). Based on the enzymatic activity, the estimated current density change over concentration ranged ~7, 10, 20, 25, 1, $3.2\frac{\mu A.{{cm}}^{-2}}{{mM}}$ for glucose, AA, UA, BHB, alcohol, and lactate, respectively. When the flow rate increased, the experimental response remained nearly constant up to 200–250 nl/min, after which it began to increase and eventually plateaued >1.5 µL/min, indicating a transition to a mass-transport-limited regime (Fig. 2e, f). These results indicate that operating under osmotic sweat rates effectively minimizes the impact of sweat rate on the sensor response. Since the sensors are positioned ~1.3 cm from the hydrogel inlet, we also performed FEA simulations to estimate the inter-sensor time lag required to reach peak flux at a specified input concentration profile (from low to high to low) (Fig. 2g). This time lag ranged ~2–3 min at 150 nL/min and was accounted for all our on-body trials. We also experimentally examined the potential influence of varying sweat rates during daily activities (like lab work, walking) and observed negligible effect on the sensor response (Fig. 2h). Overall, osmotic sweat extraction at the ring site demonstrates feasibility for long-term monitoring and holds great promise for providing effortless, exertion-free day-long extended sensing for the user.

### Wireless and flexible multichannel potentiostat

**Fig. 3:**
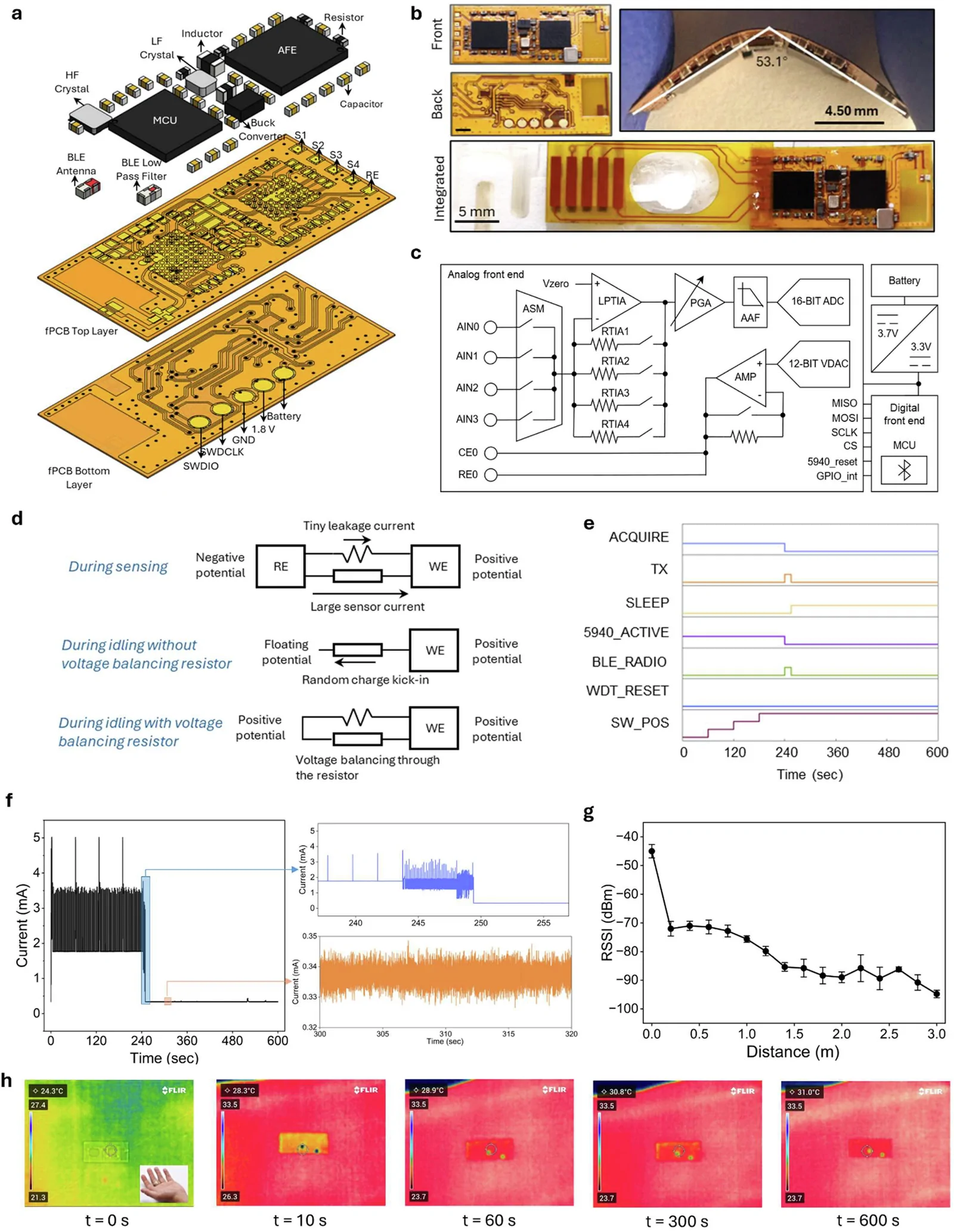
Wireless electronics architecture. **a** Schematic highlighting all circuit components and how they are arranged to build the miniaturized multichannel potentiostat. Schematic generated from shapr3D. **b** Optical images illustrating the front and back view of the electronic board and demonstrating its flexibility, with bending angles up to 53°. Scale bar: 2 mm. Image also showing how the F-PCB connects to the sensor array with the connection pads. **c** Block diagram of the functional circuit. **d** Strategies used to prevent signal drifts and unwanted reactions on the electrode surface during multichannel operation. Both (**c**) and (**d**) are designed in MS PowerPoint. **e** Digital timing diagram of the firmware control cycle. TX Transmission, WDT Watchdog timer, SW_POS Switching position. **f** Power consumption metrics of the potentiostat during active operation and sleeping. 1 complete sensing cycle: Working current: 2.36 mA; Sleeping current: 0.40 mA; Mean current: 0.83 mA. **g** The receiver signal strength (RSSI) of the potentiostat during multichannel operation, measured by a smartphone for up to 3 m. Each black dot point denotes average value and error bar denotes SD from *n* = 3 measurements. **h** Device temperature measurement under continuous operation when placed on the hand (inset).

The multi-channel CA sensing system integrates an AD5940 analog front end (AFE) for electrochemical sensing and a low-power NRF52840 digital front end (DFE) for system control, data handling, and wireless communication (Fig. 3a, Supplementary Note 4-8). The AFE generates the specified voltage waveforms for CA and performs current-to-voltage conversion through a high-gain transimpedance amplifier. The resulting voltage signal is digitized by a 16-bit ADC and temporarily stored in an onboard FIFO for later retrieval by the digital subsystem. A shared 1.8 V supply rail, generated by a miniature high-efficiency buck converter from a 3.6 V battery powers both front ends and enables the compact ring integration. The buck converter provides a stable 3.3 V supply, as it yields the lowest current noise (standard deviation ~$3.6{nA}$ vs. 4.1 and $5.7{nA}$ at 2.8 and 3.8 V) while maintaining accurate mean current, thereby improving chronoamperometric stability and reducing power consumption compared to direct battery operation (Table S3). All components are situated on a two-layer polyimide-based flexible PCB that can bend up to 53^0^ and is shorter than a US quarter coin (Fig. 3b, c, Fig. 1d). To support multi-channel sensing without replicating analog circuitry, the AFE employs a low-leakage analog switching matrix. All sensors share a common counter (CE)/reference (RE) electrode, while the working electrode (WE) is multiplexed to the AFE through the analog switch matrix. This arrangement enables a single set of core analog components—including the digital-to-analog converter (DAC), buffer amplifier, potentiostat interface, transimpedance amplifier (TIA), and analog-to-digital converter (ADC) to support all channels, while reducing size, complexity, and power consumption (Figs. S15–18). A firmware flow chart detailing how MCU handles BLE data transmission is shown in Fig. S19. Temporal multiplexing can leave inactive sensors with residual potential that can trigger unintended reactions. Thus, adding a 10 MΩ shunt resistor in parallel provides a DC relaxation path that drives the electrode toward 0 V without affecting sensitivity, preventing drift and cross-channel carryover while preserving measurement fidelity (Fig. 3d and Fig. S20).

The DFE is based on the nRF52840 microcontroller (MCU) selected for small form factor, low power consumption, and native BLE (Bluetooth low energy) support. It coordinates AFE sequencing, acquires digitized data via serial peripheral interface (SPI), and manages wireless transmission. A 32 MHz crystal ensures timing stability for high-performance operation and BLE, while a 32.768 kHz crystal supports low-power real-time clock operation during sleep. The RF path includes an integrated ceramic low-pass filter for harmonic suppression and out-of-band noise filtering, complemented by a compact ceramic antenna. The filter aids in removing out-of-band noise, and the final antenna tuning is refined through the RF front-end design and layout. The programming interface is provided through the underside serial-wire debug pads on the flexible PCB (Fig. 3a), which also hosts the main power interface, preserving accessibility while maintaining a clean top-side footprint for integration.

Power minimization is central for realizing long-term undisturbed continuous sensing. Since CA does not require high sampling rates, the ADC sampling frequency is restricted to 0.5 Hz. The DFE is programmed as a finite-state machine, with the “ACQUIRE” state meaning recording of the sensor readings, during which the analog switch matrix switches between all four channels to record and store the sensor data sequentially to a buffer (Fig. 3e)^29^. After completing all four channels, the DFE enters the “TX” state, and the BLE transceiver is enabled briefly to transmit the buffered data before being immediately disabled again to avoid unnecessary energy expenditure. This control scheme results in an average power consumption of 0.83 mA over a complete sensing cycle (Fig. 3f). Following transmission, the system enters a deep-sleep state for approximately eight minutes. Timing is maintained using the low-frequency crystal and watchdog timer, and the entire system autonomously wakes us to initiate the next sensing cycle. This duty-cycled architecture enables long-term, multi-channel electrochemical monitoring under strict power constraints. The restoration of automatic Bluetooth connection and wireless connection after sleep is shown in Fig. S21. Wireless data are streamed to a BLE-enabled host and visualized in real time using a custom MATLAB interface, which also allows wireless configuration of sensing parameters (channel, sampling rate, pulse duration, and applied potential). This removes the need for repeated MCU reprogramming when switching modes and enables flexible electrochemical sensing across different analytes. The optimal RSSI from the packaged on-body ring during multichannel operation is about 3 m (Fig. 3g). Additional RSSI measurements show that BLE range decreases from ~14 m (bare circuit) to ~5 m (packaged off-body) and ~3 m (on-body), indicating that packaging and body proximity, rather than intrinsic radio limits, govern the practical operating range of the wearable system (Fig. S22). The measured temperature increase remains well within ranges considered safe (27.3 °C–31.0 °C) after prolonged skin-contact, indicating that the F-PCB does not produce excessive thermal loading during normal operation (Fig. 3h). The multichannel sensor operation are shown in Figs. S23, 24.

### AgO-Zn flexible battery performance

The aqueous-phase stretchable AgO-Zn battery has been widely adopted in integrated flexible electronics owing to its inherent safe electrochemical performance and mechanical stability^30,31^. To meet the power demands of CHARM within its limited space, a thin and flexible AgO-Zn battery was designed as the power unit. This battery offers a high operating voltage and a superior theoretical cathode capacity (423 mAh g⁻¹), making it well-suited for integrated wearable applications. Here, the flexible AgO-Zn battery relies on the following anode reaction^32^:

1 $${\rm{Zn}}({\rm{s}}){+{\rm{2OH}}}^{-}({\rm{aq}}.)\leftrightarrow {{\rm{ZnO}}+{\rm{H}}}_{2}{{\rm{O}}+{\rm{2e}}}^{-}{,{\rm{E}}}^{{\rm{o}}}=-1{\rm{.22V}}\,{\rm{versus}}\,{\rm{SHE}}$$

and two step cathode reactions

2 $${\rm{2AgO}}({\rm{s}}){+{\rm{H}}}_{2}{{\rm{O}}+{\rm{2e}}}^{-}\leftrightarrow {{\rm{Ag}}}_{2}{\rm{O}}({\rm{s}}){+{\rm{H}}}_{2}{\rm{O}}({\rm{l}}){,{\rm{E}}}^{{\rm{o}}}=+ 0{\rm{.60V}}\,{\rm{versus}}\,{\rm{SHE}}$$

3 $${{\rm{Ag}}}_{2}{\rm{O}}({\rm{s}}){+{\rm{H}}}_{2}{\rm{O}}\leftrightarrow {\rm{2Ag}}({\rm{s}}){+{\rm{2OH}}}^{-}({\rm{aq}}.){,{\rm{E}}}^{{\rm{o}}}=+ 0{\rm{.34V}}\,{\rm{versus}}\,{\rm{SHE}}$$

**Fig. 4:**
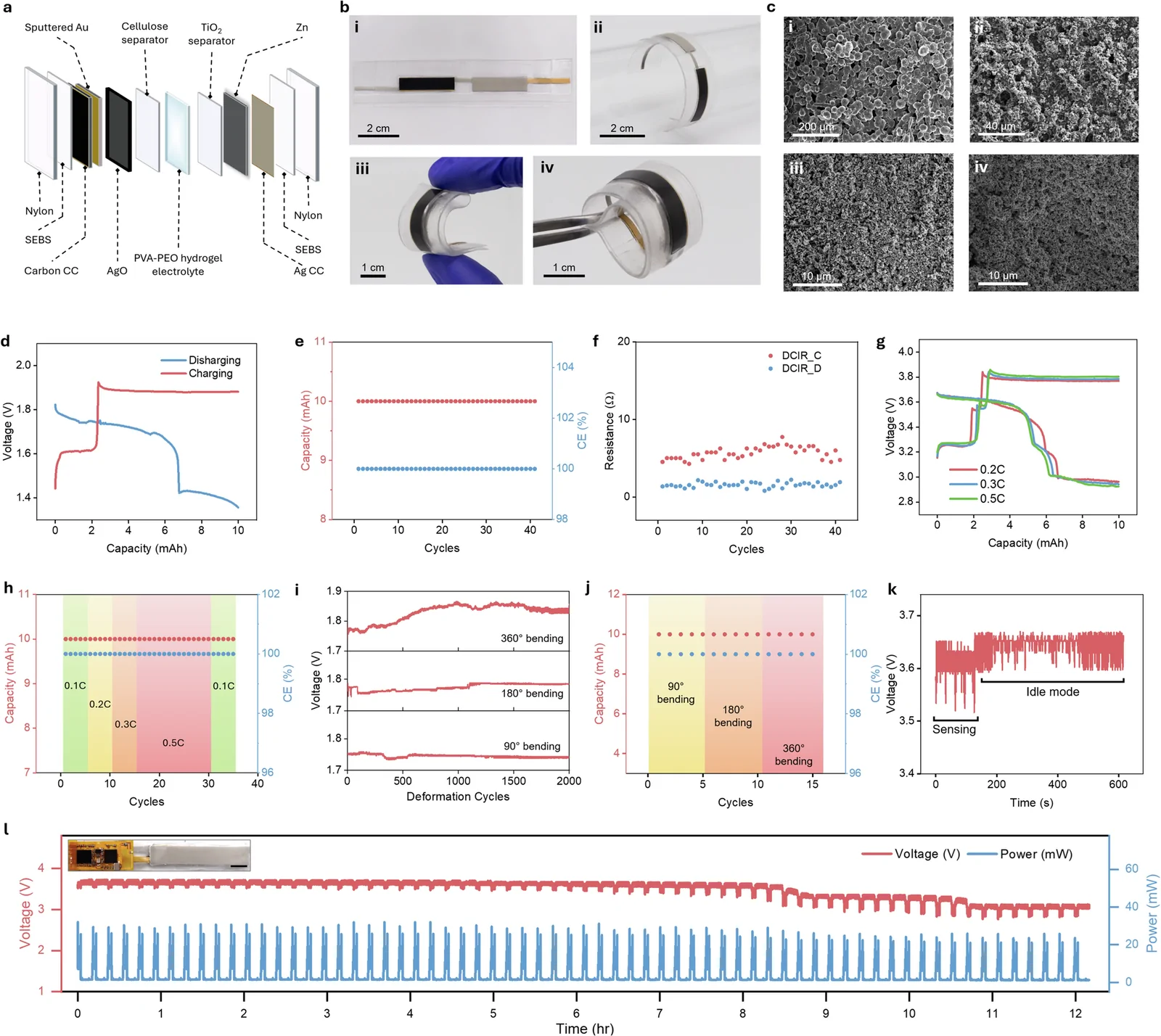
Characterization of the flexible AgO-Zn battery. **a** Exploded view detailing the individual layers of the flexible AgO-Zn battery. Schematic prepared suing MS PowerPoint. **b** Images of the in-series connected AgO-Zn battery under bending angles of 0° (i),90° (ii), 180°(iii), and 360° (iv). **c** SEM images of the Zn anode (i), AgO cathode (ii), TiO_2_ (iii) and cellulose (iv) separators. Each component was finalized after *n* = 3 experiments. **d** The voltage-capacity plot of the battery under *0.1 C* rate. **e** The cycling performance of the battery at charging and discharging rates of *0.5 C*. **f** The DCIR of the AgO-Zn within 40 cycles at the C-rate of *0.5 C*. **g** The voltage–capacity plot of the AgO-Zn flexible battery under different discharging C-rate. **h** The cycling performance of the battery at different charging and discharging C-rates. **i** The battery voltage under repeated different bending angles for 2000 cycles. **j** The battery voltage under bending with continuous galvanostatic cycling. **k** In series-connected battery voltage under discharge of F-PCB system operation across different sensing steps. **l** Energy consumption and voltage response for 12-hour F-PCB operation, along with an inset showing their connection. Scale bar: 5 mm.

The battery was fabricated by screen printing methodology on SEBS substrate with two kinds of current collectors, different separators as well as integrated with a hydrogel-based quasi-solid electrolyte (Fig. 4 a, b and Fig. S25). Screen printing allows the battery design to match the curvature of finger and casing, ensuring proper fit, conformability, and adaptability to varying finger sizes. Thermoelastic SEBS was chosen as the substrate and binder for the current collectors and separator due to its mechanical strength, surface compatibility, and strong interlayer adhesion during printing. The electrode stability is maintained by a high pH-resistant elastomeric fluoro-copolymer binder that also offers high electrochemical durability in the presence of strong oxidative AgO. Furthermore, SEBS and nylon exhibit high resistance to alkaline electrolytes and are compatible with heat sealing, enabling secure containment of the electrolyte. The electrolyte is infused into a quasi-solid PVA-PEO hydrogel to further ensure resilience towards mechanical stresses. The aqueous AgO–Zn battery design provides safe, stable, and reliable operation under mechanical bending while delivering high power output, energy density, and extended lifetime.

The morphology of each fabricated layers and the full cell was characterized using scanning electron microscopy (SEM) (Fig. 4c and Fig. S26). The precursor powders combined with elastomeric binders form flexible composites for each printed layer. SEM imaging after bending showed no fractures (Fig. S27) and preserved surface morphology comparable to pristine electrodes (Fig. 4c and Fig. S26), confirming the binder’s effectiveness in imparting flexibility. SEM images of the TiO_2_ separator layer after 20 cycles show preserved morphology across the three samples, indicating that Zn dendrites were contained and unable to puncture the anode separator due to Bi_2_O_3_ (Fig. S28)^32^. To fit the ring size, the battery was fabricated to range 2.7 cm × 0.5 cm and configured in a two-cell series to deliver over 3.6 V and a rechargeable capacity of 10 mAh (Fig. S29). The involvement of the AgO redox stage enables 2.5 X higher capacity and twice the cycle life compared to a similarly designed battery using an Ag₂O cathode (Fig. S30). The AgO-Zn battery also demonstrated excellent flexibility under bending angles of 90° (bending radius of 16 mm), 180° (bending radius of 8.0 mm), and 360° (bending radius of 4.0 mm) (Fig. 4b), indicating its adaptability for integration into various curvatures. The batteries are folded along the silver wire junction to achieve a size comparable to a single cell and are shaped to fit snugly against the inner right surface of CHARM’s 3D-printed casing (Fig. 4b).

The battery shows two voltage platforms. The first platform is situated at ~1.8 V, corresponding to the reduction of AgO to Ag_2_O, followed by the second plateau at ~1.5 V, associated with the continued reduction of Ag_2_O to Ag (Fig. 4d). The cycle life characterized by galvanostatic charge-discharge at 0.5 C shows a stable 10 mAh capacity and 100% coulombic efficiency over 40 cycles (Fig. 4e and Fig. S31). Two-electrode DC internal resistance (DCIR) was measured during cycling at each charge and discharge current, revealing stable cycle performance with only a minor increase in internal resistance under repeated galvanostatic operation (Fig. 4f and Fig. S31). Two AgO-Zn batteries in series generates a combined potential of 3.6 V and 3.0 V at the first and second voltage platform, respectively, which is sufficient to continuously power the electronics (Fig. 4g). Furthermore, increasing current density showed minimal effect on the voltage of the two batteries in series (Fig. 4g and Fig. S32), and a rechargeable capacity of 10 mAh is maintained across varying current densities (Fig. 4h), enabled by its low internal resistance. It also exhibited a CE of ~100%, demonstrating efficient and reversible electrochemical performance (Fig. 4h).

The mechanical stability of the battery was evaluated by simulating stresses at relevant working conditions. The battery was bent up to 90°, 180°, and 360°, and 2000 bending cycles were applied in each mode. It maintained stable open-circuit voltage (OCV) under all the conditions, reflecting minor voltage variations from mechanical stresses (Fig. 4i). Furthermore, the battery operated under a continuous state of deformation inside CHARM, and stable performance under these conditions was confirmed by cycling at 0.5 °C using a protocol (Fig. 4j and Fig. S33). The protocol consists of cycling the battery under no stress for five cycles, applying a flexure of 90° from cycle 6 to cycle 15, then increasing the flexure to 180° for the following ten cycles, and finally increasing the flexure to 360°. As shown in Fig. 4k, the series-connected battery was discharged during PCB system operation across different sensing steps, with only a slight decrease in OCV. The power consumption profile revealed a low baseline with intermittent peaks, remaining below 40 mW throughout operation (Fig. 4l). Moreover, the energy system maintained an operational voltage above 2.75 V during continuous PCB operation with active sensing and wireless data transmission, ensuring stable CHARM functionality for more than 12 h (Fig. 4l). Finally, the self-discharging analysis during 45 continuous cycles over 8 days showed ~2.74 mAh drop, which does not reduce the total delivered capacity (Fig. S34).

### Analytical performance in type 1 diabetic subject

**Fig. 5:**
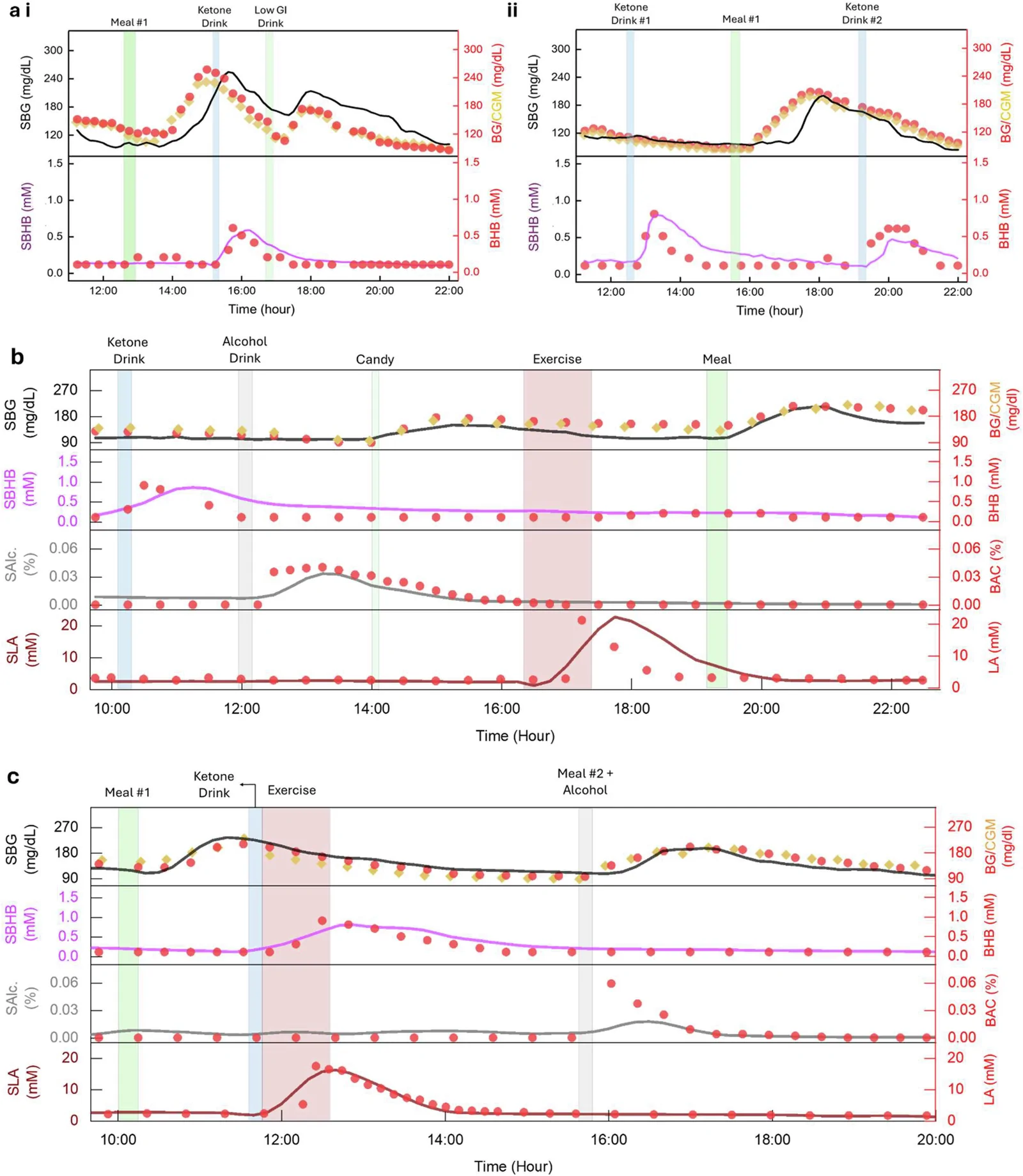
CHARM’s performance with T1-D subject. **a** Plots highlighting glucose and ketone dynamics, validated against blood and commercial CGM readings over a ~ 10-hour study with i) multiple meals and one ketone drink, and ii) one meal with two ketone drinks. To convert current to concentration, $a=0.007\frac{\mu A.{{cm}}^{-2}}{{mg}.{{dL}}^{-1}}$, $b=-0.69{\mu}A.{{cm}}^{-2}$ was used for glucose, while $a=0.074\frac{\mu A.{{cm}}^{-2}}{{mg}.{{dL}}^{-1}}$ and $b=-0.11.{{cm}}^{-2}$ was used for ketone. **b** A 12-hour study capturing all the individualized effects of meals, ketone drink, exercise, and alcohol on the sensor response. **c** A 10-hour study capturing the combined effects of multiple stimuli on the sensor response. The trends in (**b**) and (**c**) were also validated against commercial blood and CGM readings. For all tests, $a=0.007\frac{\mu A.{{cm}}^{-2}}{{mg}.{{dL}}^{-1}}$, $0.77\frac{\mu A.{{cm}}^{-2}}{{mM}}$, $32.38\frac{\mu A.{{cm}}^{-2}}\%$, and $0.117\frac{\mu A.{{cm}}^{-2}}{{mM}}$ was fixed for glucose, BHB, alcohol and lactate, respectively. Fixed value for b ranged −0.69,−0.11, and$-0.21,-0.02\mu A.{{cm}}^{-2}$ for glucose, BHB, lactate, and alcohol, respectively.

CHARM was first tested on a T1-D subject and initially focused on glucose and ketone. We examined whether the measured current at the ring site could be directly translated into concentration values, enabling more intuitive interpretations of diabetic events for users (Supplementary Note 9)^5,33^. We studied the relative change in current and blood concentration from a specific stimuli, and used the following equation (shown here for glucose) to convert I(t) to its equivalent sweat-based blood glucose concentration (${SBG}(t)$):

4 $${SBG}\left(t\right)=\left(\frac{I\left(t\right)-{I}_{i}+\triangle {I}_{0}}{{\triangle I}_{\max }}\right)\left({{BG}}_{\max }-{{BG}}_{f}\right)$$

where, I(t): measured sensor current over time, ${I}_{i}$: current value at t=0 (fasting current), $\triangle {I}_{0}$: initial current change at t=0, ${\triangle I}_{\max }$: maximum current change, ${{BG}}_{\max }$: maximum change in blood concentration from meter, and ${{BG}}_{f}$: fasting blood concentration measured by meter. The two key parameters in this equation: $a=\frac{\triangle {I}_{\max }}{{{BG}}_{\max }-{{BG}}_{f}}$ and $b=-\triangle {I}_{0}$ can be personalized to the subject improve accuracy of ${SBG}(t)$ estimation. Equation 4 can be applied to any biomarker in this study, and its rationale and execution strategy are as follows: a) Coefficients of a and b for every biomarker are empirically derived after considering the sensor current and its maximum concentration change in blood. b) As the flux at the sensor–fluidic channel interface changes continuously over time, parameters obtained from a conventional in vitro calibration curve cannot be directly used to convert current into concentration. Thus, Eq. 4 provides an alternative approach to estimate blood concentration from the sweat current trends. c) Values of a and b will vary between biomarkers as they depend on the biomarker concentration in sweat, sensor composition, and reaction kinetics of the enzymatic reaction. d) a and b values are determined separately for each measurement day using a freshly prepared sensor. Hence, any observed deviation is most likely to arise from individual-specific effects, rather than sensor-related variability. e) Values of a and b can vary across repeated measurements on different days from normal biological fluctuations, physiological state, and environmental factors effects between the periods of measurements. Hence, we check stability of both a and b over multiple days under the same conditions for each individual. Our focus remained on checking the short-term accuracy of CHARM (on a per day basis) over an extended timeline, rather than on the long-term (continuously over multiple days) performance of a single sensor. If deviations occur, the subject may simply recalibrate with blood strips to update the values (Fig. S35). A higher measurement frequency would capture only short-term fluctuations, whereas measurements over extended periods (our approach) are better suited to tracking overall trends and averaging out variations from the factors listed in (c). The calculated ${SBG}(t)$ remains independent of sweat rate, as osmosis effectively minimizes its influence on the sensor response (Fig. 2h). Figure S35 shows the execution of this approach for deriving glucose concentration trends from sweat over time. The average values of a and b for the subject ranged ~ $0.007\pm 0.001\frac{\mu A.{{cm}}^{-2}}{{mg}.{{dL}}^{-1}}$ and ~ $-0.691\pm 0.107\mu A.{{cm}}^{-2}$, receptively for glucose; ~$0.074\pm 0.027\frac{\mu A.{{cm}}^{-2}}{{mg}.{{dL}}^{-1}}$ and $-0.11\pm 0.03\mu A.{{cm}}^{-2}$, respectively for ketone, during independent testing with new patches over multiple days (Figs. S36, 37). These values also showed insignificant change till day 60 (since day 1 calibration). Using these parameters, we tested the subject on day 65 with different glucose and ketone intakes throughout the day (Fig. S38). BG spiked by ~110 mg/dL and ~40 mg/dL from the two meals (with 8U insulin, ~ 0.27 mg). The resulting *SBG (t)* trends aligned well with BG and CGM profiles throughout the testing period. *SBG (t)* from iced-coffee (5U insulin) showed a lag time (${t}_{{lag}}$) ~ 20 min vs. BG peak timeline. Blood ketone (BK) peaked to ~0.8 mM around 60 min after drink while *SBHB (t)*’s peak lagged ~20–25 min against BK. These timelines for BHB align well with our previous findings on fingertip sweat ketone sensing (10-15 mins)^34^. The slightly longer lag time likely stems from the lower sweat gland density at the ring site, which consequently lowers the osmotic extraction rate^35^. ${SBG}(t)$ and ${SBHB}(t)$ showed a mean absolute relative difference (MARD) of ~18.26% and Pr ~ 0.83, respectively, against their corresponding blood concentration values, demonstrating the model’s effectiveness and its utility as a blood-free approach for tracking sweat biomarkers. Using these fixed parameters, we further evaluated the subject under different glucose and ketone intake conditions (Fig. 5ai, ii and Fig. S39). In the first case, we induced two glucose spikes with a meal and a low–glycemic index drink (5U insulin), and with a ketone drink intake in between. Both BG and CGM readings started rising from ~25 min and their peaks appeared ~120–140 min after the first meal (Fig. 5a i). This peak timeline ranged ~45 min from the low GI drink. Such timelines have been previously reported in large T1-D cohort studies, which can range 15–300 min, largely influenced by the meal composition and timing^36,37^. With overnight fasting, the insulin resistance remains high for T1-D subjects in the morning due to different hormonal surges (from the release of cortisol, glucagon, and catecholamines)^38,39^. Due to this, the postprandial blood glucose change ($\triangle {BG}$) from the first meal also ranged high ( ~ 110–150 mg/dL), as seen in Fig S38 and Fig. 5ai. ${SBG}(t)$ aligned well with BG and CGM measurements, showing ${t}_{{lag}}$ ~ 15-minute after the first meal but none following the second, and a MARD of 18.90% against BG. BK increased to 0.6 mM within 30 minutes of the drink, and ${SBHB}(t)$ closely matched its trend with a Pr ~ 0.94. The GKI post drink ranged ~16–17, indicating that the drink did not induce rapid ketosis in presence of meal associated glucose^40^. In the second case, BG, CGM readings and ${SBG}(t)$ did not show any change from the first ketone drink, while $\triangle {BG}$ ranged ~ 100 mg/dL from the late afternoon meal (Fig. 5aii). The lower magnitude of this change compared to that shown in Fig. 5ai can be attributed to the lowered insulin resistance, driven naturally by the human circadian rhythm^39,41,42^. ${t}_{{lag}}$ ranged ~20 min, with a MARD of ~11.30% against BG. BK peaked up to 0.8 mM and 0.5 mM from two ketone drinks (Fig. 5a ii). GKI ranged ~6.5 (fasting) to 11 (post-meal) during peak BK window, indicating a transient phase of nutritional ketosis. Higher morning insulin resistance drives the body to rely more on fats for energy (as glucose cannot be metabolized), leading to increased ketone release into the bloodstream^43,44^. As a result, BK levels were elevated more after the first drink and declined later in the evening, despite an identical intake. ${SBHB}(t)$ followed a similar trend, showing a Pr~0.88. Additional glucose from another diabetic subject is shown in Fig. S40. Overall, these results highlight the importance of tracking extended glucose–ketone dynamics in T1D and demonstrate how CHARM can potentially generate this information effortlessly.

We also tested CHARM for other diabetic biomarkers, like alcohol and lactate (Figs. S41–44). Lactate helps identify oxidative stress and early DKA, while alcohol profoundly disrupts glucose regulation and can trigger delayed hypoglycemia. Hence, together they provide deeper insight into metabolic stability and safety for T1D individuals^8^. Our first testing protocol included evaluating the individual effects of all stimuluses (Fig. 5b). BK spiked up to 0.9 mM within 45 min (GKI ~ 6.6), while ${SHBH}(t)$ peaked within 75 min after drink consumption, with a Pr ~ 0.81. Blood alcohol content (BAC) rose by 0.04% with 250 ml wine intake and displayed a slow clearance pattern, fully returning to baseline in ~4.5 h. Both BAC and sweat based alcohol (${SAlc}(t)$) peaked within 45–60 min, with ${SAlc}(t)$ showing a Pr ~ 0.92. BG showed a drop of ~35 mg/dL within 2 h of alcohol intake. This arises since alcohol is known to induce hypoglycemia through hepatic gluconeogenesis suppression and hepatic glycogen store depletion in T1D subjects^7,45,46^. To prevent progression of hypoglycemia, the subject then consumed candy to elevate blood glucose. Both BG and CGM readings changed by 65-70 mg/dL within an hour, while ${SBG}(t)$ changed by ~40 mg/dL. The variation could arise from the residual alcohol in the bloodstream that might not have been fully cleared. BG and CGM readings peaked within 90-95 minutes after the night meal. ${SBG}(t)$ also peaked around a similar timeline with a MARD of ~15.20% against BG. Lactate was also measured between the two glucose spikes, and the exercise timeline was chosen to minimize the influence of active sweat before the second glucose spike. 30 min of moderate intensity exercise (50–60% maximum heart rate) elevated the blood lactate (BL) to ~20 mM within 15 min. The sweat derived lactate (${SLA}(t)$) ranged similar, with a ${t}_{{lag}}$, MARD, and Pr of ~ 15 min, 26%, 0.85, respectively, against BL.

The combined effects of multiple stimuli were also evaluated (Fig. 5c). The first meal spiked BG, CGM reading, ${SBG}(t)$ by ~ 85 mg/dL, 80 mg/dL, and ~100 mg/dL, respectively after 80 min. ${SBG}\left(t\right)$’s MARD and Pr ranged 12.55% and 0.97, respectively, against BG. The second meal was consumed with alcohol, which caused a lower spike in BG and ${SBG}(t)$, likely due to alcohol’s inhibition of gluconeogenesis. The ketone drink followed by exercise did not cause any notable changes in the BK and $({SBHB(t)})$ profiles, proving there were no dilution effects from active sweat. ${t}_{{lag}}$ and Pr ranged ~ 30 minutes and 0.88, respectively, vs. BK. Alcohol consumed with meal showed a rapid spike in BAC with rapid clearance from blood (in ~1 h). However, ${SAlc}(t)$ showed a delayed rise and increased by only 0.008% ( ~ 2.3X lower than at fasting state) (Fig. 5b). This occurs primarily since food slows the alcohol delivery rate to the small intestine, where most absorption occurs^47^. ${SLA}(t)$ profile remained unaffected by the ketone drink and closely followed BL, yielding a peak concentration, Pr, and MARD of 16 mM, ~0.95, and 18.80%, respectively. The performance of UA and VC sensors is shown in Fig. S45. Overall, these results illustrate that real-time tracking of multiple biomarkers is crucial for informed diabetic management, and that CHARM can reliably generate these needed dynamic insights.

### Nutritional analysis in healthy individuals and operational statistics

**Fig. 6:**
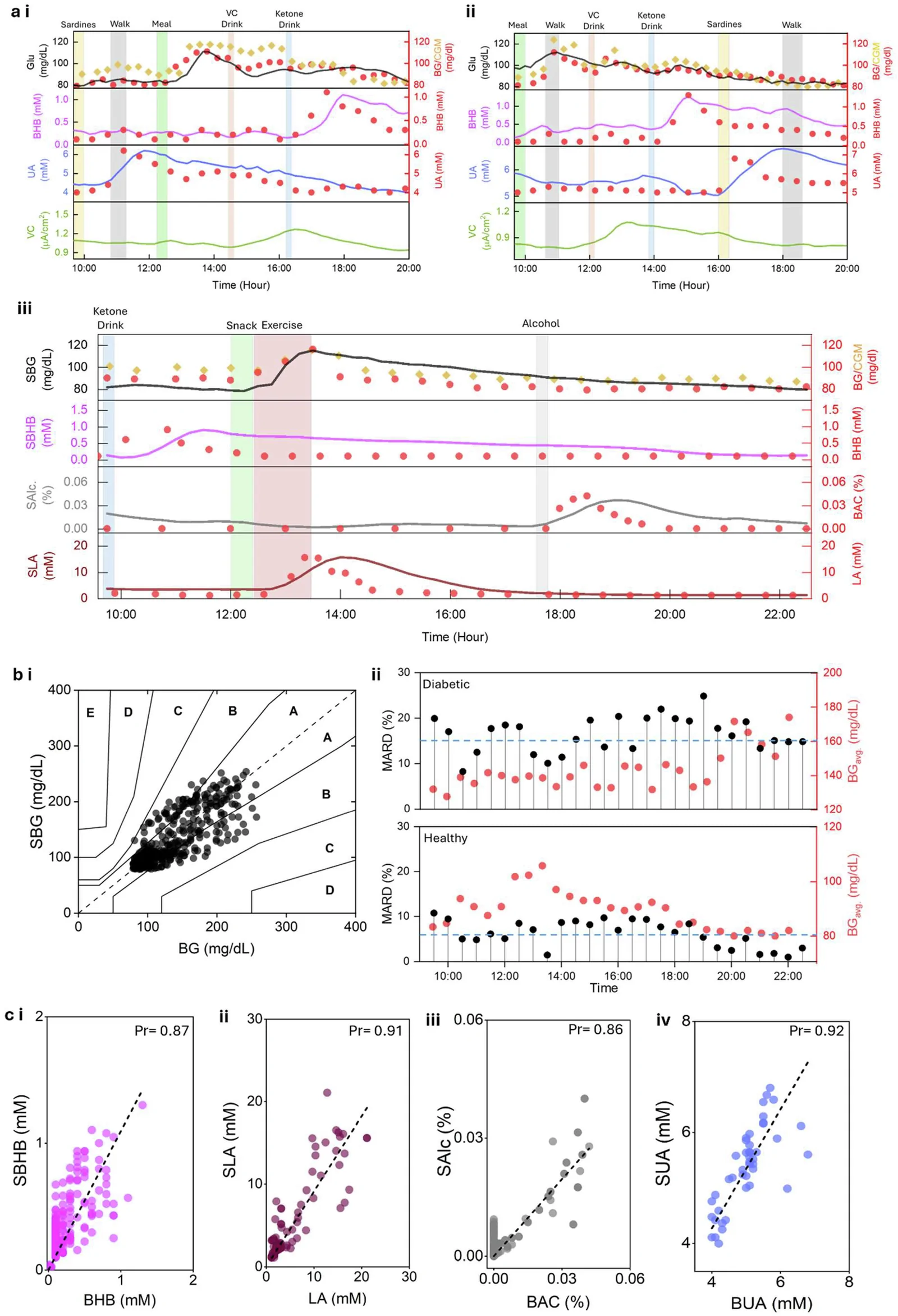
CHARM’s performance for nutritional analysis with operational statistics. **ai–iii** Plots showing the temporal profiles of all six biomarkers measured over a 10–12-hour period in a healthy subject under varying stimuli. For all tests, $a=0.02\frac{\mu A.{{cm}}^{-2}}{{mg}.{{dL}}^{-1}}$, 0.46, .115, $0.132\frac{\mu A.{{cm}}^{-2}}{{mM}}$, and $30.73\frac{\mu A.{{cm}}^{-2}}\%$ was fixed for glucose, BHB, lactate, UA, and alcohol, respectively. Fixed value for b ranged −1.67,−0.084,−0.18, $-0.59\mu A.{{cm}}^{-2}$, and $-0.02\mu A.{{cm}}^{-2}$ for glucose, BHB, lactate, UA, and alcohol, respectively. **bi** CEG plot showing the agreement between estimated ${SBG}(t)$ and true ${BG}(t)$ from both T1-D and healthy subjects tests. Total data points (n)=420. **ii** Plots showing the overall MARD and BG trends over time from all diabetic and healthy test results. Blue dashed line: Mean MARD. **ci–iv** Plots showing the correlation between the estimated blood concentration from CHARM against blood concentration measured by commercial meters for BHB, lactate, alcohol, and UA. *n* = *251* (SBHB), 136 (SLA), 110 (SAlc), and 45 (SUA). Pr was calculated using Origin 2026 plotting software.

CHARM can also deliver nutritional insights through glucose, ketone, UA, and VC measurements (Fig. 6ai–iii and Figs. S46–52). The average value of a for glucose ranged ~2.8X higher than the diabetic subject due to lower BG change under different stimuli. In a healthy individual, meal-spiked BG, CGM, and $({SBG(t)})$ by 30-35 mg/dL within an hour of intake with matching peak timelines. Ketone drink raised BK by 0.7–1 mM, with ${SBHB}(t)$’s showing ${t}_{{lag}}$ and Pr of ~ 30 minutes and 0.77, respectively. However, GKI ranged lower ( ~ 4–5) than the diabetic subject due to a combined effect of intrinsically lower baseline BG, reduced postprandial BG, and similar BK change from the keto drink^40^. Blood uric acid (BUA) and sweat derived uric acid (${SUA}(t)$) changed by 1.5–2 mM with a $\Pr \sim 0.75$ from sardines. However, ${SUA}(t)$’s ${t}_{{lag}}$ ranged ~30–75 min, revealing the slow metabolic rate of dietary purines and slow clearance rate of UA from blood to sweat in the body^10,23,48^. The VC sensor showed a net change of $\sim 0.2-0.3.{{cm}}^{-2}$ 60–90 min after consuming commercial drink. The artificial sweetener present in these drinks also caused ~10 mg/dL changes in BG, CGM, and ${SBG}(t)$ responses. $({SAlc(t)})$ and $({SLA(t)})$ profiles remained similar to diabetic individuals, with a ${t}_{{lag}}$ ranging ~15–20 min vs. their respective peak blood concentration timelines.

All ${SBG}(t)$ values were plotted in a T1-D consensus error grid (CEG) plot to assess its overall accuracy against BG (Fig. 6bi). All data points (*n* = 420) lay in the A + B zone of CEG, confirming that none of them drastically affected the trial outcomes. The overall MARD and Pr against BG ranged ~13.72% and ~0.82, respectively. This MARD value is higher (by 2.88%) than what has been observed at the fingertip with osmotic sweat extraction, confirming higher glucose availability from the sweat at fingertip than ring site^5^. Based on all our tests, the average MARD (blue dashed line) and average BG (${{BG}}_{{avg}.}$) range measured ~17.05% and 140–180 mg/dL, respectively, throughout the day for T1-D population (Fig. 6bii). Higher MARD mainly arises from the wider glucose range and insulin-driven dynamics in diabetes, a trend also widely reported for commercial CGMs under both clinical and at-home settings^49,50^. This was not seen in healthy subjects, and so the average MARD ranged ~6.14%. Moreover, the lowest MARD occurred when rate of $\triangle {SBG}(t)$ ranged -1 to +0.5 ${mg}.d{L}^{-1}/\min$ and -0.5 to +0.2 ${mg}.d{L}^{-1}/\min$ for T1-D and healthy, respectively (Fig. S53). Sweat-derived blood concentrations for all other biomarkers showed good correlation ($\Pr \sim 0.85-0.90$) with their respective blood concentrations, proving CHARM’s high accuracy for daily multi-biomarker monitoring and dependable blood metric assessment (Fig. 6ci–iv).

## Discussion

Despite rapid advances in wearable health technologies, miniaturized devices capable of parallel, long-term monitoring of multiple chemical biomarkers remain limited. Here, we address this gap with CHARM, a smart and compact ring–based wearable for continuous, multi-analyte biochemical sensing. CHARM represents a pioneering integrated biochemical counterpart to existing smart rings, providing a comprehensive evaluation of sweat molecular dynamics at the finger-ring site and establishing it as a viable sensing location. It simultaneously monitors up to four chemical biomarkers for up to 12 hours with osmotically extracted sweat, powered by a flexible, high-capacity Zn–AgO battery with integrated wireless electronics. The platform is safe to be used on skin and exhibits high analytical accuracy with overall glucose MARD ranging ~13.72%, while other biomarkers show strong correlations (0.85–0.90) with their respective blood profiles (Figs. S54, 55). Importantly, the integrated sensor–microfluidic architecture maintains personalized calibration stability for up to two months, eliminating the need for frequent blood-based calibration. Together, these results establish CHARM as a practical, user-friendly multi-biomarker wearable and emphasize the finger ring site as a promising site for daily biochemical monitoring. CHARM can be readily adapted to track additional biomarkers, enabling customization for diverse medical, wellness, and nutritional needs.

Despite the advantages, our study still has several limitations. We believe our design can be further improved to enhance its waterproofing. One possible approach would be to transition from the hinge-lock design to a fully enclosed concentric configuration, in which all components would be arranged along the circumference of the inner circular shell and sealed within a snug elastomeric cover. Protection of the electronic components can be further reinforced with thin elastomeric coatings. While we did not explicitly account for the physiological time lag correction between blood and sweat biomarkers, such lag-correction algorithms can be readily implemented within on-board electronics, as routinely done in commercial CGMs^51,52^. Values of a and b require further evaluation under varying complex daily physiological conditions. The platform has not yet been comprehensively validated in diverse clinical settings and across large diabetic cohorts to assess performance across the full glycemic spectrum (including hypoglycemia, rapid glycemic excursions, and dehydration). Sustained multi-day monitoring will also require further advances in hydrogel engineering for prolonged sweat extraction, biofouling-resistant sensor interfaces, and easily replaceable modular components (such as replaceable sensor-fluidic assembly, rechargeable battery, and PCBs with charging circuits) to enhance user convenience. Future tests with skin-relevant models, such as dermal fibroblasts and keratinocytes, will also further strengthen device biocompatibility. Furthermore, the absence of key vital-sign measurements will be addressed in future designs by developing multimodal chemo-physical hybrid rings. Finally, pathways for clinical adoption - including insurance coverage and reimbursement- remain to be established (from a commercial aspect), similar to existing CGM and insulin pump models. Overall, addressing these aspects will further strengthen CHARM’s capabilities and translational potential and will support its deployment as a practical, user-friendly multi-biomarker wearable monitoring platform.

## Methods

### Chemicals and materials

Phosphate Buffer Solution, Glucose Oxidase, Uricase, Ascorbate Oxidase, Alcohol Oxidase, NAD+ enzyme, HBD enzyme, Chitosan, Glutaraldehyde solution, Acetic Acid, Toludine Blue O, Gold Nanoparticles (15 nm dia. In citrate buffer), Acrylamide, N, N’-Methylenebis(acrylamide), 2-hydroxy-4’-(2-hydroxyethoxy)-2-methylpropiophenone, Poly(vinyl alcohol), Triton-X 114, KOH (90%, flakes), LiOH ( ≥ 98%, powder), Poly(ethylene oxide) (PEO) (MW 600,000), acetone, 4-methyl-2-pentanone (MIBK), Poly(tetrafluoroethylene) (PTFE) (powder, 1 µm particle size), Silver Oxide (AgO) powder, TiO_2_ (anatase, powder, 99.8% trace metals basis), cellulose (microcrystalline, powder), ZnO (powder, <5 μm particle size 99.9%), Bi_2_O_3_ (powder, 10 μm, 99.9% trace metals basis), and Silver flakes (10 μm, 99.9% trace metals basis) were obtained from Sigma-Aldrich.

E2414 Ag/AgCl and E3449 graphite ink were obtained from Ercon. Carbon graphite paste was obtained from Gwent. Carbon paste BG04 (Prussian blue ink) was obtained from Sun Chemical. MD1648 SEBS and G1645 SEBS were obtained from Kraton. Wattman's paper was obtained from Cytiva. Tegaderm 1626 W and PET film were obtained from 3 M. Lactate oxidase was obtained from Toyobo. Multiwalled Carbon Nanotubes (MWCNTs) were obtained from www.cheaptubes.com. GBR 6005 (poly(vinylfluoride-co-2,3,3,3-tetrafluoropropylene)) was obtained from Daikin US Corporation. Ca(OH)_2_ (powder), toluene, and graphite powder were purchased from Thermo Fisher Scientific. Super-P carbon black was purchased from MTI corporation. Zinc powder was obtained from GRILLO-Werke AG.

### Hydrogel preparation

The hydrogel solution comprised acrylamide, N, N′-methylenebisacrylamide (cross-linker), 2-hydroxy-4′-(2-hydroxyethoxy)−2-methylpropiophenone (photoinitiator), DI water, and 0.1 g/mL PVA solution in the mass ratio of 0.0293:0.006:0.0017:0.8375:0.1256.

All gel solutions were poured onto a petri dish and cured under UV light (11.7 mW/cm^2^, 365 nm) for 90 min. The gel was then soaked in pure ethylene glycol (EG) overnight. Disks of 6 mm diameter were then punched out and stored in a vial of fresh pure EG for future use. The prepared hydrogels are not stored beyond 14 days.

### Sensor fabrication

- Screen printing: all six sensors resembled a 2-electrode system. Individual sensors were screen printed on a polyethylene terephthalate (PET) sheet and on SEBS sheet for on-body tests. Current collectors were screen-printed using Ag/AgCl ink. The reference electrode (RE) was screen-printed using Ag/AgCl ink. Lastly, the WE for glucose, uric acid, alcohol, and lactate were screen-printed using Gwent graphite-mediated ink, which is a Prussian Blue-mediated carbon ink. The WE for ascorbic acid was made by screen-printing Gwent carbon ink, and the WE for ketones was made by screen-printing Ercon carbon ink. Both the reference and WEs were printed with sufficient overlap with the current collectors. After each layer was printed, the sensors were cured in the oven at 60 °C for 10 min.

- Drop casting: glucose: the WE of the glucose sensor was functionalized by drop casting three successive layers. 6 µL glucose oxidase solution (40 mg ml^-^1 in 10 mg ml^-1^ bovine serum albumin (BSA)), 1 μL glutaraldehyde solution (1 wt% in DI water), and 1 μL chitosan solution (1 wt% in 0.1 M acetic acid). Additions were performed 1 μL at a time and were dried under a fan at room temperature prior to the addition of the next layer. All sensors used for testing were incubated overnight at ~4 °C.

Uric acid: the WE of the uric acid sensor was functionalized by drop casting 3 successive layers. 5 μL uricase solution (15 mg. ml^-1^ in 10 mg ml^-1^ BSA), 1 μL glutaraldehyde solution (1 wt% in DI water), and 1 μL chitosan solution (1 wt% in 0.1 M acetic acid). Additions were performed 1 μL at a time and were dried under a fan at room temperature prior to the addition of the next layer. All sensors used for testing were incubated overnight at ~4 °C overnight.

Alcohol: the WE of the alcohol sensor was functionalized by drop casting two successive layers. 4 μL alcohol oxidase solution (35 mg ml^-1^ in 10 mg ml^-1^ BSA) and 1 μL chitosan solution (0.5 wt% in 0.1 M acetic acid). Additions were performed 1 μL at a time and were dried under a fan at room temperature prior to the addition of the next layer. All sensors used for testing were incubated overnight at ~4 °C.

Lactate: the WE of the lactate sensor was functionalized by drop casting three successive layers. 9 μL lactate oxidase solution (40 mg ml^-1^ in 10 mg ml^-1^ BSA), 0.5 μL glutaraldehyde solution (2 wt% in DI water), and 2 μL chitosan solution (0.5 wt% in 0.1 M acetic acid). Additions were performed 1 μL at a time (except GA) and were dried under a fan at room temperature prior to the addition of the next layer. All sensors used for testing were incubated overnight at ~4 °C.

Ascorbic acid: the WE of the ascorbic acid sensor was functionalized by drop casting 3 successive layers. 2 μL ascorbic acid oxidase solution (0.55 mg ml^-1^ in 10 mg ml^-1^ BSA), 0.5 μL glutaraldehyde solution (1 wt% in DI water), and 0.5 μL chitosan solution (1 wt% in 0.1 M acetic acid). Additions were performed 1 μL at a time (except GA and chitosan) and were dried under a fan at room temperature prior to the addition of the next layer. All sensors used for testing were incubated overnight at ~4 °C.

Ketone: the WE of the ascorbic acid sensor was functionalized by drop casting 4 successive layers. 2 μL gold nanoparticles (avg dia.) dried at 60 °C for 10 min. 2 μL of Toluidine Blue O (TBO) solution (2 mM TBO and 5 mg/mL MWCNTOOH in 0.5 wt% chitosan in 0.1 M acetic acid) dried at 60 °C for 30 min. 3uL of 1:2 HBD:NAD^+^ solution was drop casted 1 μL at a time. And 1 μL chitosan solution (1 wt% in 0.1 M acetic acid) for in-vitro testing and 2 μL chitosan solution for on-body testing. After each addition of enzyme solution and chitosan, the sensor dried under a fan at room temperature. All sensors used for testing were incubated overnight at ~4 °C. All in-vitro tests were conducted with PalmSens 4, PalmSens EmStat 3, and Autolab (PGSTAT101).

### Sensor-fluidic patch assembly

An electrode array (4 working and 1 reference electrode) was prepared on SEBS. A piece of SEBS sheet (9 × 2 cm) was cut, and a ¼” hole was punched at the top to form the patchy inlet. The paper channel was placed on the SEBS sheet by centering the inlet of the channel to the ¼” hole. Subsequently, the prepared electrode array was placed onto the area of the paper channel between the inlet and the repeating jigsaw pattern. A weight was placed onto the subassembly for at least a minute to ensure the pieces were secured and aligned. Afterward, the weight was removed, and a piece of Tegaderm was placed on top of the subassembly to seal the paper channel and sensor; any remaining excess Tegaderm or SEBS was trimmed away. The PET on the back of both pieces of SEBS was then removed. Lastly, the patch was folded into alternating folds to be inserted into the ring. Double-sided tape was placed in between the folds to compact the patch further. The hydrogel was inserted into the inlet hole and loaded onto the ring.

### Battery fabrication

- Preparation of the SEBS substrate for battery: MD1648 SEBS was dissolved in toluene with a weight ratio of 1 to 1.5 and was left on an orbital mixer (Joanlab OS-20Pro) overnight until the mixture became homogeneous to prepare the MD1648 SEBS resin. PET film was used as the temporary casting substrate, and a doctor blade was used to cast the SEBS resin onto the PET substrate. The newly cast SEBS was left in the fume hood to cure in the ambient environment for 24 h. The flexible, stretchable, and clear SEBS film can be readily peeled off from the PET film after curing, was used as the substrate for subsequent screen printing.
- Preparation of the Quasi-Solid Gel electrolyte for battery: the quasi-solid gel electrolyte was fabricated as described in a previous study^32^. The hydroxide solution was prepared by first dissolving 0.342 g LiOH and 9.426 g KOH in 50 mL DI water. Then, 0.5 g Ca (OH)_2_ was added, and the solution was stirred for 1 h so that the solution is saturated with Ca (OH)_2_. Finally, the solution is filtered through filter paper to remove excess Ca (OH)_2_. The PEO-PVA solution was fabricated by dissolving 4.033 g PVA and 0.056 g PEO into 50 mL of DI water through heating to 90 °C. The precursor solution of the gel was prepared by adding the hydroxide solution to the PVA-PEO solution in a 13.677:10 weight ratio for a total weight of 0.2 g per cm^2^ petri dish area. The hydroxide solution was added drop by drop while the PVA-PEO solution was being stirred. The gel was then dried in vacuum until the gel reaches 26.12% of its original weight. Then, the gel was cut into the same size as the screen-printed active material. The cut gel was soaked overnight in the electrolyte solution (prepared with 0.391 g LiOH, 10.777 g KOH, and 0.5 g Ca (OH)_2_ in 50 mL DI water in the same way as the hydroxide solution) at 4 °C in a refrigerator. The quasi-solid gel electrolyte fabricated was then stored in the electrolyte solution at 4 °C.
- Fabrication of the AgO-Zn flexible battery: the carbon current collector powder was formulated by mixing graphite, Super-P, and PTFE powder in 84:14:2 ratio by weight with mortar and pestle. Then, the carbon current collector powder was mixed with the MD1648 SEBS resin and toluene in a 10:12:3 ratio by weight using a planetary mixer (Flaktak Speedmixer™ DAC 150.1 FVZ) for 10 min at 2500 rotations per minute (rpm) to fabricate the carbon current collector ink. The AgO cathode ink was formulated by first mixing AgO and Super-P powder in a 95:5 weight ratio with mortar and pestle and then mixing the powder with the cathode binder (5 g of GBR 6005 dissolved in 10 g of MIBK by shaking on an orbital mixer) and MIBK in a weight ratio of 5:5:2 for 10 minutes at 2500 rpm. The cathode separator powder was similarly prepared by mixing TiO_2_ and cellulose in a 26:9 weight ratio using mortar and pestle. After that, the cathode separator powder, cathode binder, and MIBK were mixed in a weight ratio of 8:7:4 with a planetary mixer for 10 min at 2500 rpm to prepare the cathode separator ink.
  On the anode side, the silver current collector ink was prepared by mixing 3 g of silver flakes, 1.2 g G1645 SEBS resin (4 g of G1645 SEBS dissolved in 10 mL of toluene by shaking on an orbital mixer), and 0.6 mL toluene with a planetary mixer for 5 min at 1800 rpm. The Zn anode powder was prepared by evenly mixing Zinc, Bi_2_O_3_, and ZnO powder in 18:1:1 weight ratio by a set of mortar and pestle. Then, the Zn anode ink was formulated by mixing the Zn anode powder with the anode binder (25 % GBR 6005 by weight dissolved in acetone) in a weight ratio of 40:11 using a planetary mixer for 10 min at 2500 rpm. The anode separator powder was prepared by mixing TiO_2_ and cellulose powder in a weight ratio of 2:1 with a set of mortar and pestle, and the anode separator ink was formulated by mixing the anode separator powder, G1645 SEBS resin, Toluene, and Triton-X in a weight ratio of 50:55:75:3 with a planetary mixer for 10 min at 2500 rpm.
  The screen-printing technique was used for the layer-by-layer fabrication of the battery. Each layer was printed using a squeegee and a custom-designed stencil with a thickness of 0.005 inches. For each single battery, the anode side and cathode side were printed separately onto their respective current collector, each in the order of current collector, electrode, and then separator; the two layers were combined with the gel electrolyte in the middle in the following battery assembly. To fabricate the cathode current collector, one layer of carbon ink was first printed onto the MD1648 SEBS substrate and cured at 40 °C in a conventional oven for 24 h. PET masks were prepared by cutting PET films into the shape of the carbon current collector using a computer-controlled cutting machine (Cricut Maker®, Cricut, Inc., South Jordan, UT, USA). The PET masks were then applied to the carbon current collector to expose the current collector and shield the surrounding SEBS surface. The masked carbon current collector was sputtered with Ti for 2 min as an adhesion layer and Au for 20 min to increase its conductivity. Both layers were sputtered at a DC power of 100 W and an Argon gas flow rate of 13 SCCM using Denton Discovery 635 Sputter System (Denton Discovery 635 Sputter System, Denton Vacuum, LLC, Moorestown, NJ, USA). Multiple layers of the AgO cathode ink were printed until the total mass of AgO cathode exceeds 97 mg, which ensures a full capacity higher than 20 mAh and a stable cycling capacity of 10 mAh. Each layer was dried under ambient environment before the following layer was printed. One layer of the cathode separator ink was printed so that the cathode separator completely covers the AgO cathode and current collector layers. The cathode separator layer was then dried under ambient conditions.
  To fabricate the anode current collector, one layer of silver current collector ink was printed onto the SEBS substrate and dried under ambient conditions. Then, the Zn anode ink was screen printed onto the silver current collector for multiple layers until the anode mass exceeds 223 mg. Similarly, each layer was dried under an ambient environment before the following layer was printed. One layer of the anode separator was printed to ensure that the current collector and active material layers were completely covered. Then, the cathode separator layer was dried under ambient conditions.
  After the cathode and anode were dried, the cathode was soaked for 40 min, and the anode was soaked for 10 min. The battery is assembled by first stacking the anode, gel electrolyte, and cathode. Then, the SEBS substrate on three sides of the battery were heat sealed, and the final side was vacuum sealed. Nylon was wrapped around the battery as it was sealed to enhance its structural stability.
  To fabricate two cells in series, a silver current collector and a carbon current collector were screen printed in a linear layout with a gap on the same piece of SEBS substrate, with their long sides lined up and their tabs on opposite sides. As the other piece, a silver current collector and a carbon current collector, both without tabs, were screen-printed in the same layout and gap width on another piece of SEBS substrate, with a silver wire then printed across the gap to electrically connect the two current collectors. Then, sputtering, screen printing and assembly steps were carried out in the same way as in the fabrication of single batteries. Overall, the current battery uses non-toxic components and exhibits stable anode and cathode performance. Electrochemical Characterization: after fabrication, the battery was left idle for 16 h for the electrolyte to fully permeate the electrode structure. Cycling tests were done using a computer-controlled battery testing system (Landt Instruments CT2001A). The conditioning cycles applied for single batteries are as follows: first, the battery was discharged 60% of its designed full capacity at 0.2 C. Then, the battery charged 50% of its designed full capacity with constant current charging at 0.2 C until reaching 2 V and constant voltage charging at 2 V. The conditioning cycles for 2 batteries in series are similar the same except for applying constant voltage charging after reaching 4 V. The conditioning cycles were applied to make sure the battery functions between 40% and 90% of its full capacity to ensure a long cycle life. The cycling test was conducted by running the battery through galvanostatic cycles with the desired C rate with 10 min of rest between each step. The cut-off voltage was set to be 1.2 V for discharging steps and 2.05 V for charging steps for single batteries, and the voltages were doubled for 2 batteries connected in series. Direct current internal resistance (DCIR) tests were applied before each discharge and charge step using the same battery testing system. Bending of the battery was applied by conforming the battery to cylinders of various diameters. Active bending was applied using a computer-controlled linear motor (ZABER™ LHM150A-T3) on single batteries at a speed of 1 bending cycle every 4 seconds for 90°, 1 bending cycle every 8 seconds for 180°, and 1 bending cycle every 11 seconds for 360° bends. The voltage was recorded on the same computer-controlled battery testing system. The energy consumption and voltage response analysis was conducted by firstly obtaining the current profile during the operation of the electronics. Then, key characteristics of the measured current data were extracted and translated into a galvanostatic discharging procedure with varying current on Nova 2.1 (Metrohm Autolab). A potentiostat/galvanostat (μAutolab Type III) was used to deploy the procedure to the battery and collect the voltage response, along with the power consumption, from the battery.
- Microstructural characterization methods: the morphology of different layers of the battery before and after bending was observed by scanning electron microscopy (SEM). Pristine samples were cut off from battery layers before assembly. The bent samples were cut off from the same battery layers after being manually bent 180° around a cylinder for 100 bending cycles. SEM Pictures were taken on a Thermo Fisher Apreo SEM equipment with a current of 0.10 nA and an accelerating voltage of 5 kV.

### Integrated ring assembly

CHARM’s casing was designed in AutoCAD and 3D-printed (using Formlabs 4 printer). A Cr adhesion layer was first deposited by sputtering at 100 W for 5 min. This followed a sputtered Pt layer at 100 W for 15 min. The deposition was performed under an argon flow rate of 14 sccm using direct current (DC) mode on a Denton Discovery 18 sputtering system. The sensor and fluidic assembly were first attached to one half of the ring casing. The battery was then attached to the F-PCB and its electrical connections interfaced the connections of the flexible connection pad on one end. This whole assembly was first mounted onto the other half of the ring body and bent such that the electrical connections on the other end of the connection pad interfaced the screen-printed electrode array (as shown in Fig. 1c). The ring was subsequently closed and secured, and ready for wear.

### On-body testing protocol

- Ethics: every experiment involving human participants have been carried out following a protocol approved by an ethical commission. Each participant gave informed written consent. No personal identifiable information was collected.
- Dye testing on skin: all on-body studies were conducted under approved IRB (UCSD IRB# 13003). Two SEBS sheets were prepared, one with a ¼″ hole aligned to the inlet of a paper channel, which was encapsulated by sealing a second SEBS sheet on top using double-sided tape after removing the PET backing. A red dye solution (5 µL dye in 500 µL ethanol) was prepared, and 6 µL was applied to the paper-channel inlet in 1 µL increments with drying between additions to prevent migration. A hydrogel was then placed between the channel inlet and the top SEBS layer, and double-sided tape with a 5/16″ hole was aligned to the ¼″ hole on the underside of the patch. The assembled patch was applied to the palmar side at the base of each finger, and images were collected hourly to monitor dye transport. Dye travel distance was quantified in ImageJ by scaling images using the 6.5 mm inlet diameter and measuring the traced dye path.
- Extended study: prior placing the ring, the subjects washed the ring site with soap and water. Both healthy and T1-D subjects consumed carbohydrate ( ~ 37-40 gm), ~8 gm ketones, ~142 gm sardines, and 250 ml wine for glucose, BHB, UA, and alcohol elevation. Exercise intensity ranged ~50-60% maximum heart rate for lactate elevation. The following commercial blood meters were used for testing: Glucose (Accu Check Guide Me, Roche), Lactate (THE EDGE), BHB (Medilax), Alcohol (BACtrack S80, BACtrack), and UA (UA Sure II, UA Sure). Stelo (Dexcom) was used as the CGM on healthy patient.

### In vitro cytotoxicity evaluation

The cytotoxicity of seven components in CHARM were evaluated by incubating THP-1 cells obtained from the American Type Culture Collection (ATCC, catalog no. TIB-202), originally isolated from the peripheral blood of a male patient with acute monocytic leukemia. The cells were suspended in RPMI medium (Thermo Fisher, Waltham) and adjusted to 1 × 10^6 ^mL^-1^. All of the sample surfaces were sanitized and underwent an 1 h UV sterilization before the 24 h incubation. At 1 h and 24 h, 500 μL THP-1 cells were examined by adding 50 μL of the MTS kit (Colorimetric, ab197010) for each sample group and the untreated control, and was placed at 37 °C for 40 min. The relative cell viability percentage results were acquired by UV spectroscopy (BioTek Synergy Mx microplate reader) at 490 nm absorbance. In addition, the cells are tested with SYTOX^TM^ Green Dead Cell Stain (Thermo Fisher Scientific) at day 7. The corresponding relative cell viability was subsequently analyzed under the same UV spectroscopy microplate reader under 488/525 nm for excitation/emission wavelength, with fluorescence merged images acquired with Invitrogen EVOS XL Core Imaging System (Thermo Fisher Scientific).

### Reporting summary

Further information on research design is available in the Nature Portfolio Reporting Summary linked to this article.

## Supplementary information

Supplementary Information

Description of Additional Supplementary Files

Supplementary Video 1

Supplementary Video 2

Reporting Summary

Transparent Peer Review file

## Source data

Source Data

## Data Availability

All data supporting the findings of this study are available within the article and its supplementary files. Any additional requests for information can be directed to, and will be fulfilled by, the corresponding authors. Source data are provided with this paper. Source data are provided in this paper. The data are not deposited in a public repository because of their extensive nature and the contextual explanation required for appropriate interpretation. Source data are provided with this paper.

## Code availability

The code for the multichannel potentiostat is available: https://github.com/tamoghnasaha91/CHARM-Ring_June-2026
