# Supplementary material for: A fully integrated smart ring for daily biochemical monitoring
Source: Nat Commun. 2026 Jul 23;17:9003. doi: 10.1038/s41467-026-75980-z (PMC13507133; doi:10.1038/s41467-026-75980-z)
Supplement: Supplementary file 1 [file 41467_2026_75980_MOESM1_ESM.pdf]

Supplementary Information

A Fully Integrated Smart Ring for Daily Biochemical Monitoring

Tamoghna Saha[a*], Shichao Ding[a*], Siyu Qin[b*], Hannah Fishman[a], Rosie Pham[c], Te Gu[a], Muyang Lin[a], Yushan Xian[b], Barak Sabbagh[a], Abdulhameed Abdal[d], Gaoyuan Ji[b], Kittiya Sakdaphetsiri[a], Yi Fung Winsten Lam[a], Muhammad Inam Khan[a], Hao Luan[a], Zongnan Wang[d], Selene Tang[a], Omeed Djassemi[b], Samar Singh Sandhu[a], Sheng Xu[a,b,c], Joseph Wang[a#]

[a] Aiiso Yufeng Li Family Department of Chemical and Nanoengineering, University of California San Diego, La Jolla, CA 92093, USA

[b] Department of Electrical and Computer Engineering, University of California San Diego, La Jolla, CA 92093, USA

[c] Department of Bioengineering, University of California San Diego, La Jolla, CA 92093, USA

[d] Department of Mechanical Engineering, University of California San Diego, La Jolla, CA 92161, USA

[*]Equal Contribution

[#]Corresponding Author: josephwang@ucsd.edu

Table of Contents

Table S1: CHARM's comparison vs. sweat devices tested at the ring location

Figure.S1: CHARM assembling process.

Figure.S2: Optical image of the fluidic channel showing liquid advancement at 150 nl/min flow rate.

Supplementary Note 1: Theoretical estimation of hydrogel osmotic pressure

Figure.S3: G'/G'' vs. strain rate plot of hydrogels at angular frequency of 10 rad/s.

Figure.S4: Optical image of the fluidic channel showing greater dye penetration due to osmosis vs. natural perspiration.

Supplementary Note 2: Von Mises stress estimation on hydrogel surface.

Figure.S5: Mechanical simulation of the hydrogel under bending.

Figure.S6: In-vitro performance of the glucose sensor.

Figure.S7: In-vitro performance of the ascorbic acid sensor.

Figure.S8: In-vitro performance of the uric acid sensor.

Figure.S9: In-vitro performance of the ketone sensor.

Figure.S10: In-vitro performance of the lactate sensor.

Figure.S11: In-vitro performance of the alcohol sensor.

Supplementary Note 3: Numerical simulation of analyte flux under varying concentrations and sweat rates

Figure.S12: Schematic illustration and mathematical formulation of the fluid flow numerical model.

Table S2. Parameters, symbols, and values used in the fluidic numerical simulation.

Figure.S13: Mesh Convergence and Numerical Validation.

Figure.S14: Transient numerical simulation showing time-dependent inlet concentration changes.

Supplementary Note 4. Electronic system overview and control architecture.

Supplementary Note 5. Debug and deployment configurations.

Supplementary Note 6. Power consumption measurement methodology 1.

Supplementary Note 7. Power consumption measurement methodology 2.

Supplementary Note 8. Power consumption measurement methodology 3.

Figure.S15: Circuit schematics.

Figure.S16: F-PCB layer and drill layout.

Figure.S17: Bill of materials.

Figure.S18: Low-power control flow chart.

Table S3: Effect of supply voltage on chronoamperometric current measurement.

Figure S19: BLE data acquisition and transmission workflow.

Figure S20: DC bias resistor function

Figure S21: Reconnection after sleep.

Figure S22: RSSI measurement under different device configurations.

Figure.S23: Chronoamperometric response from 4 channels in the F-PCB, operating sequentially for peroxide detection in a Prussian blue-carbon electrode system.

Figure.S24: Chronoamperometric response from 4 channels in the F-PCB, operating sequentially for glucose, VC, UA, and BHB detection at their respective potentials.

Figure.S25: Optical images of the layer-by-layer printing of the flexible AgO-Zn battery.

Figure.S25: SEM images of the current collectors and layers of the AgO-Zn battery.

Figure.S27: SEM images of the different layers of the flexible AgO-Zn battery after bending.

Figure.S28: SEM images of the surface of the TiO_2 separator in three different battery samples after 20 cycles.

Figure.S29: The voltage-capacity plot of the in series connected batteries.

Figure.S30: The voltage-capacity plot of the battery under varying charge and discharging rates.

Figure.S31: The charging capacity and coulombic efficiency of two other battery samples.

Figure.S32: The voltage-capacity plot of 3 different battery samples charging and discharging under different C-rates.

Figure.S33: The voltage-capacity plot of the battery under bending.

Figure S34: Self-discharge characteristics of the battery.

Supplementary Note 9: Calibration model to acquire blood concentration dynamics from sensor's $I(t)$.

Figure.S35: Flow chart of personalized calibration protocol for glucose.

Figure.S36: Glucose profile of T1D subject over multiple days.

Figure.S37: Ketone profile of T1D subject over multiple days.

Figure.S38: Utilization of the personalized calibration protocol to convert current to concentration in a daily test.

Figure.S39: Current profiles of glucose and ketone related to Figure.5a i and ii.

Figure S40: Glucose profile of the second T1D subject over multiple days

Figure.S41: Lactate profile of T1D subject over multiple days.

Figure.S42: Alcohol profile of T1D subject over multiple days.

Figure.S43: Current profiles of glucose and ketone related to Figure.5b.

Figure.S44: Current profiles of glucose, BHB, alcohol and lactate from T1D subject related to Fig.5c.

Figure.S45: UA (blue) and VC (green) profile of T1D subject over multiple days.

Figure.S46: Glucose profile of healthy subject over multiple days.

Figure.S47: BHB profile of healthy subject over multiple days.

Figure.S48: UA profile of healthy subject over multiple days.

Figure.S49: Lactate profile of healthy subject over multiple days.

Figure.S50: Alcohol profile of healthy subject over multiple days.

Figure.S51: Current profiles of glucose, AA, UA and BHB from T1D subject related to Fig.6a.

Figure.S52: Current profiles of glucose, BHB, alcohol and lactate from the healthy subject related to Figure.6a.

Figure.S53: Comparison of MARD vs. Rate of $SBG\ (t)$ change.

Figure.S54: Cytotoxicity test of CHARM's components.

Figure.S55: Multiday on-skin tolerance test of CHARM.

Table S1: CHARM's comparison vs. sweat devices tested at the ring location.

Component	Parameter	This work	vs. Tu et al.,[1]	vs. Cui et al.[2]	vs. Fu et al.[3]
**Form factor**	N/A	Fully integrated true ring	Flexible patch	Flexible patch wrapped at ring location	Flexible patch wrapped at ring location
**Microfluidics**	Material	Paper	PDMS	PDMS	PDMS
	Accommodating volume	~90 µL Sensing volume: 5 µL	~ 21 µL	~7 µL	Not reported
	Size	8 cm length 3D stackable	~ 2 cm length – No 3D stackable	~1.5 cm length – No 3D stackable	~1.5 cm length – No 3D stackable
	Inlet	5 mm diameter	0.2 mm diameter	5 mm diameter	6 mm diameter
	Mixing chamber	No	0.8 mm diameter	No	No
	Outlet	7 mm diameter	0.1 mm diameter	Not reported	0.5 mm diameter
**Battery**	Form factor	Printed flexible battery	Coin cell	Coin cell	Coin cell
	Voltage	3.6 V	3.8 V	3.8 V	3.8 V
	Capacity	10 mAh	8 mAh	8 mAh	8 mAh
**Sensors**	Biomarker	Glucose, lactate, ketone, alcohol, uric acid, ascorbic acid	C-reactive protein, pH, sweat rate	Estradiol, pH, sweat rate	C-reactive protein, cholesterol, potassium, sweat rate
**Sweat Access**	Technique	Osmotic (passive)	Iontophoresis (active)	Iontophoresis (active)	Iontophoresis (active)
	Sweat rate	0.1-0.2 µL/min	0.5-3.5 µL/min	Not reported	Not reported
**Operation**	Sensing duration	~12 hours	60s scans	10s discrete scans over multiple days	Discrete scans

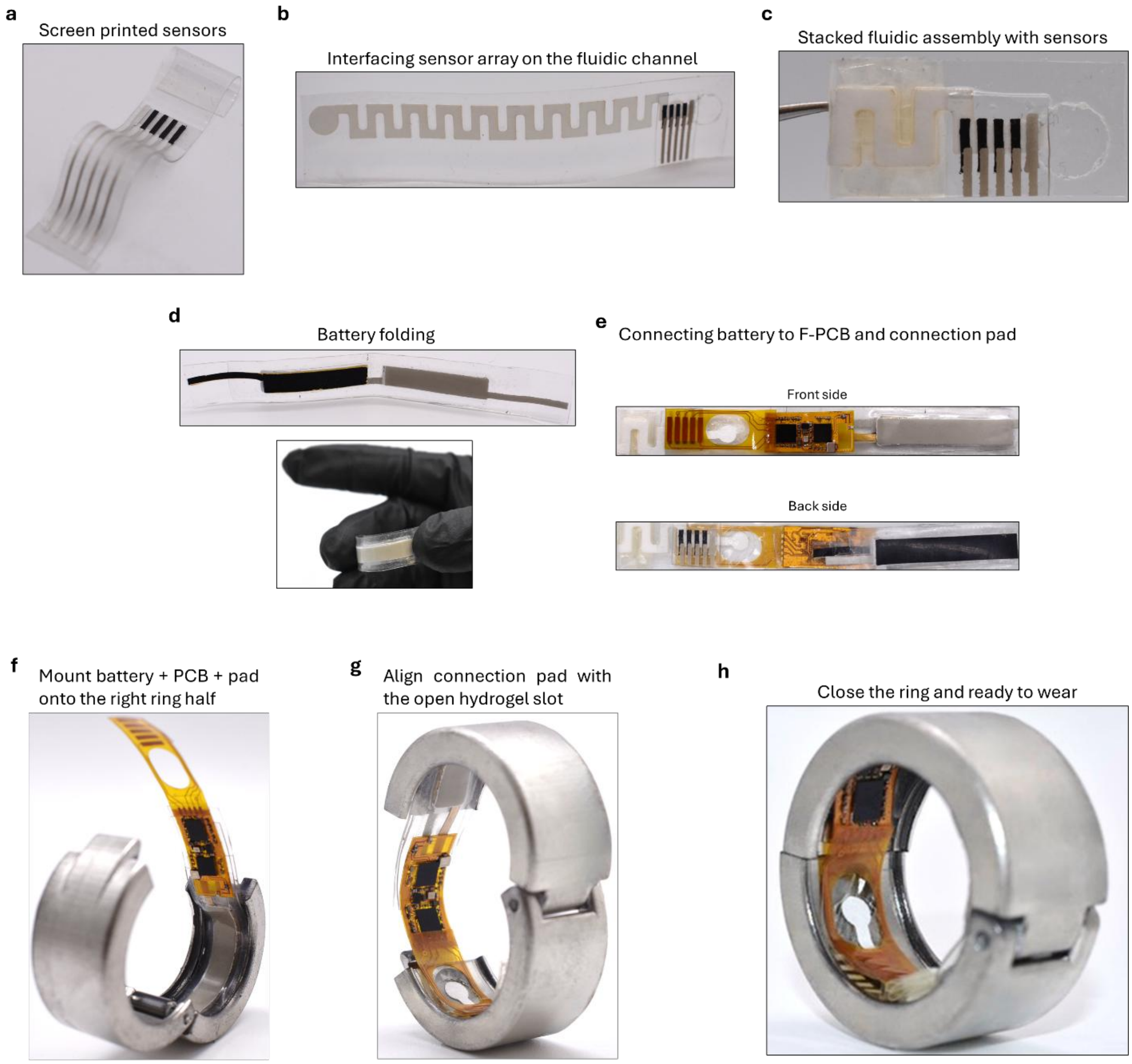

Fig. S1: CHARM assembling process. a) The electrode array are first screen printed on SEBS and b) interfaced to the fluidic channel near the hydrogel inlet with SEBS casing. SEBS casing prevents all fluid evaporation**.** c) The fluidic channel is then folded to reduce its length such that it fits in the ring. d) The printed batteries are folded along the center and e) connected to the F-PCB on one end. The other end of F-PCB connects to the electrical connections of the connection pad. The pad has an elliptical slot to accommodate the hydrogel. Metallic array connections on the other end of the connection pad connects to the printed electrochemical sensor array. f) The whole assembly is fitted on the right halve of CHARM and curved such that the elliptical hole on the connector pad aligns with the hydrogel slot on the left halve of CHARM. The right half is pushed such that it locks, making CHARM ready for use.

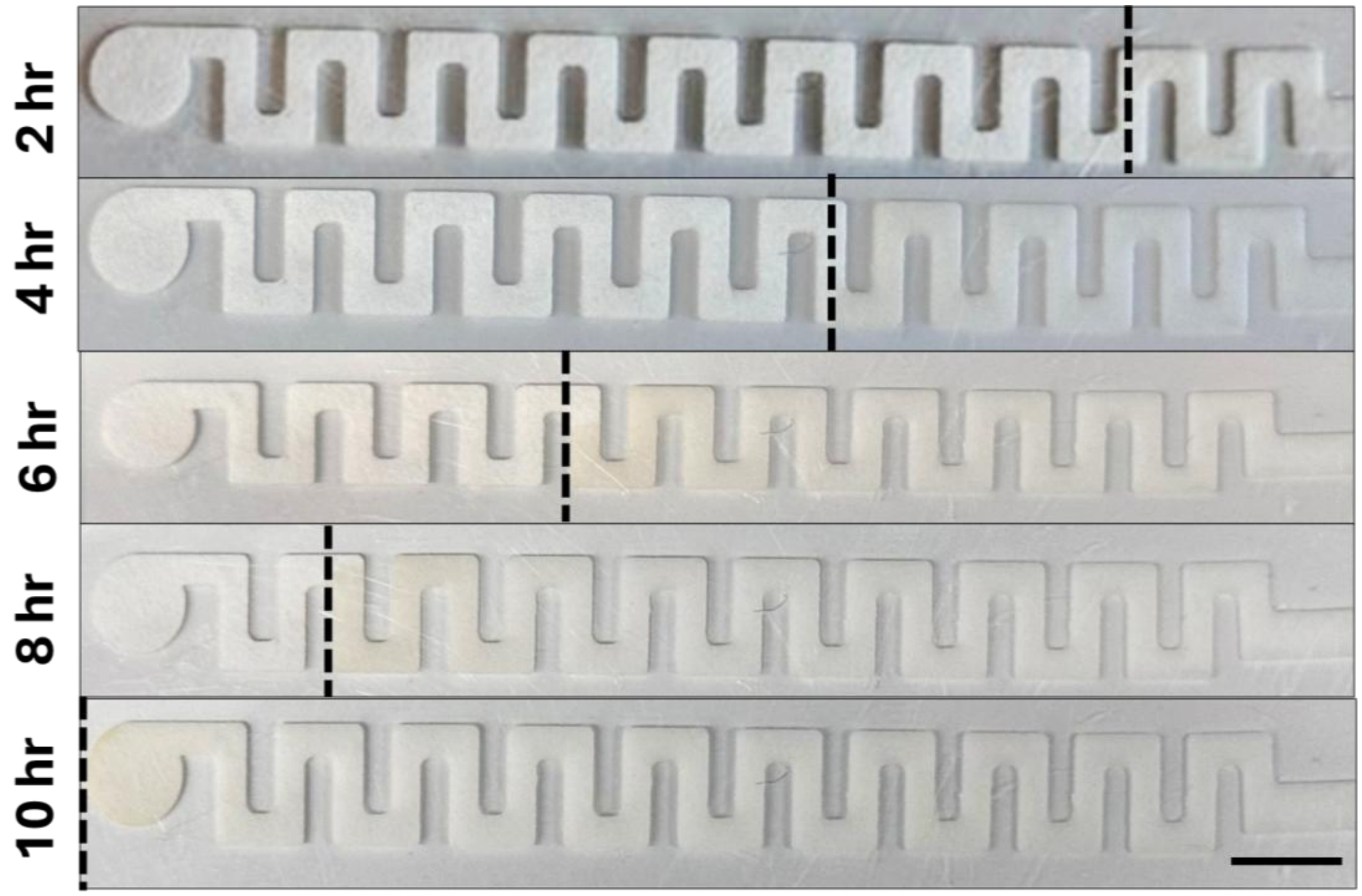

Fig. S2: Optical image of the fluidic channel showing liquid advancement at 150 nl/min flow rate. The fluid reaches the end of channel in about 10 hours. Total volumetric capacity ~ 85-90 µL's**.** Scale bar: 5 mm.

Supplementary Note 1: Theoretical estimation of hydrogel osmotic pressure

The osmotic pressure of a hydrogel is derived theoretically by estimating the swelling pressure using the Flory-Rehner Theory. The swelling capacity of any gel system upon fluid intake is highly dependent on the overall contributions from elastic forces, polymer-solvent interactions, and ionic composition. These effects depend on several thermodynamic properties (*e.g.,* temperature, pH, solution ionic strength and composition). Hence, it is important to estimate each contribution to understand the overall swelling behavior and fluid extraction capacity of a hydrogel.[4–7]

The total swelling pressure (π_{tot}) is derived using the following equation:

$$\pi_{tot} = -V_1^{-1}\left(\frac{\partial \Delta F_{tot}}{\partial n_1}\right) = \pi_{el} + \pi_{mix} + \pi_{ion} \quad (2)$$

where V_1 is the solvent molar volume (18 ml/mol), n_1 is the number of moles of solvent, π_{tot} is the total gel swelling pressure and π_{el}, π_{mix} and π_{ion} are the elastic, mixing and ionic contributions to the total swelling pressure, respectively.

We first quantified the elastic contributions (π_{el}) for all our hydrogel systems. For every hydrogel variant, we estimated its weight swelling ratio (q_p), polymer volume fraction after equilibration ($\emptyset_2$), and gel shear modulus (G) via experiments. These parameters were used for the estimation of π_{tot}. The q_p was calculated as:

$$q_p = \frac{m_{gel}}{m_{dry}} \tag{3}$$

where m_{gel} is the initial mass of gel after equilibration in the osmolyte and m_{dry} is the mass of gel after drying. $q_p\ (PVA - PAAm) = 2.61$. $\emptyset_2$ was calculated from q_p as follows:

$$\emptyset_2 = \left[1 + \frac{(q_p - 1)\rho}{d}\right]^{-1} \tag{4}$$

where *ρ* is the polymer density (1.35 g/ml-PAAm, and 1.29 g/ml- PVA)[8] and *d* is the density of water (1 g/ml). $\emptyset_2\ (PVA - PAAm) = 0.34$.

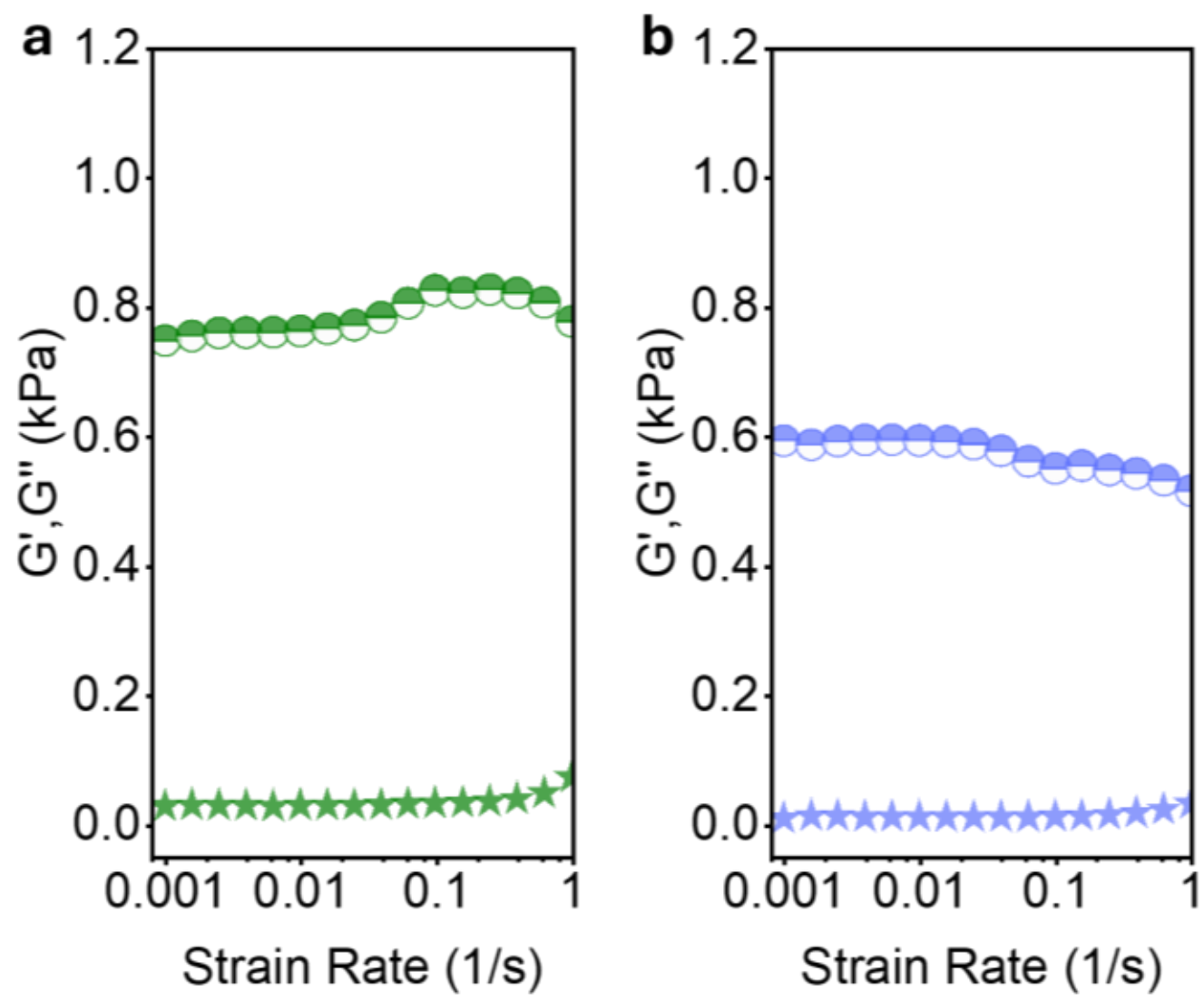

Fig. S3: *G'/G''* vs. strain rate plot of at angular frequency of 10 rad/s for a) PVA-PAAm gel soaked in pure EG; and b) PVA-PAAm gel soaked in PBS. Circle: *G'*, Star: *G''.*

The elastic contribution in the hydrogel system using $\emptyset_2$ was calculated as follows:

$$\pi_{el} = -G = G_{dry}{\emptyset_2}^{1/3} \tag{5}$$

where G_{dry} is the dry gel shear modulus unequilibrated in any solution. G for both pure EG and PBS hydrogel variants were measured through an amplitude sweep experiment conducted at an angular frequency of 10 rad/s using a rheometer (TA instruments HR 30). G ranged ~ 0.8 KPa and ~0.6 KPa for PVA-PAAm/EG and PVA-PAAm/PBS, respectively (Fig. S3)

The contribution from the polymer-solvent mixing interactions was calculated as follows:

$$\pi_{mix} = \frac{-RT}{V_1}[\ln(1 - \emptyset_2) + \emptyset_2 + \chi\emptyset_2^2] \tag{6}$$

where χ is the Flory-Huggins polymer-solvent interaction parameter. For hydrogels, $\chi = 0.48$.[9]

The contributions from the ionic interactions were neglected as the hydrogels were not treated with any ionic solvent during the test.

The contributions from the ionic interactions are calculated as follows:

$$\pi_{ion} = 2RT\{[(C^{sol})^2 + (i\phi_2/(2V_m))^2]^{0.5} - C^{sol}\} \quad (7)$$

where R is the gas constant, T is the absolute temperature, i is the degree of ionization, V_m is the molar volume of the monomer unit of the polymer (13.50 mol/m^3) and C^{sol} is the concentration of the electrolyte with which the gel is treated.[3] For DI, we assumed π_{ion}=0. For 1X PBS, we assumed $C^{sol} = 0.1 \frac{mol}{L}$ and $i = 2$ (since NaCl is the dominant salt). Based on this, $\pi_{tot} = 2.79\ MPa$ and $58\ kPa$ were estimated for PVA PAAm (pure EG) and PVA PAAm (PBS), respectively.

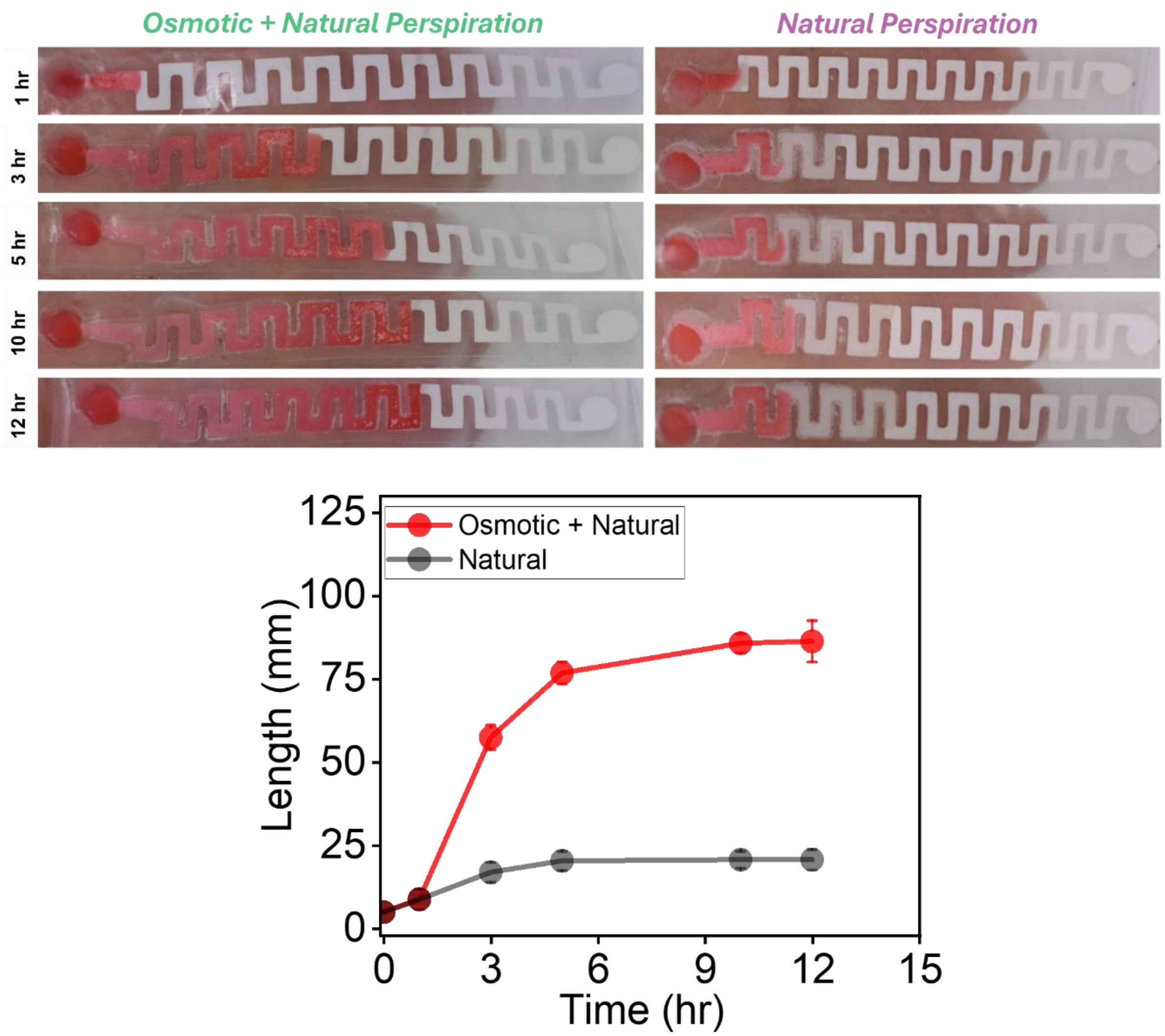

Fig. S4: Optical image of the fluidic channel showing greater dye penetration due to osmosis vs. natural perspiration. Experiments were conducted at the ring finger site. Each

dots denotes average value and error bars denote standard deviation (SD) from *n=3* measurements.

Supplementary Note 2: Von Mises stress estimation on hydrogel surface

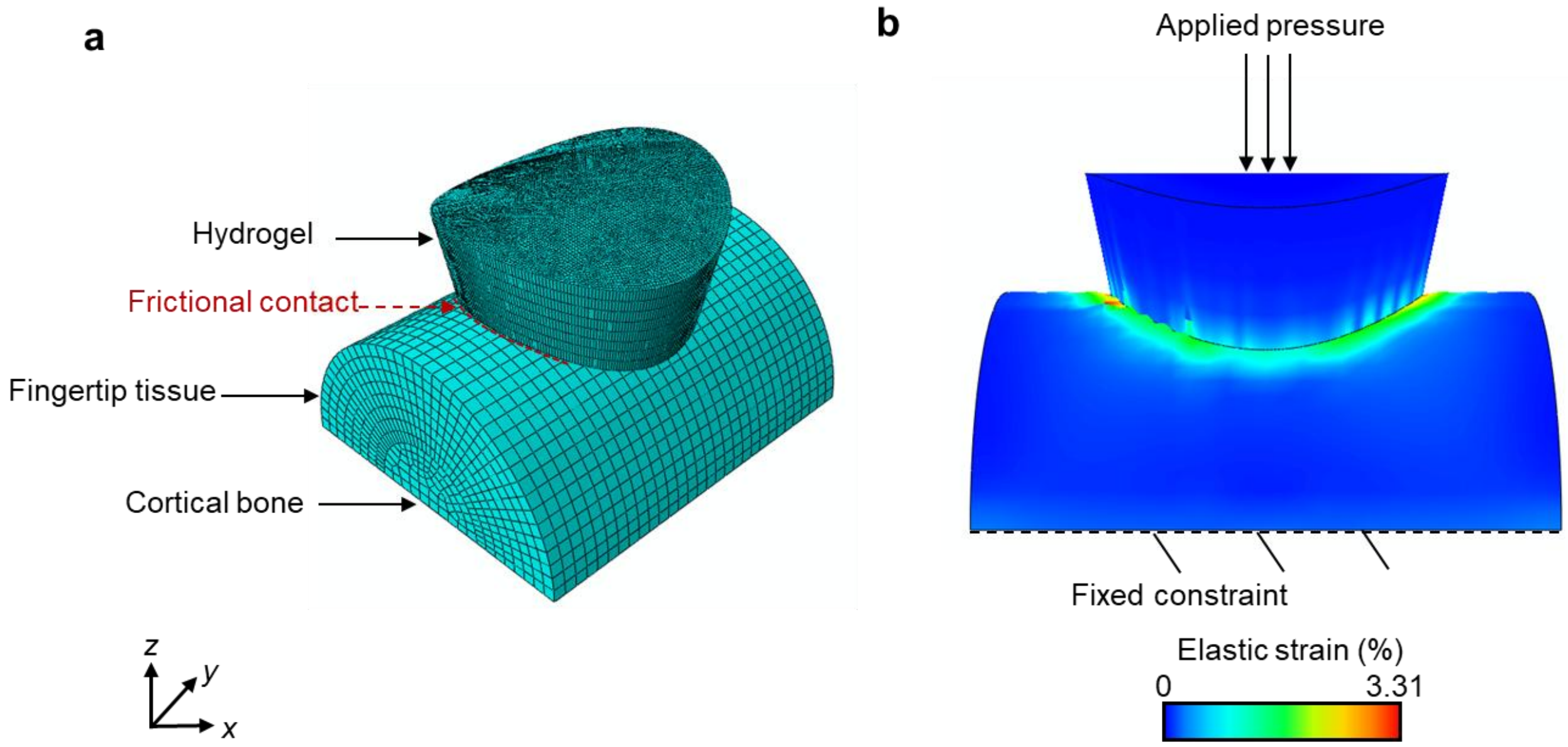

Fig. S5: Mechanical simulation of the hydrogel under bending. a) Isometric view of the assembled meshed system with defined frictional contact between the hydrogel and fingertip tissue. b) Lateral view of the elastic strain on fingertip tissue after applying 3 KPa pressure on the hydrogel surface.

The commercial software ABAQUS was utilized to predict the stresses acting on the hydrogel and fingertip interface under an applied pressure. An 8-node linear brick, reduced integration (C3D8R) mesh (Fig. S5) and explicit dynamic analysis were adopted to ensure the convergence of the simulations. Frictional contact between the hydrogel interface and fingertip tissue was defined with a frictional coefficient of $\mu = 0.15$.[10] Hyperelastic properties were defined by fitting the experimental stress-strain data of the hydrogel. Here, we chose Neo-Hookean hyperelastic model to calculate the coefficients of the hydrogel $C_{10} = 785.25$ and $D_1 = 0.00137$. Linear elastic properties were used to model the fingertip skin and cortical bone where the elastic modulus E and Poisson's ratio v are E_{Skin} = 400 KPa and $v_{\text{Skin}} = 0.48$; $E_{\text{Cortical bone}}$ = 16.2 GPa and $v_{\text{Cortical bone}} = 0.36$.[11,12] We assumed that human skin could accommodate up to ~30% elastic strain and used this as a conservative upper-bound stretchability when estimating the strain in the skin layer induced by applying 3 kPa normal pressure to the hydrogel surface.

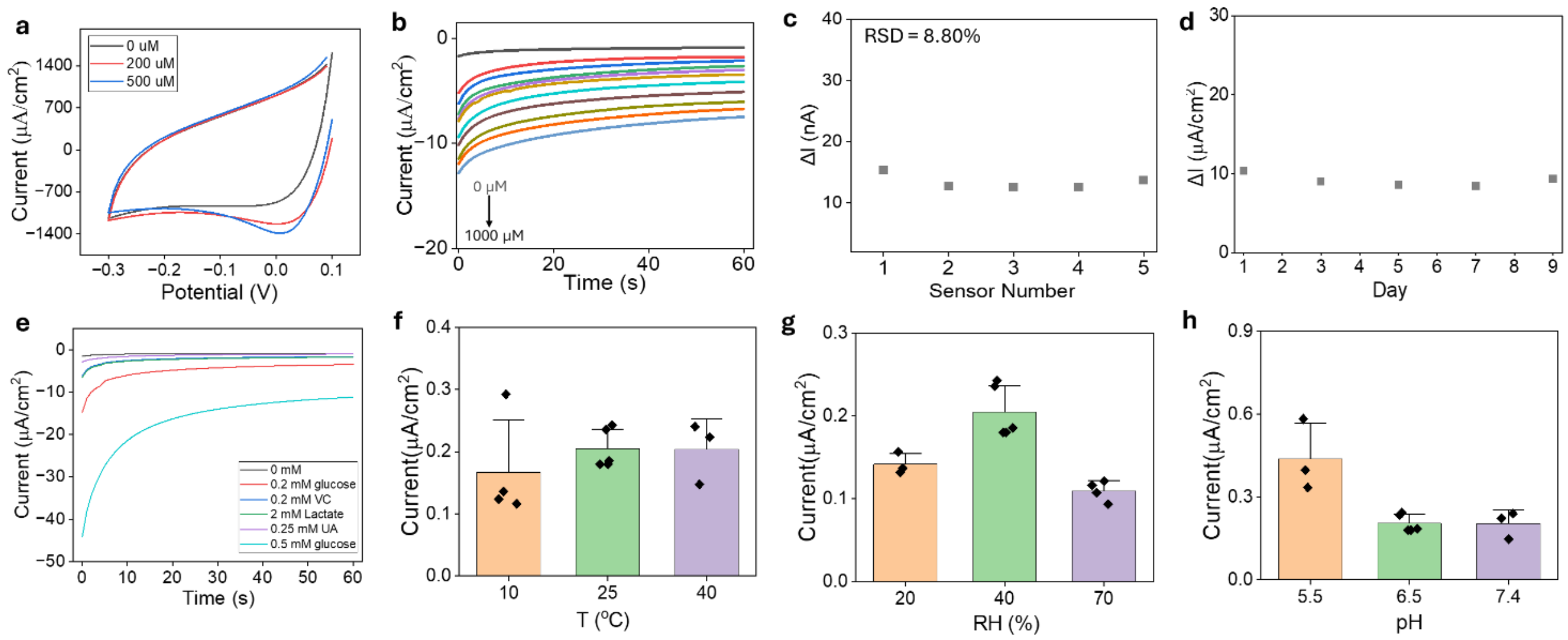

Fig. S6: In-vitro performance of the glucose sensor showing a) cyclic voltammetry (CV); b) chronoamperometric response with 100 μM increments; c) Relative standard deviation (RSD) from 5 sensors; d) Long-term stability of the sensor response; e) interference plot; stability under varying f) temperature, g) relative humidity (RH), and h) pH. Error bars denote standard deviation. Bar denotes mean ± SD from n=4 data points (black dot).

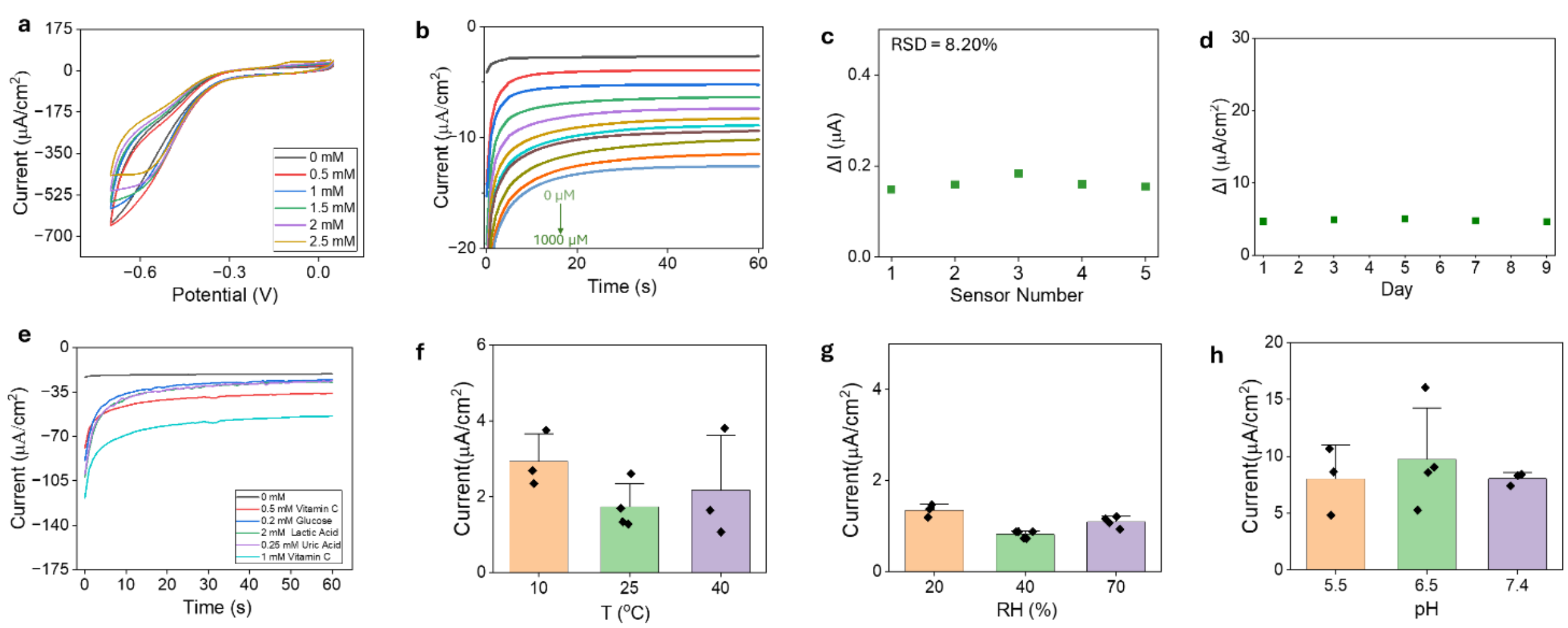

Fig. S7: In-vitro performance of the ascorbic acid sensor showing a) cyclic voltammetry (CV); b) chronoamperometric response with 100 μM increments; c) Relative standard deviation (RSD) from 5 sensors; d) Long-term stability of the sensor response; e) interference plot; stability under varying f) temperature, g) relative humidity (RH), and h) pH. The response follows an inverse trend with pH at a rate of $\sim -0.20\ \frac{\mu A.cm^{-2}}{pH}$. Error bars denote standard deviation. Bar denotes mean ± SD from n=4 data points (black dot).

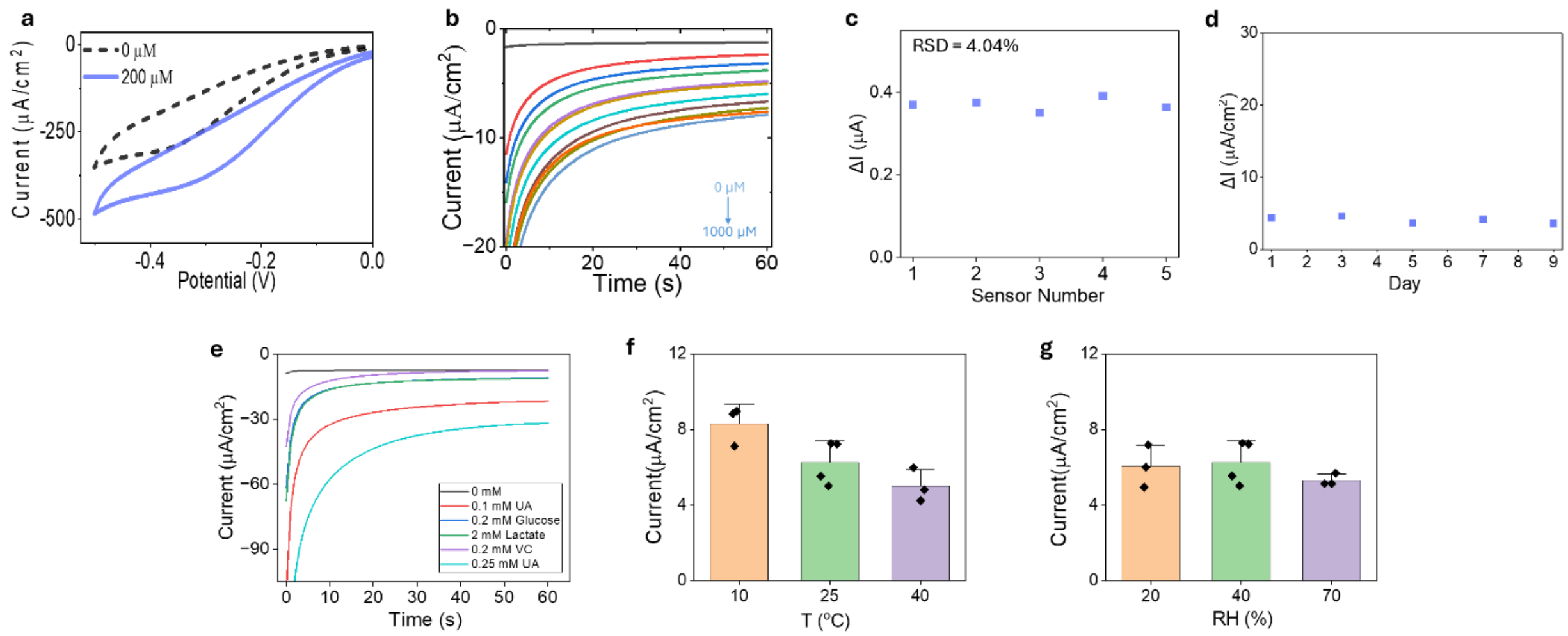

Fig. S8: In-vitro performance of the uric acid sensor showing a) cyclic voltammetry (CV); b) chronoamperometric response with 100 μM increments; c) Relative standard deviation (RSD) from 5 sensors; d) Long-term stability of the sensor response; e) interference plot; stability under varying f) temperature, and g) relative humidity (RH). Error bars denote standard deviation. Bar denotes mean ± SD from n=4 data points (black dot).

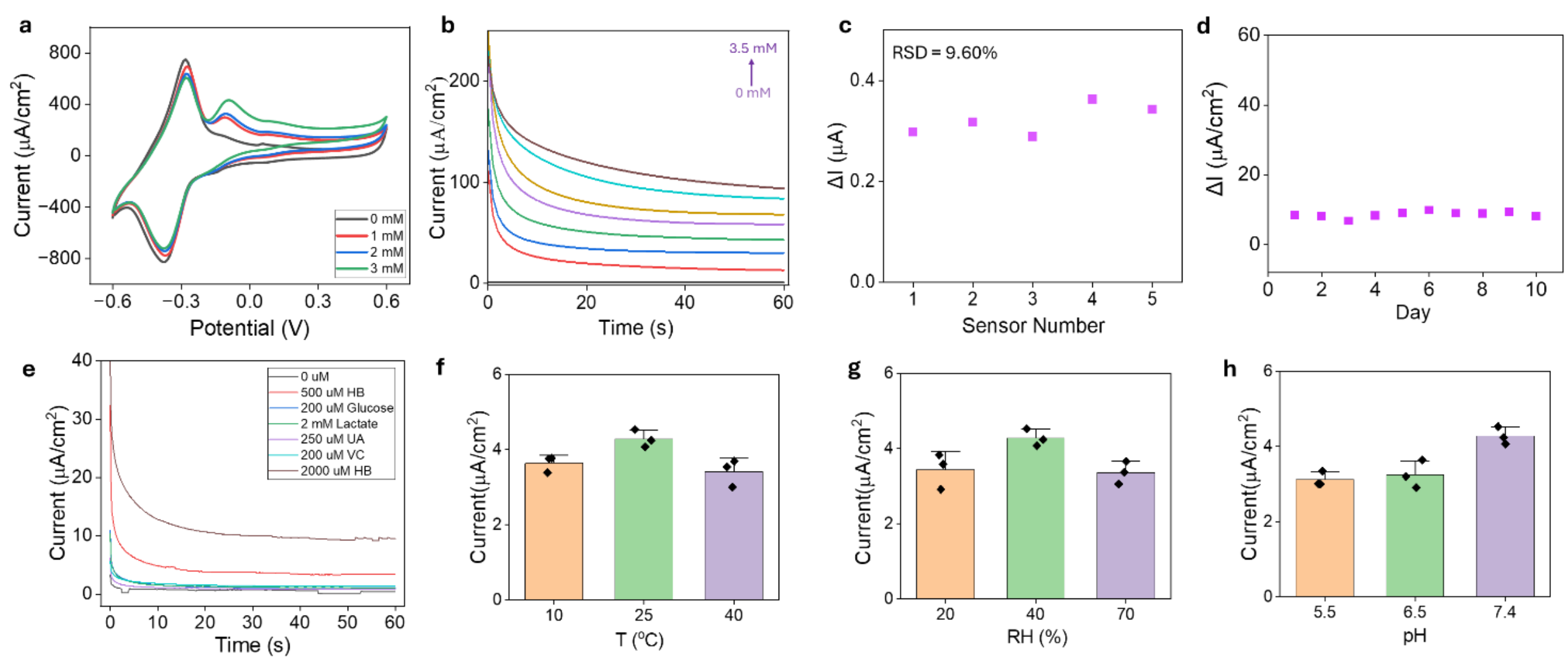

Fig. S9: In-vitro performance of the ketone sensor showing a) cyclic voltammetry (CV); b) chronoamperometric response with 0.5 mM increments; c) Relative standard deviation (RSD) from 5 sensors; d) Long-term stability of the sensor response; e) interference plot; stability under varying f) temperature, g) relative humidity (RH), and h) pH. The response follows an inverse trend with pH at a rate of ~$5.40\ \frac{\mu A.cm^{-2}}{pH}$. Error bars denote standard deviation. Bar denotes mean ± SD from n=4 data points (black dot).

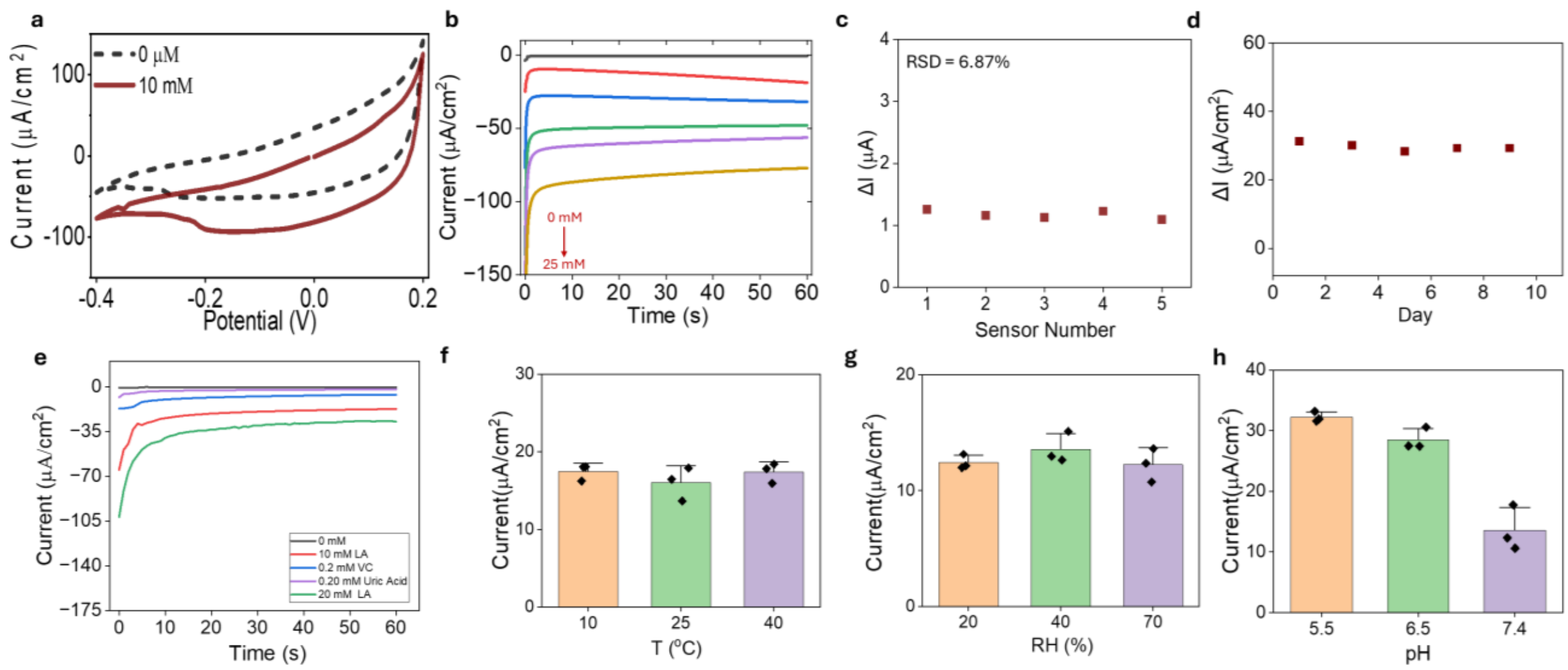

Fig. S10: In-vitro performance of the lactate sensor showing a) cyclic voltammetry (CV); b) chronoamperometric response with 5 mM increments; c) Relative standard deviation (RSD) from 5 sensors; d) Long-term stability of the sensor response; e) interference plot; stability under varying f) temperature, g) relative humidity (RH), and h) pH. The response follows an inverse trend with pH at a rate of ~$-9.10\ \frac{\mu A.cm^{-2}}{pH}$. Error bars denote standard deviation. Bar denotes mean ± SD from n=3 data points (black dot).

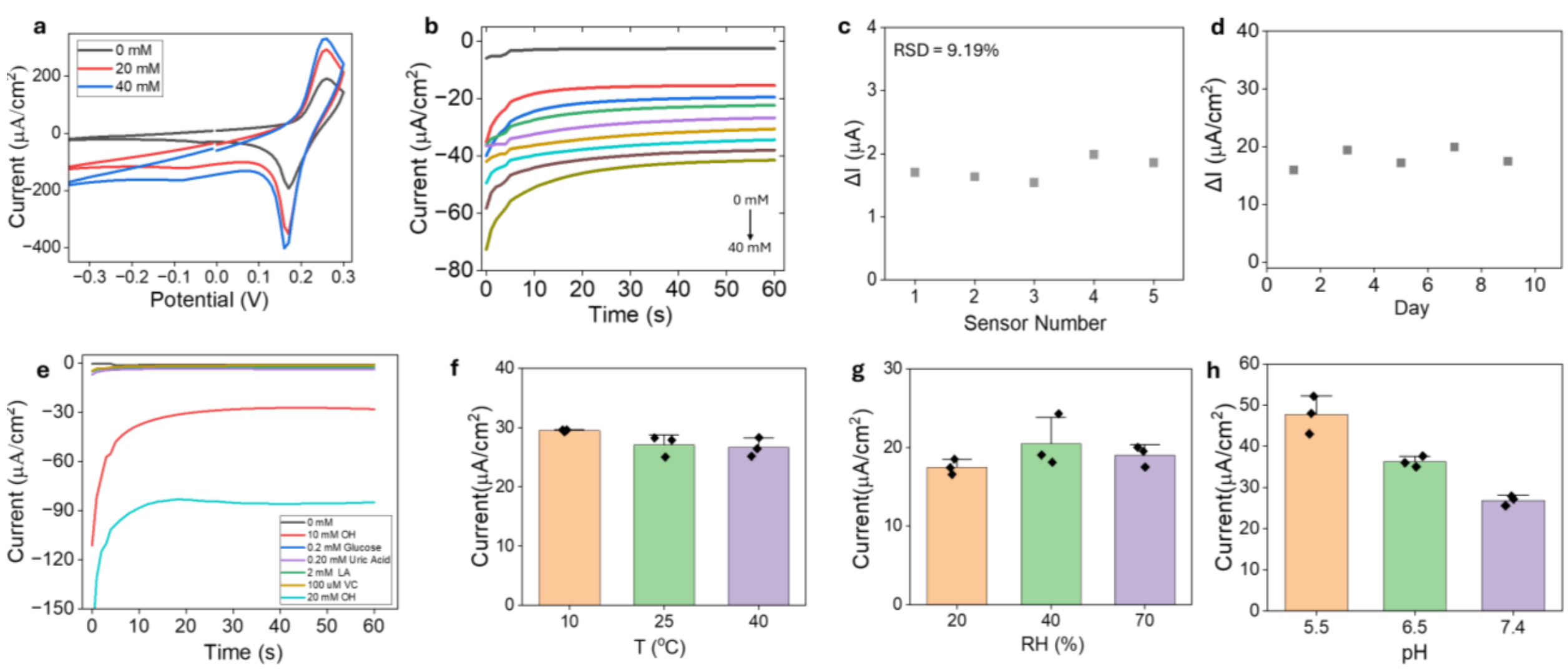

Fig. S11: In-vitro performance of the alcohol sensor showing a) cyclic voltammetry (CV); b) chronoamperometric response with 5mM increments; c) Relative standard deviation (RSD) from 5 sensors; d) Long-term stability of the sensor response; e) interference plot; stability under varying f) temperature, g) relative humidity (RH), and h) pH. The response follows an inverse trend with pH at a rate of ~$-0.77\ \frac{\mu A.cm^{-2}}{pH}$. Error bars denote standard deviation. Bar denotes mean ± SD from n=3 data points (black dot).

Supplementary Note 3: Numerical simulation of analyte flux under varying concentrations and sweat rates

Finite element simulations were performed using COMSOL Multiphysics to model the experimental system in a simplified two-dimensional framework. A fully wetted paper channel operating under incompressible conditions was assumed, and mass conservation was enforced through the continuity equation (Eq. S1). Consistent with the low–Reynolds number regime characteristic of paper-based microfluidic systems ($Re < 1$), the flow was treated as laminar, in agreement with previous reports on lateral flow assays.[13] Under these assumptions, fluid transport within the channel was modeled using the Brinkman-modified Navier–Stokes equations to capture the effects of porosity, permeability, and viscous drag in the porous matrix confined between two parallel plates (Eq. S1). [14]

The channel geometry, along with the initial and boundary conditions applied in the simulations, are shown in Figure S1. Transport of the electroactive analyte was modeled using the convection–diffusion equation (Fick's second law, Eq. S2). The rate constant K was experimentally determined from chronoamperometric measurements conducted at varying analyte concentrations. A linear relationship between the steady-state current I and concentration C was observed (Fig. 2d), validating the first-order reaction assumption. To capture the sensor response under a fixed applied overpotential (i.e., chronoamperometric operation), the Butler–Volmer kinetics were simplified to a first-order reaction with respect to analyte concentration, $J_i = -K_i C_i$, representing the time-dependent consumption of analyte at the electrode surface.[15] The subscript i represents the different tested analytes: glucose, BHB, lactate, UA, UC, and ethanol.

$$\frac{\rho}{\epsilon}\frac{\partial \boldsymbol{u}}{\partial t} = -\nabla P + \mu_{eff}\nabla^2 \boldsymbol{u} - \frac{k}{\mu}\boldsymbol{u},\ \nabla.\boldsymbol{u} = 0 \qquad \text{Eq. S1}$$

$$\frac{\rho}{\epsilon}\frac{\partial c_i}{\partial t} + \boldsymbol{u}.\nabla.c_i = D_{i,eff}\nabla^2 c_i \ \text{ where } J_i = D_{i,eff}\nabla^2 c_i + \boldsymbol{u}c_i \qquad \text{Eq. S2}$$

$$\boldsymbol{u} = (u_{i,x}, u_{i,y}), \qquad J_i = (J_{i,x}, J_{i,y})$$

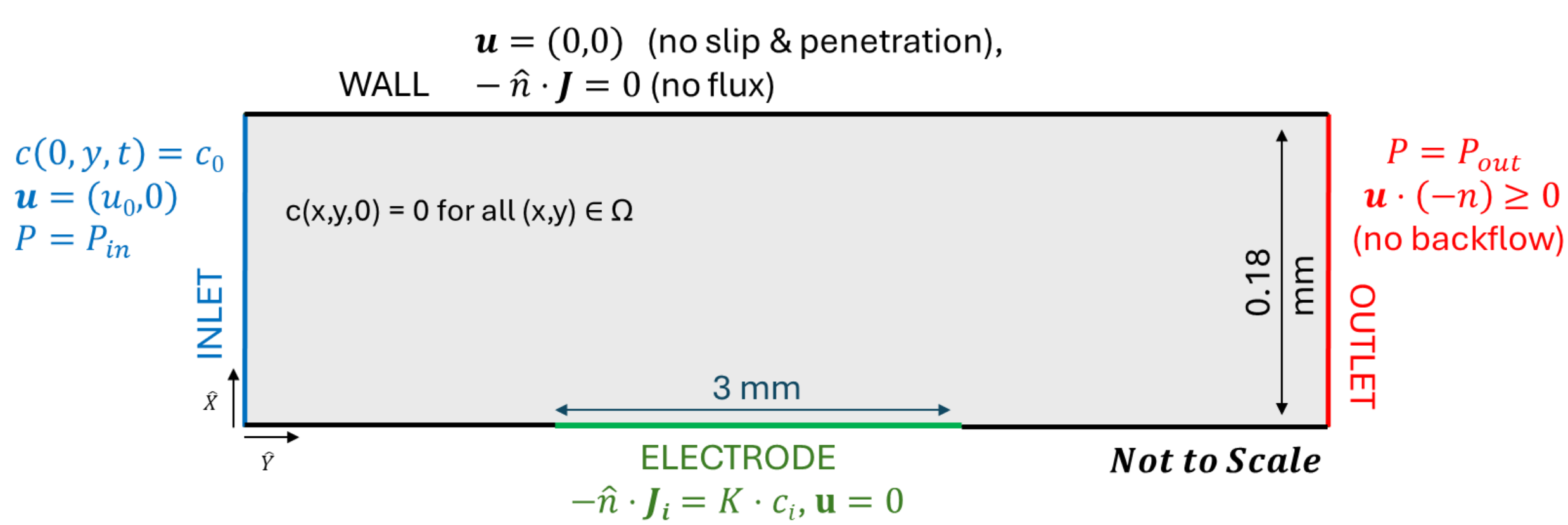

Fig. S12: Schematic illustration and mathematical formulation of the fluid flow numerical model. Equation S1 describes fluid flow using the Navier–Stokes equations with Brinkman corrections, while Equation S2 governs analyte transport via the convection–diffusion equation. The parameters ρ, μ, ε, κ, τ, and D represent the solution density and dynamic viscosity, paper porosity, permeability, tortuosity, and the diffusion coefficient of the analyte, respectively. The effective viscosity and diffusion coefficient are defined as $\mu_{\mathrm{eff}} = \mu/\varepsilon$and $\mathrm{D}_{\mathrm{eff}} = \varepsilon\mathrm{D}/\tau$. The variables u, J, p, c, and $\mathrm{t}$denote the velocity and flux fields, pressure, analyte concentration, and time, respectively; bold symbols indicate vector quantities, and subscripts x and y denote their corresponding components. The subscript i represents the different tested analytes: glucose, BHB, lactate, UA, UC, and ethanol. The parameter K corresponds to the experimentally determined sensor sensitivity. All parameter values used in the simulations are listed in Table S2.

Table S2. Parameters, symbols, and values used in the fluidic numerical simulation.

Parameter	Symbol	Value	Unit
Paper porosity (fraction of void volume)	ϵ	0.7	-
Faraday constant	F	96485.3	$sAmol^{-1}$
Temperature	T	293.15	K
Water dynamic viscosity	μ	0.89	$mPa\ s$
Water density	ρ	997.0	$kg.m^{-3}$
Paper permeability	k	$10e^{-12}$	m^2
Glucose diffusion coefficient	D_{Glu}	$7.1e^{-10}$	$m^2.s^{-1}$
BHB diffusion coefficient	D_{BHB}	$6.6\ e^{-10}$	$m^2.s^{-1}$
Lactate diffusion coefficient	D_{Lac}	$1.1e^{-9}$	$m^2.s^{-1}$
Uric acid diffusion coefficient	D_{UA}	$6.7e^{-10}$	$m^2.s^{-1}$
Vitamin C diffusion coefficient	D_{VC}	$6.4e^{-10}$	$m^2.s^{-1}$
Ethanol diffusion coefficient	D_{Eth}	$1.2e^{-9}$	$m^2.s^{-1}$
Glucose reaction rate constant	K_{Glu}	$4.5e^{-7}$	$m^3A.mol^{-1}$
BHB reaction rate constant	K_{BHB}	$1.7e^{-6}$	$m^3A.mol^{-1}$
Lactate reaction rate constant	K_{Lac}	$1.2e^{-7}$	$m^3A.mol^{-1}$

Uric acid reaction rate constant	K_{UA}	1.4e^{-6}	$m^3A.mol^{-1}$
Vitamin C reaction rate constant	K_{VC}	7.3e^{-7}	$m^3A.mol^{-1}$
Ethanol reaction rate constant	K_{Eth}	2.9e^{-8}	$m^3A.mol^{-1}$

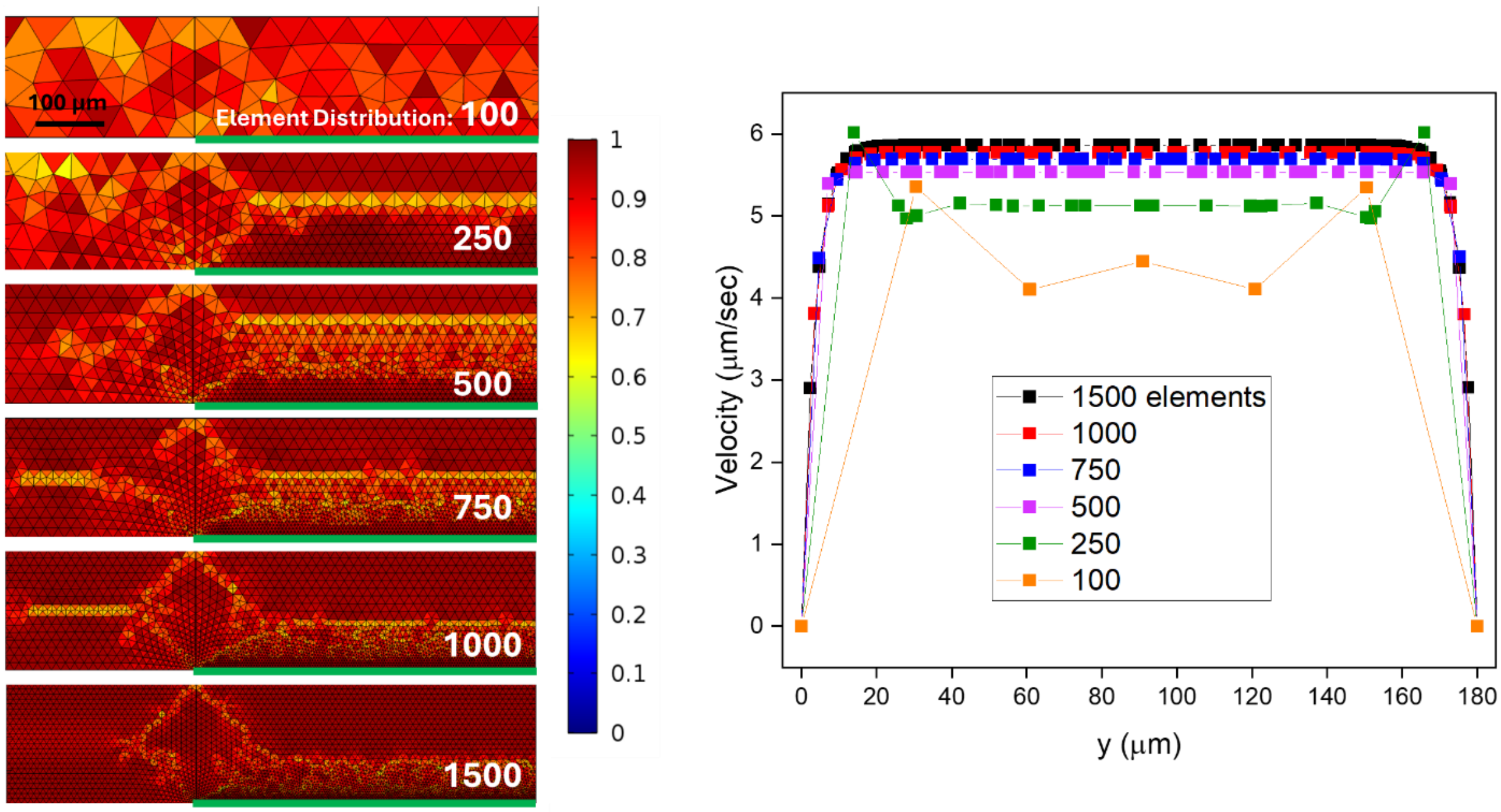

Fig. S13: Mesh Convergence and Numerical Validation. Mesh convergence was verified by progressively refining the mesh and evaluating the channel velocity profile across the channel. Mesh level 5 was selected, with 1,000 elements distributed along the sensor line, as further refinement produced less than a 1% change in velocity. The final mesh consisted of approximately 60,000 elements and required a solving time of under 10 minutes. The resulting velocity profile exhibited plug-like flow, consistent with porous paper channels.

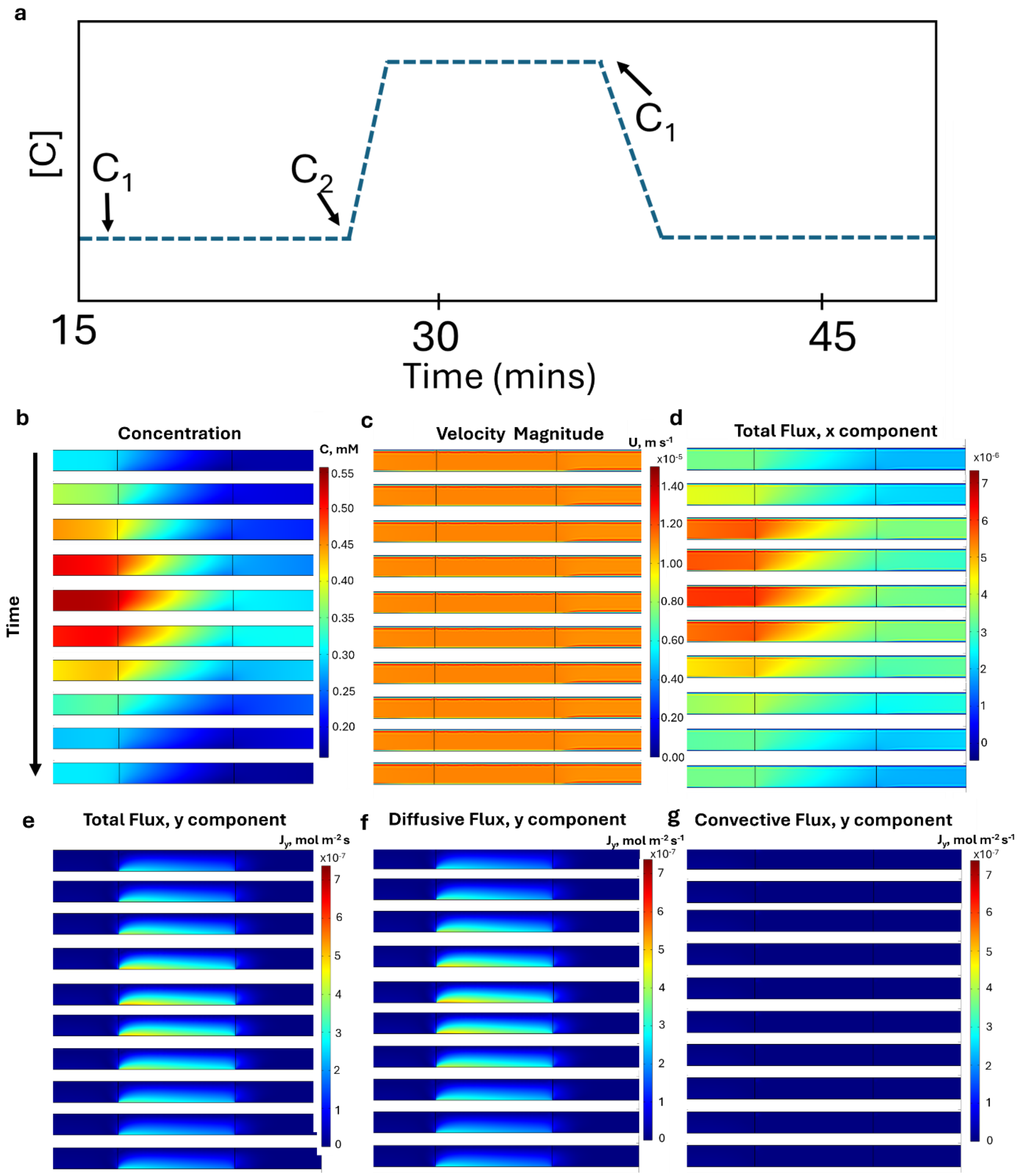

Fig. S14: (a) Schematic showing the input concentration profile used to derive flux data in **Fig. 2g**. $C_1<C_2$. Transient numerical simulation showing time-dependent inlet concentration changes. The inlet analyte concentration was ramped from 0.30 to 0.55 mM and then ramped down to 0.30 mM, each over 200 s, with a 600 s hold period between ramps. The flow velocity was kept constant throughout the entire simulation. The following parameters were monitored and plotted: (b) analyte concentration, showing the

increase and decrease in spatial concentration distribution in response to changes at the inlet; (c) absolute velocity, which remained constant as expected; (d) total analyte flux in the x direction, increasing with inlet concentration primarily due to convection along the channel; (e) total analyte flux in the y direction, exhibiting significant flux along the sensor line and negligible flux elsewhere; (f) diffusive component of the analyte flux in the y direction, matching the magnitude and spatial distribution of the total y-direction flux; and (g) convective component of the analyte flux in the y direction, showing no contribution, as expected. Time stamps: 0,10,13,16,19,22,25,28,31,40 mins.

Supplementary Note 4. Electronic system overview and control architecture

The wearable sensing system consists of a low-power Bluetooth-enabled microcontroller unit (MCU; nRF52840) and an analog front-end (AFE; AD5940). The firmware implements a deterministic finite-state control architecture that minimizes average power consumption while maintaining reproducible electrochemical measurements.

The control program operates cyclically and comprises three primary modes: (i) chronoamperometric data acquisition, (ii) buffered wireless data transmission, and (iii) a low-power sleep interval. During each cycle, electrochemical data are acquired and stored locally in memory, transmitted wirelessly in a short burst after acquisition is complete, and followed by a prolonged low-power idle period before the next acquisition cycle begins.

1.1 Chronoamperometric acquisition protocol

Chronoamperometric (CA) measurements are performed sequentially across four electrodes using a software-controlled switch matrix. For each electrode configuration, data rate, excitation voltage, and sampling period are all individually adjustable.

The four electrode configurations are measured consecutively in a user-defined order. The AFE generates measurement-ready events that are detected by the MCU, and the MCU reads and stores the corresponding current values. The AFE remains active only during the acquisition phase and is explicitly shut down after completing the fourth channel to reduce static power consumption.

1.2 Firmware state machine and timing control

The firmware is implemented as an explicit state machine with four main states: initialization, acquisition, transmission, and sleep. After system initialization, the firmware enters the acquisition state, during which chronoamperometric measurements are performed sequentially across the four electrode configurations. A sample counter tracks the number of measurements acquired per channel and triggers a state transition once the predefined number of samples is reached.

Upon completion of data acquisition, the firmware transitions to the transmission state, during which the BLE radio is enabled, and the locally buffered data is transmitted to an external receiver in bursts. After successfully transmitting all stored samples, the firmware enters a low-power sleep state for a predefined interval before restarting the cycle.

1.3 Data buffering and wireless transmission

All electrochemical data are stored locally in the MCU's memory during the acquisition phase. Data is organized sequentially by channel and time and is not transmitted in real time. This buffering strategy enables the BLE radio to remain disabled during sensing, thereby reducing radio duty cycle and minimizing interference between wireless communication and electrochemical measurements.

During the transmission state, buffered data are packetized and transmitted via BLE notifications. Each packet includes metadata indicating the electrode configuration and sample index to ensure correct reconstruction of the time series at the receiver. After all buffered data is transmitted, the BLE stack is explicitly de-initialized to prevent background radio activity during subsequent low-power operation.

1.4 Low-power operation and duty cycling strategy

To minimize average power consumption, the firmware employs aggressive duty cycling of high-power subsystems. The BLE radio and associated high-frequency clocks are enabled only during the short transmission window following data acquisition. During the acquisition phase, BLE is disabled, and only the AFE and required MCU peripherals are active.

After data transmission, the AFE is placed into a shutdown state, and all wireless communication interfaces are disabled. The MCU then enters a low-power idle state implemented using a delay-based sleep mechanism, which was empirically found to yield the lowest baseline current consumption on this platform. The sleep interval was set to 6 min, substantially longer than the acquisition and transmission durations, ensuring that the system spent most of its operating cycle in a low-power state while continuously acquiring meaningful CA data.

1.5 Watchdog-based autonomous restart

Rather than preserving MCU state across sleep intervals, the system employs a watchdog-timer-based restart mechanism. A hardware watchdog timer is configured to reset the MCU after the sleep interval elapses. Upon reset, the firmware restarts from a known initial state and initiates a new acquisition cycle.

This approach eliminates the need to retain RAM or peripheral state during sleep, reducing idle-state power consumption, and ensures robust long-term operation without

cumulative state drift. Using a full system reset also simplifies the firmware design and improves fault tolerance.

Supplementary Note 5. Debug and deployment configurations

Two firmware configurations are used during development and testing. In a debug configuration, USB serial communication is enabled to allow real-time logging and diagnostic output. In a deployment (low-power) configuration, all USB-related functionality is disabled to reduce baseline current consumption. The functional behavior of the control program is otherwise identical between configurations. The circuit is programmed using a custom-designed desktop probe station. Firmware flashing is done using an NRF52840 development kit with the NRF Connect desktop software.

Supplementary Note 6. Power consumption measurement methodology 1

Power consumption measurements are performed with the system powered via the custom-designed desktop probe station and a Nordic Power Profiler Kit. Current consumption is displayed and recorded in real time using the power profiler tool GUI on a PC, and characteristic current levels are recorded for the acquisition, transmission, and sleep phases.

Distinct current plateaus corresponding to the acquisition phase, the BLE transmission burst, and the low-power sleep interval are observed, confirming the correct operation of the duty-cycled control program. Disabling USB and BLE during sleep results in a substantial reduction in baseline current compared to debug configurations.

Supplementary Note 7. Power consumption measurement methodology 2

The received signal strength indicator (RSSI) of the BLE communication is characterized using a smartphone-based application (nRF Toolbox, Nordic Semiconductor). During these measurements, the circuit operates in its standard transmission mode, in which buffered data are transmitted via BLE notifications after the acquisition phase completes.

RSSI measurements are performed in a laboratory environment with a maximum separation distance of 3 m between the circuit and the smartphone. The smartphone receiver is held stationary at each measurement location to minimize motion-induced variability. RSSI values are recorded over multiple transmission cycles to evaluate the stability and consistency of the wireless link under typical operating conditions. These measurements are intended to provide a practical assessment of communication range and link quality rather than a calibrated radio-frequency characterization.

Supplementary Note 8. Power consumption measurement methodology 3

Thermal imaging is performed to assess temperature changes of the wearable circuit during a complete operating cycle. Thermal images are acquired with an infrared thermal

camera (FLIR C5) at 0 s, 60 s, 300 s, and 600 s after the start of a measurement cycle. These points span the full sequence of electrochemical acquisition, wireless data transmission, and low-power sleep.

Over the 10 min operating cycle, the device's maximum surface temperature increases from 27.3 °C at the start to 31.0 °C at the end. The observed temperature rise is gradual and stabilizes during the latter portion of the cycle, consistent with the system's duty-cycled operation, in which high-power activities are confined to short acquisition and transmission intervals.

The measured temperature increase remains well within ranges considered safe for prolonged skin-contact wearable electronics, indicating that the device does not produce excessive thermal loading during normal operation.

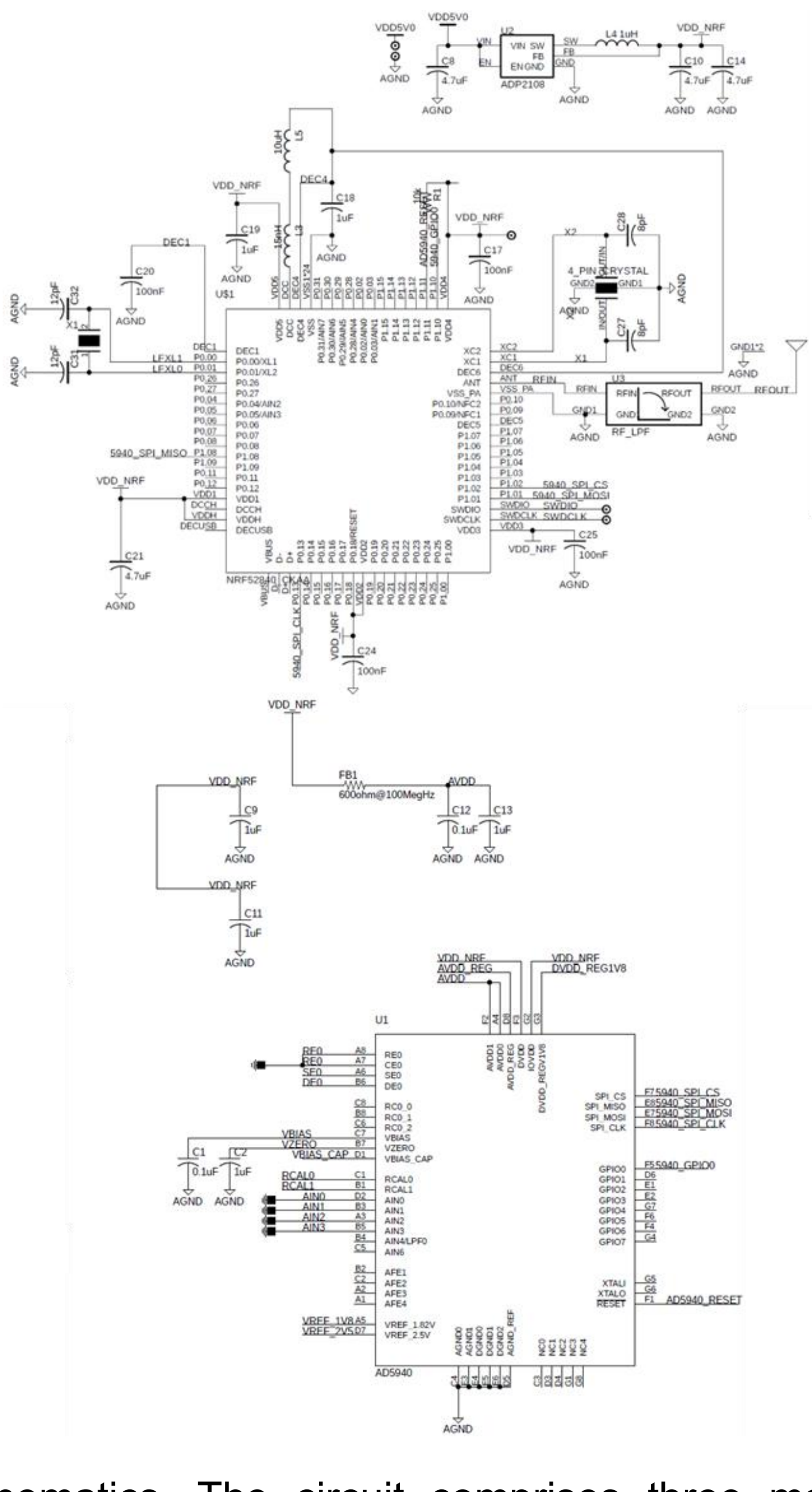

Fig. S15: Circuit schematics. The circuit comprises three major components: the microcontroller unit (MCU, NRF52840, Nordic Semiconductor), the analog front end

(AFE, AD5940, Analog Devices), and a power converter (ADP2108, Analog Devices). All components are selected to minimize board area while maintaining high sensitivity, low noise, and low power consumption.

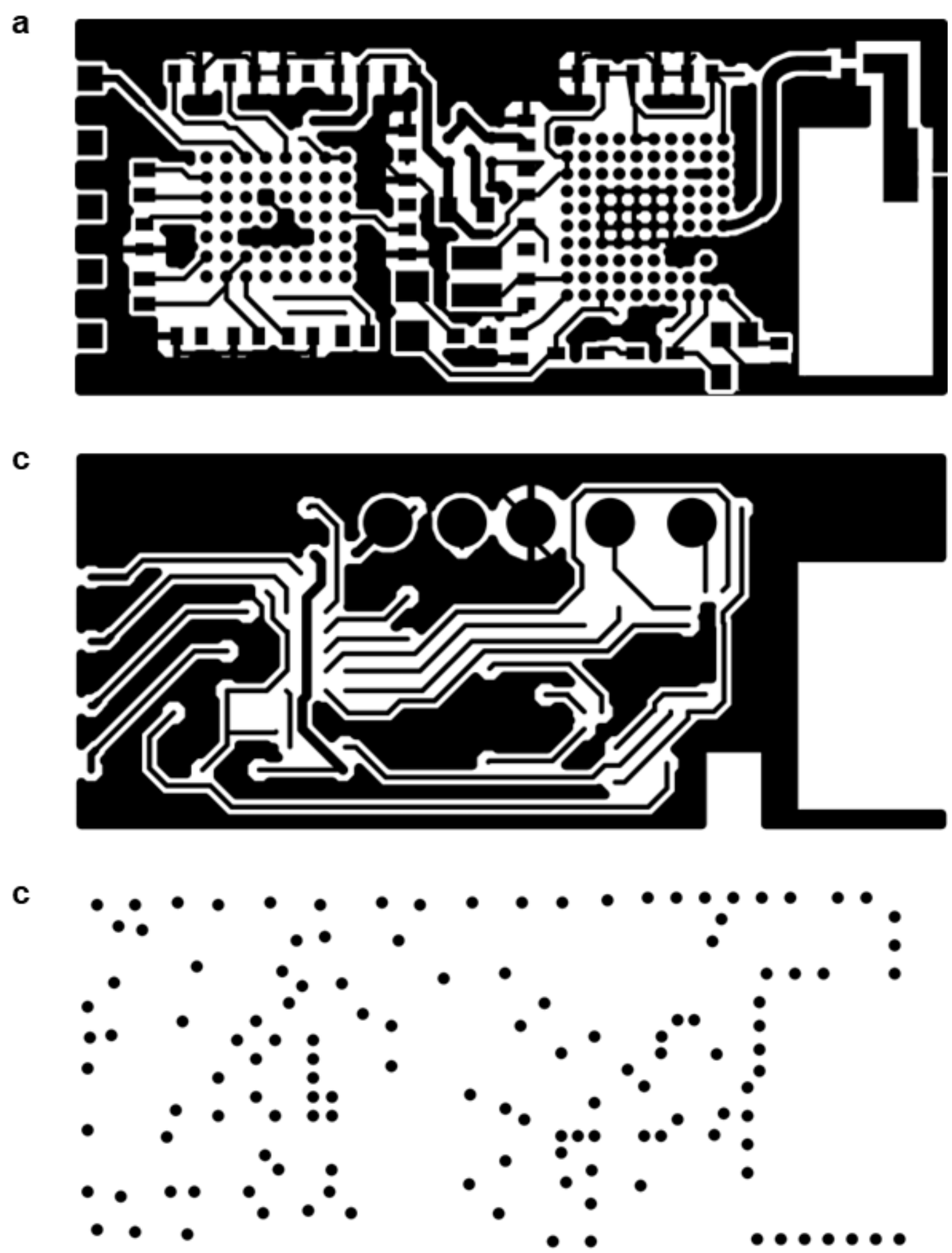

Fig. S16: F-PCB layer and drill layout. a. fPCB top layer copper pattern, large areas of copper fill are applied to simplify routing in a compact area while maintaining good noise suppression and RF transmittivity; b. fPCB bottom layer copper pattern. Thin copper traces (75 µm) are used to fan out the delicate BGA chips properly; c. fPCB drill vertical interconnect access (VIA) drill pattern. Stitched VIAs are applied along the board edge and in the RF antenna region to minimize ingress noise and radiated emissions back into the board.

Qty	Value	Footprint Name	Parts
4	0.47uF	C0201	C3, C4, C6, C7
6	100nF	C0201	C1, C12, C17, C20, C24, C25
1	10uH	IND_0402	L5
2	12pF	C0201	C31, C32
1	15nH	IND_0201	L3
6	1uF	C0201	C2, C9, C11, C13, C18, C19
1	1uH	IND_0302	L4
1	LF Crystal	LF Crystal	X1
5	4.7uF	C0201	C5, C8, C10, C14, C21
1	HF Crystal	HF Crystal	X2
1	600ohm@100MegHz	R0201	FB1
2	8pF	C0201	C27, C28
1	AD5940	AD5940	U1
1	ADP2108	ADP2108	U2
1	ANT_2450AT07A0100	2450AT07A0100	ANT1
1	NRF52840_CKAA	NRF52840	U$1
1	RF_LPF	2450LP07C0100	U3

Fig. S17: Bill of materials. The picture shows the component values, footprint, and parts name used to assemble the circuit. 0201 package is used whenever possible to save the board size.

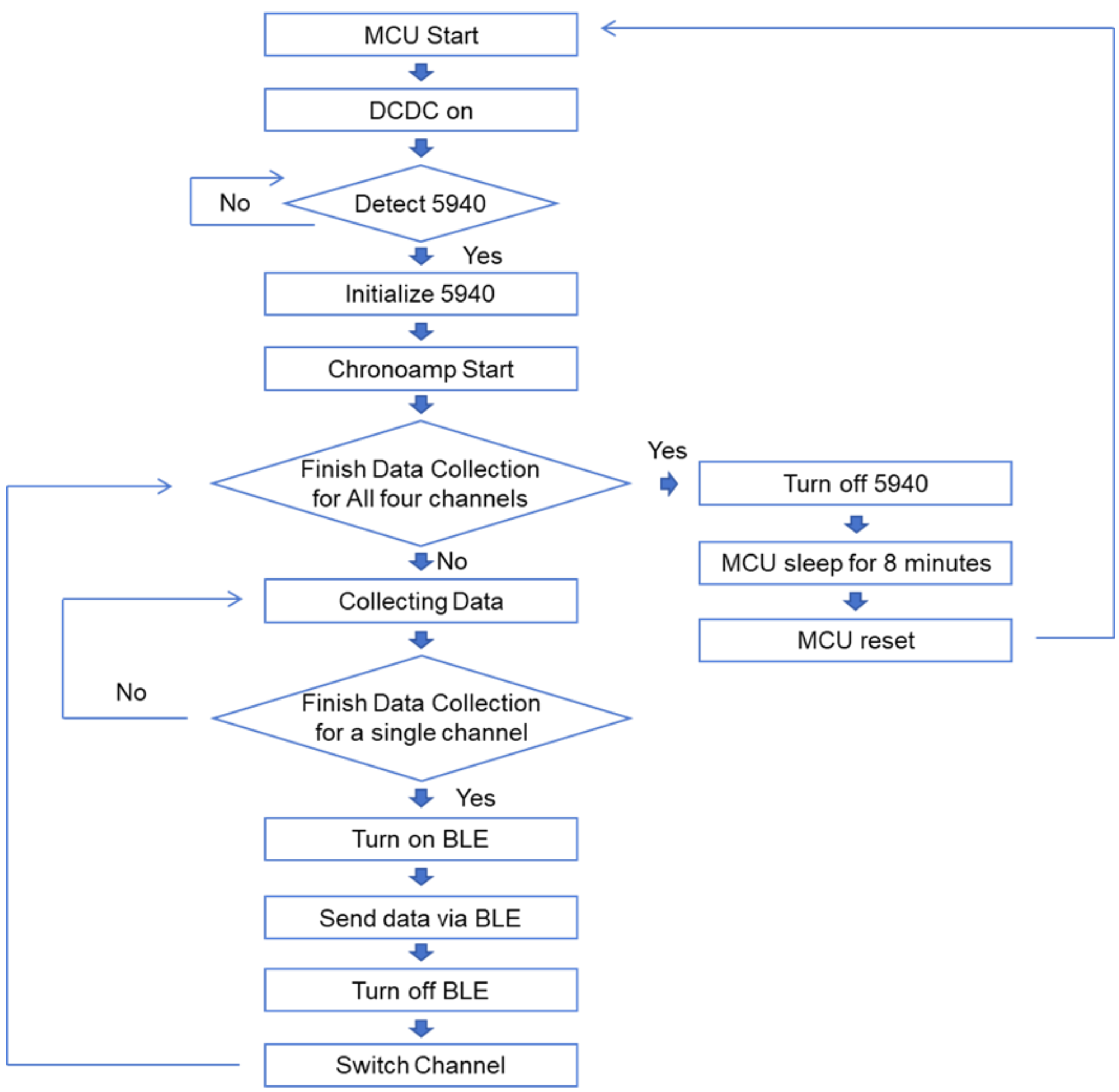

Fig. S18: Low-power control flow chart. The low-power sensing program starts by enabling the MCU's DC-DC converter to reduce MCU power consumption. It then detects

whether the AFE is appropriately connected. If yes, the AFE is initialized, and the chronoamperometry at the first sensor location starts. Then, Bluetooth Low Energy (BLE) is enabled to wirelessly transmit the data to a receiver, after which it is shut down again for power savings. The AFE then switches to the next channel and repeats this three more times before entering the idle state. It then sleeps for 8 minutes before starting a new round of sensing.

Table S3: Effect of supply voltage on chronoamperometric current measurement. A 500 mV bias was applied across a 1.02 MΩ resistor. The measured mean current remained similar from 2.8 to 3.8 V, while the 3.3 V supply produced the lowest standard deviation. Operation at 2.3 V resulted in increased variation, indicating degraded measurement stability outside the nominal operating range.

Supply Voltage (V)	Mean current (μA)	STD (μA)
2.3	0.0477	0.0247
2.8	0.0488	0.0041
3.3	0.0487	0.0036
3.8	0.0495	0.0057

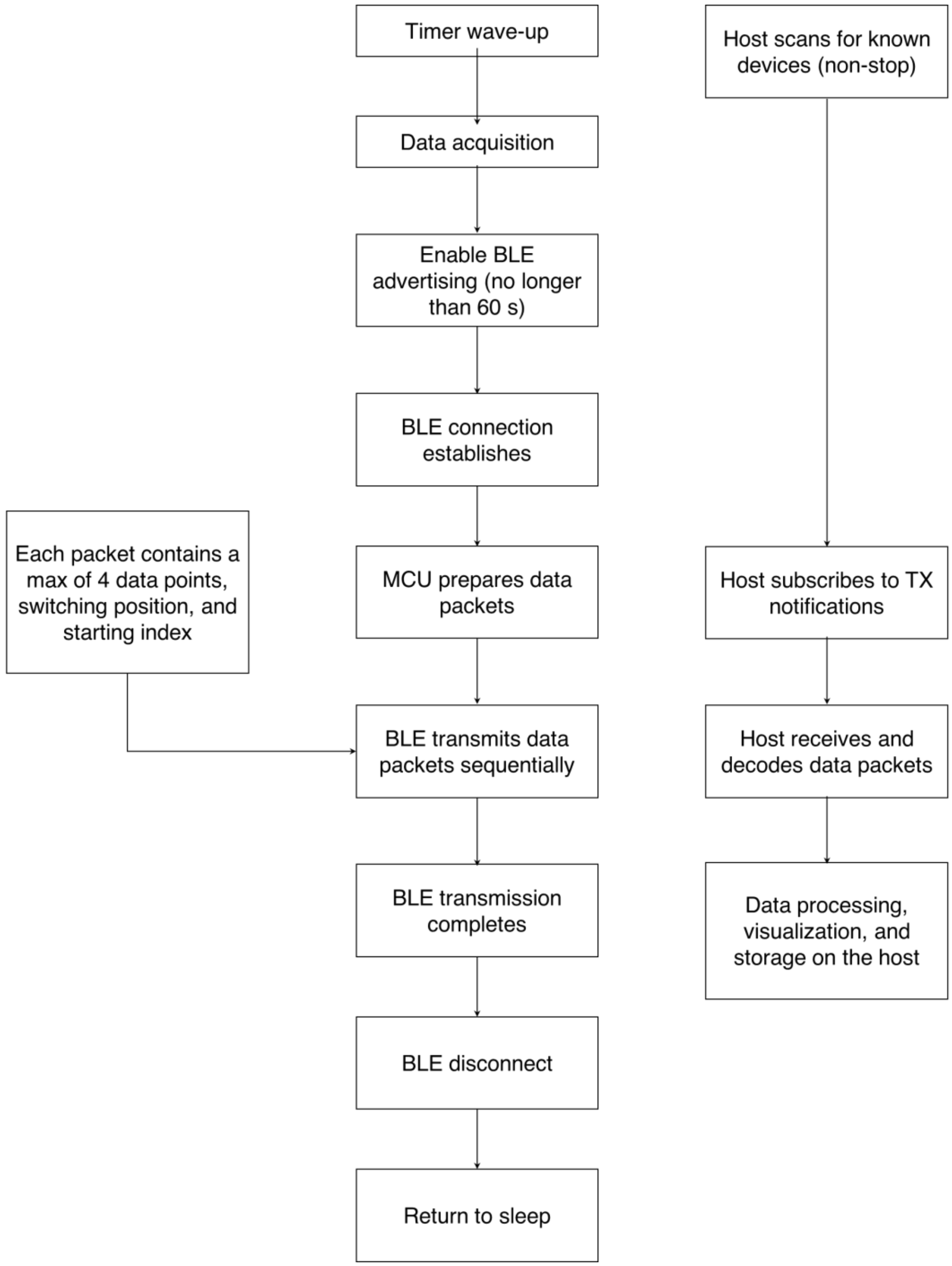

Fig. S19: BLE data acquisition and transmission workflow. The wearable device wakes up periodically by timer, performs data acquisition, enables BLE advertising, establishes a BLE connection, prepares data packets, sequentially transmits the packets, disconnects, and then returns to sleep. Each packet contains up to four data points, including position and starting index information. On the host side, the receiver continuously scans for the known device, subscribes to TX notifications after connection, receives and decodes the transmitted packets, and performs data processing, visualization, and storage.

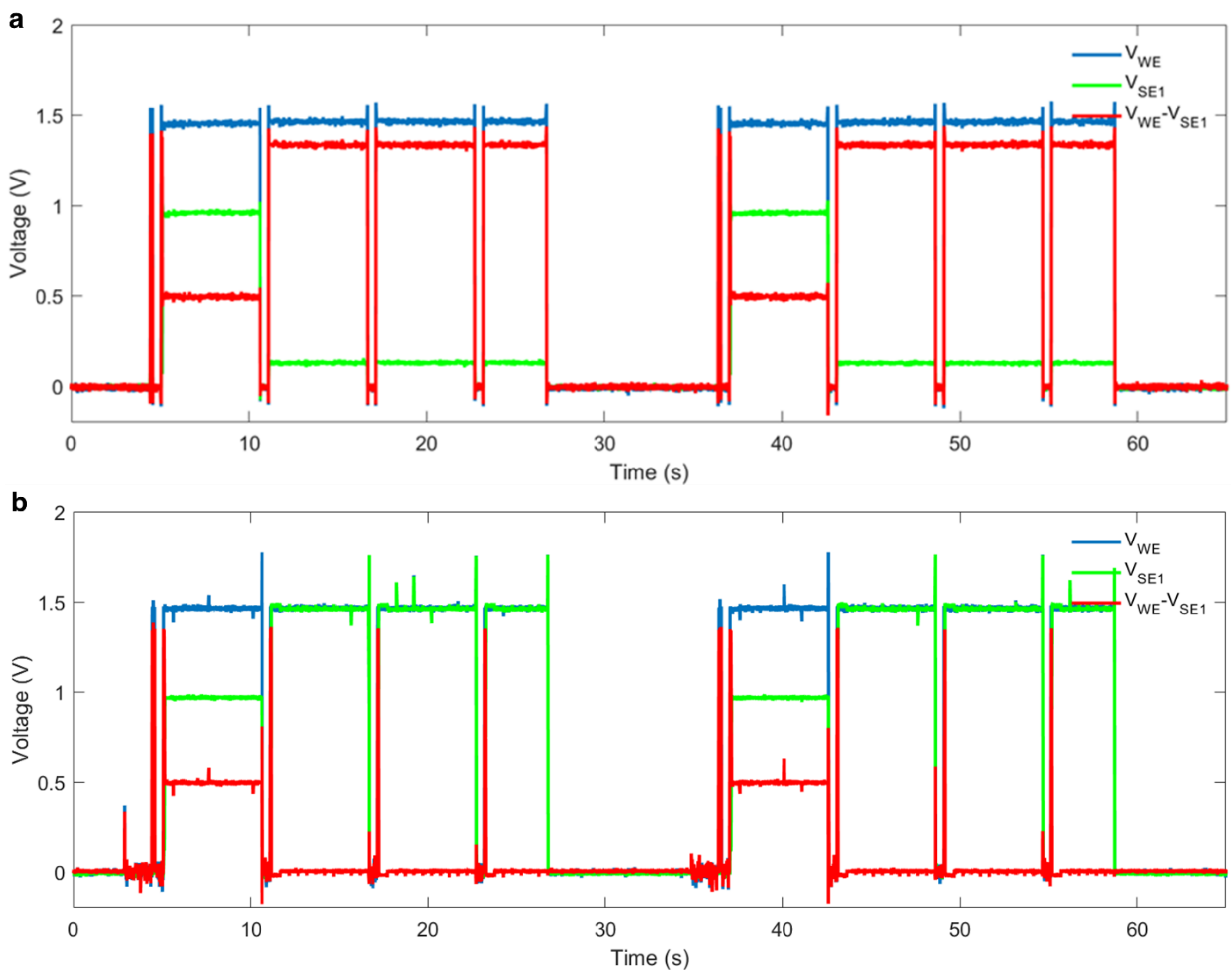

Fig. S20: DC bias resistor function: a. Plot of working electrode voltage (blue), sensing electrode voltage (green), and the voltage across the sensor (red). The plot shows that the voltage across the sensor is not 0 V even when the sensor is not actively being sensed. b. The plot shows the same voltage-recording setup, but with the DC bias resistor included. The plot shows that the voltage across the sensor is 0 V when it is not being sensed, and 500 mV when actively sensing, proving the effectiveness of the DC bias resistor.

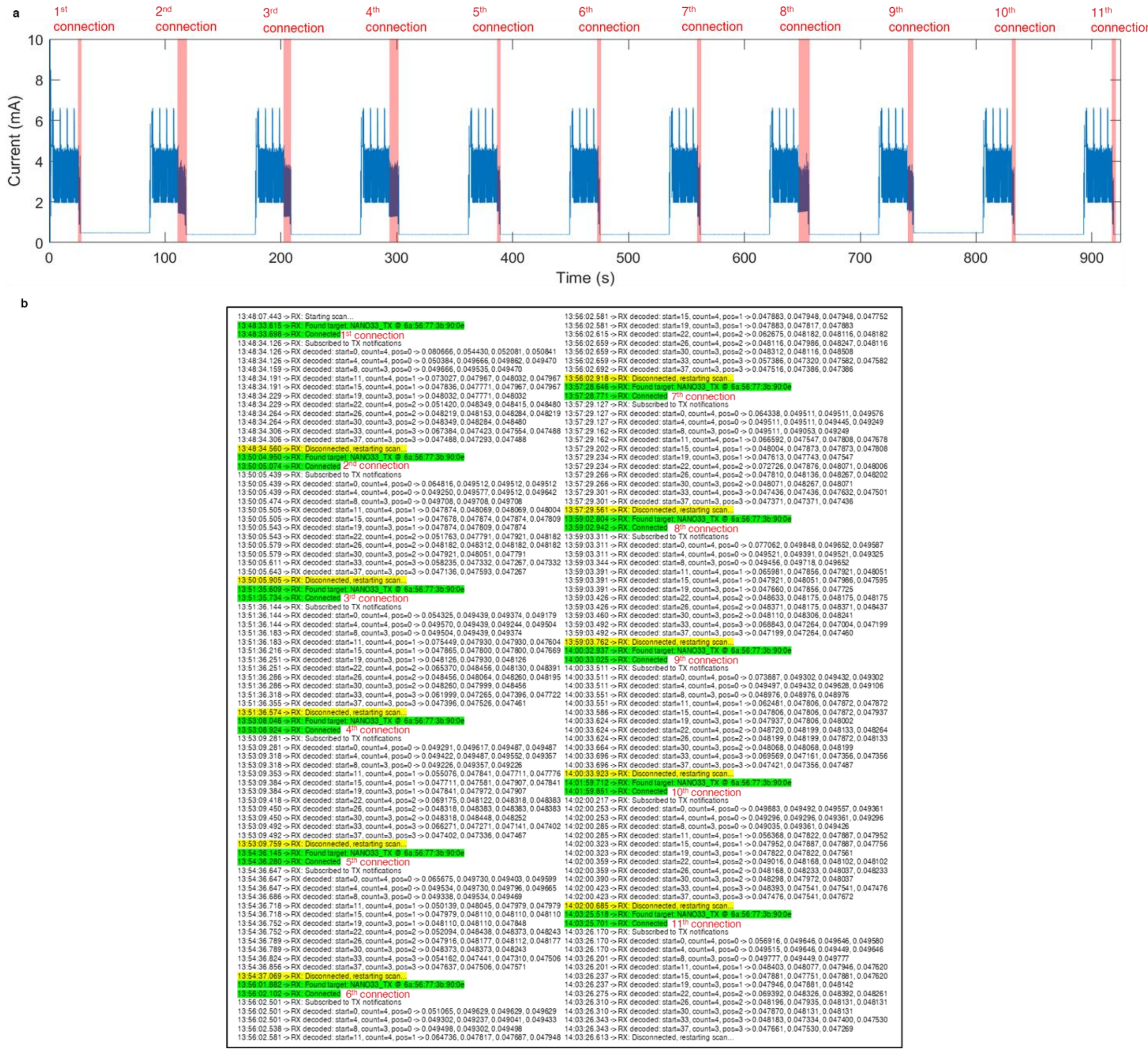

Fig. S21: Reconnection after sleep: a. Power consumption plot of accelerated experiment protocol showing 11 wake-acquisition-transmit-sleep cycles; b. Corresponding original serial monitor outputs from the BLE receiver showing timestamps, BLE events, and data packets demonstrate successful BLE reconnection even after multiple sleep-wake cycles.

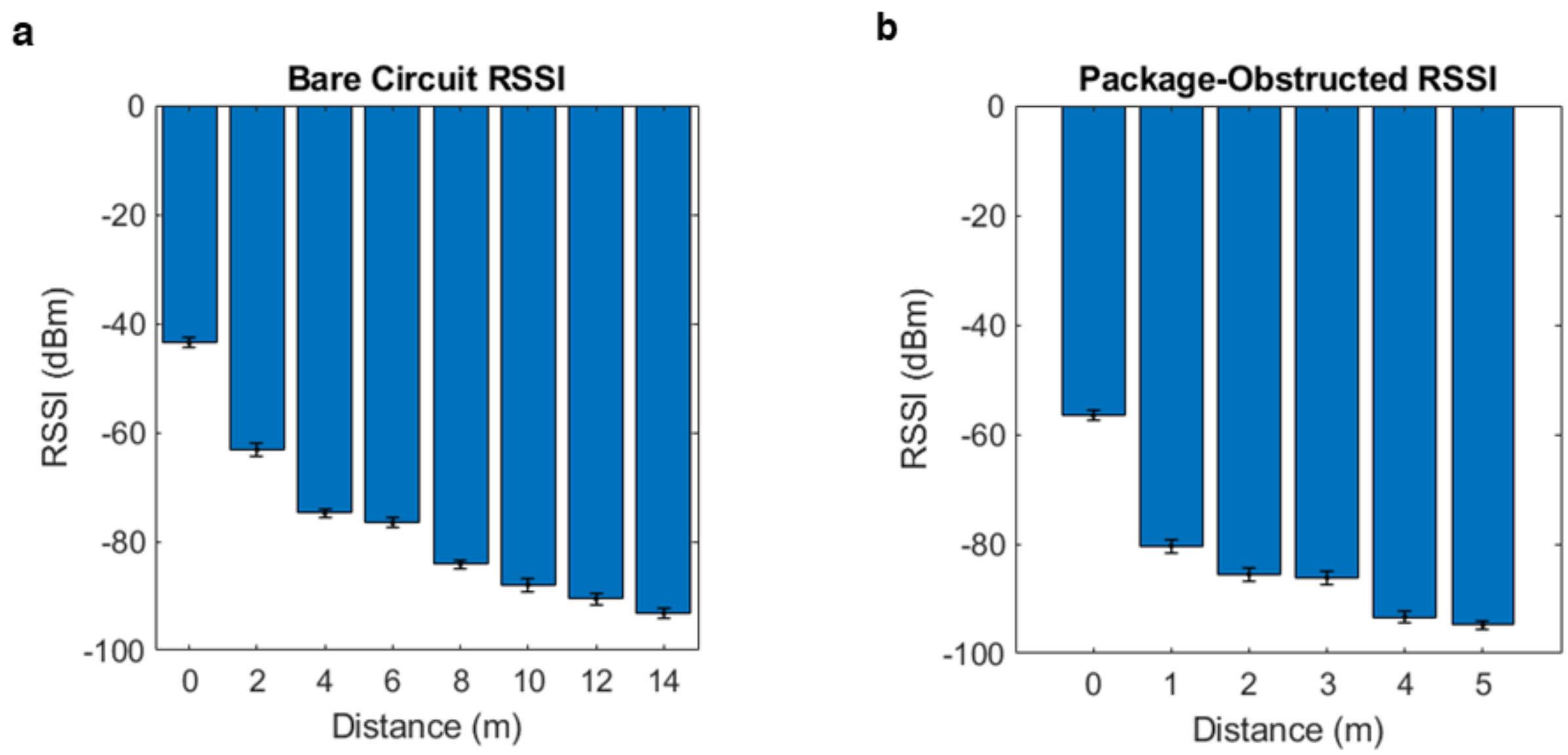

Fig. S22: RSSI measurement under different device configurations: a. RSSI of the bare circuit measured in a bare circuit configuration. The device maintained BLE communication up to approximately 14 m, with RSSI decreasing from approximately −43 dBm at 0 m to approximately −93 dBm at 14 m. b. RSSI of the fully packaged ring was measured in an off-body configuration. The communication range decreased to approximately 5 m, with RSSI reaching approximately −95 dBm. Error bars represent the standard deviation from 5 repeated RSSI measurements.

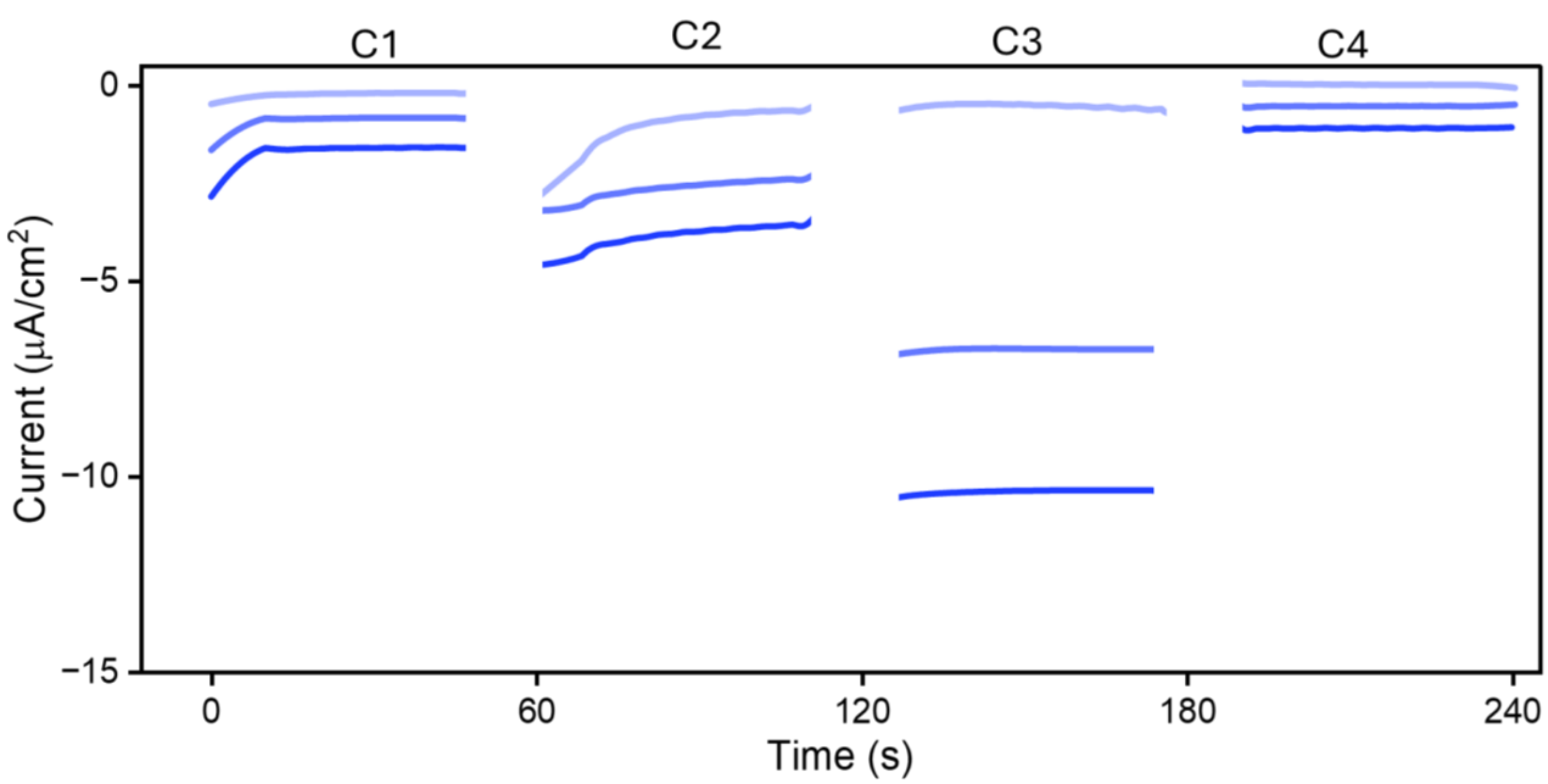

Fig. S23: Chronoamperometric response from 4 channels in the F-PCB, operating sequentially for peroxide detection in a Prussian blue-carbon electrode system. C1:

Channel 1 at -0.05V, C2: Channel 2 at -0.5V, C3: Channel 3 at -0.3V, and C4: Channel 4 at 0.05V. All four working electrodes shared a common reference electrode. Peroxide concentrations spiked: 0,500,1000 μM.

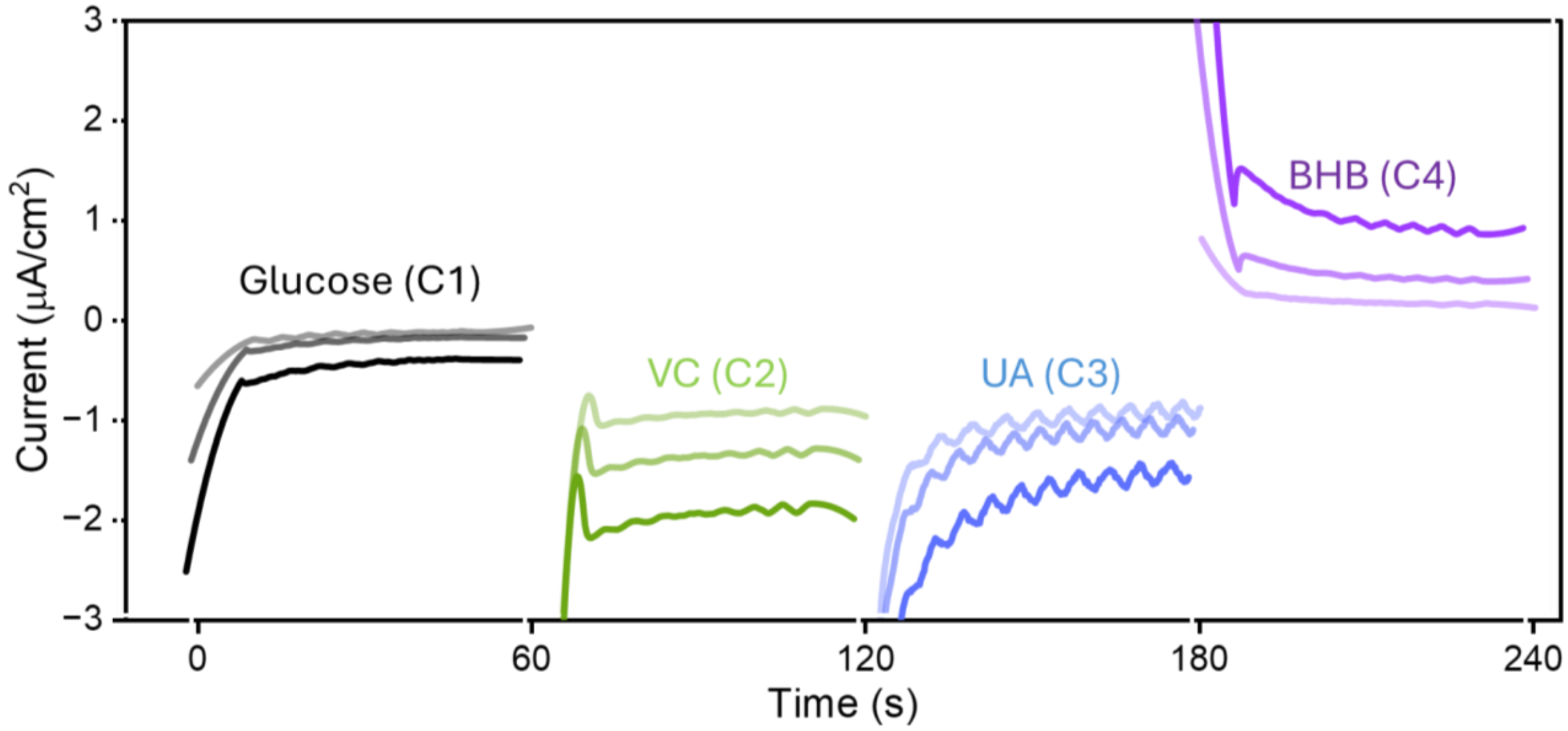

Fig. S24: Chronoamperometric response from 4 channels in the F-PCB, operating sequentially for glucose, VC, UA, and BHB detection at their respective potentials. Glucose concentrations: 0,200,500 μM; VC concentrations: 0,200,400 μM; UA concentrations: 0,100,400 μM; BHB concentrations: 0,400,1000 μM. All four working electrodes shared a common reference electrode.

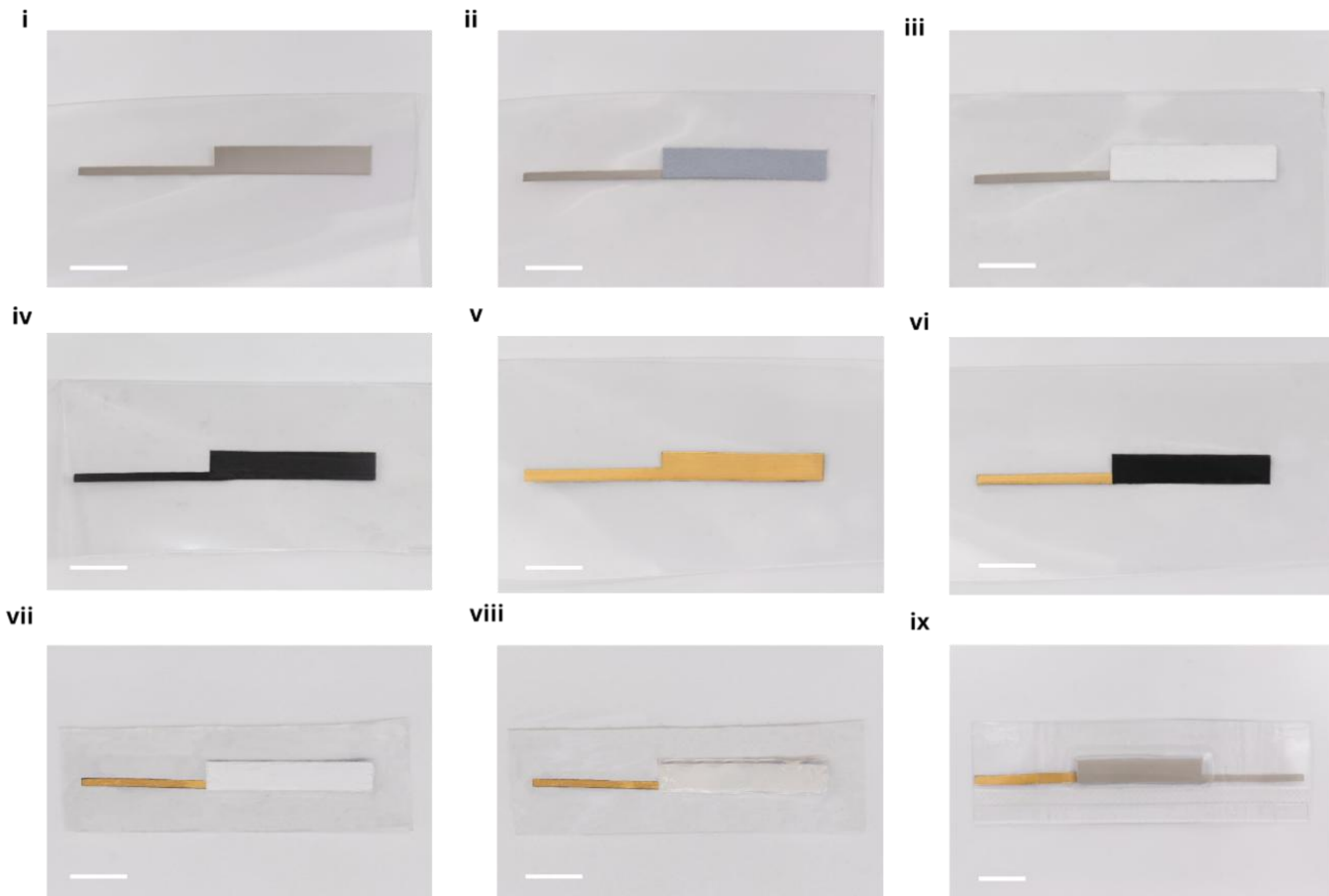

Fig. S25. Optical images of the layer-by-layer printing of the flexible AgO-Zn battery. (i) Ag current collector; (ii) Zn anode; (iii) TiO_2 separator; (iv) carbon current collector; (v) Sputtered Au; (vi) AgO cathode; (vii) cellulose separator; (viii) hydrogel electrolyte; (ix) assembled battery. Scale bar: 1 cm.

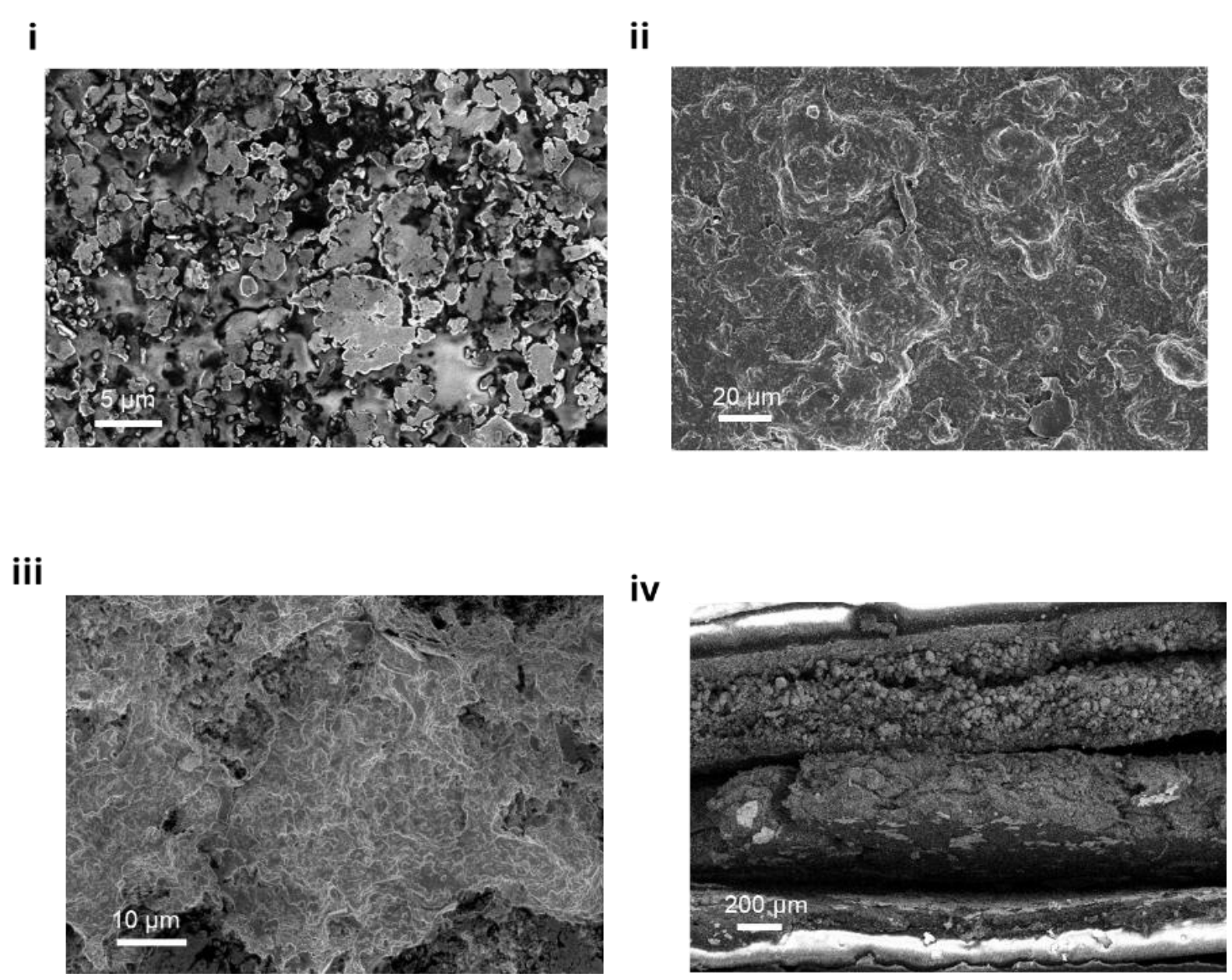

Fig. S26. SEM images of the Ag CC (i), carbon CC (ii), gold sputtered CC (iii) layers and full cell (iv) of the flexible AgO-Zn battery.

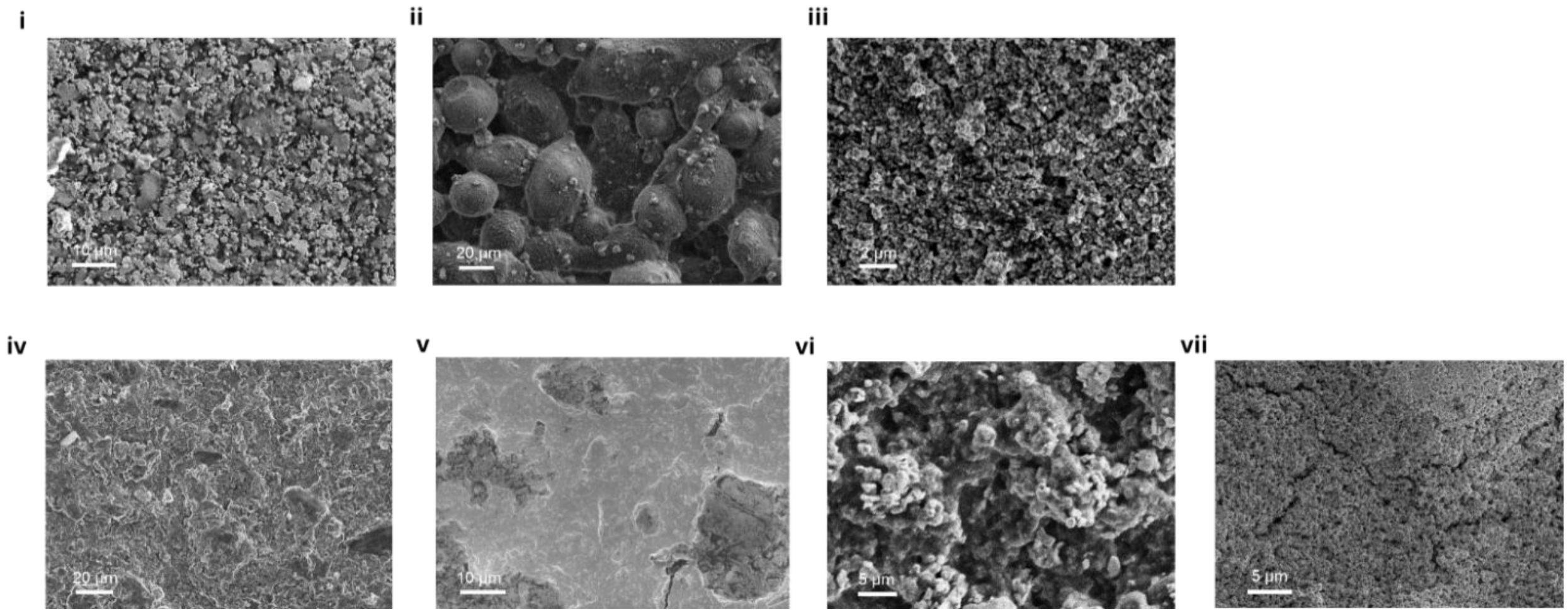

Fig. S27. SEM images of the different layers of the flexible AgO-Zn battery after bending. (i) AgCC; (ii) zinc anode; (iii) TiO_2 separator; (iv) carbon CC; (v) gold sputter; (vi) AgO; (vii) cellulose separator.

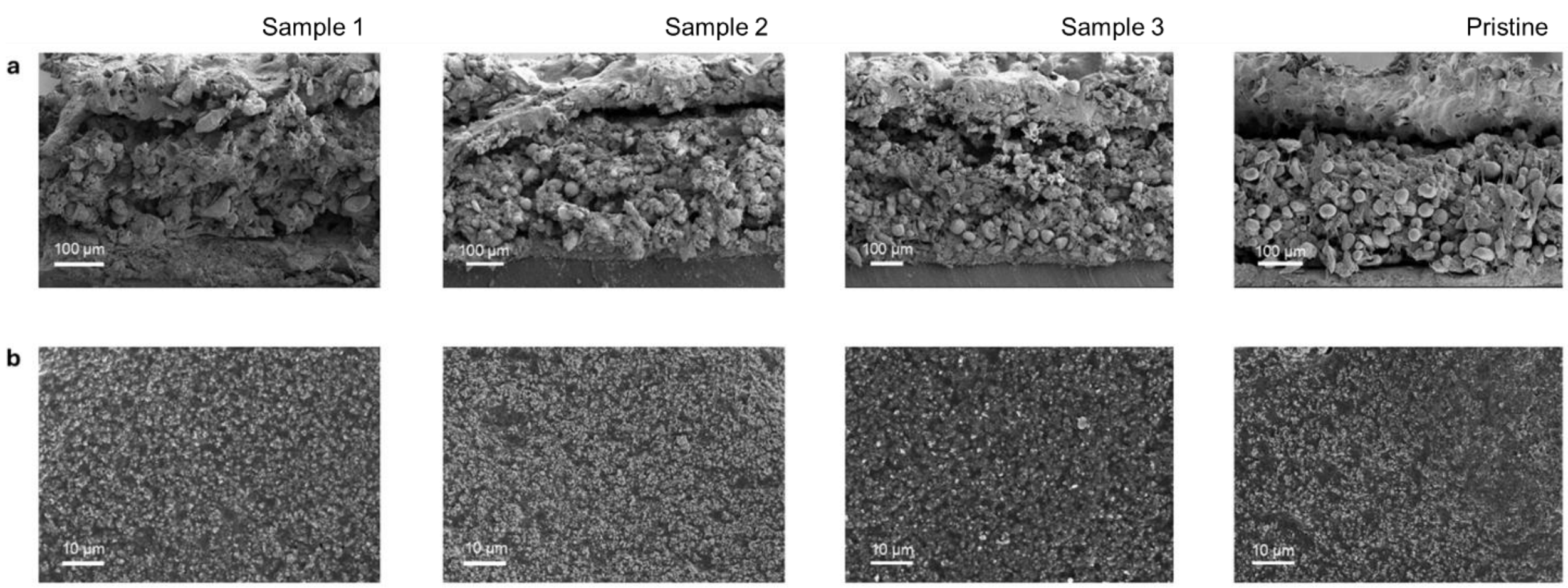

Fig. S28. a) SEM images of the cross-section of the anode side in one pristine battery and in three different battery samples after 20 cycles. The top layer is the TiO_2 separator layer, the layer in the middle is the zinc anode layer, and the bottom layer is the silver current collector and the SEBS substrate. b) SEM images of the surface of the TiO_2 separators in one pristine battery and in three different battery samples after 20 cycles.

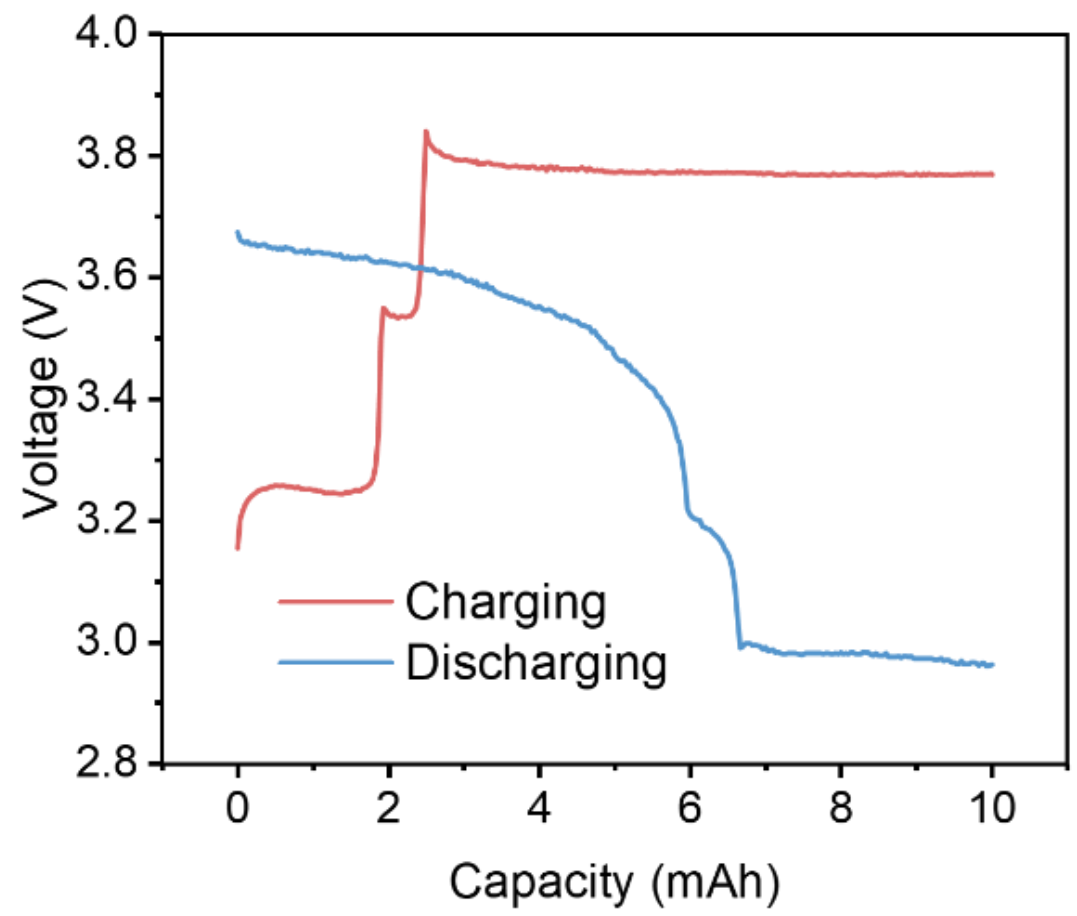

Fig. S29. (a) The voltage-capacity plot of the in series connected batteries.

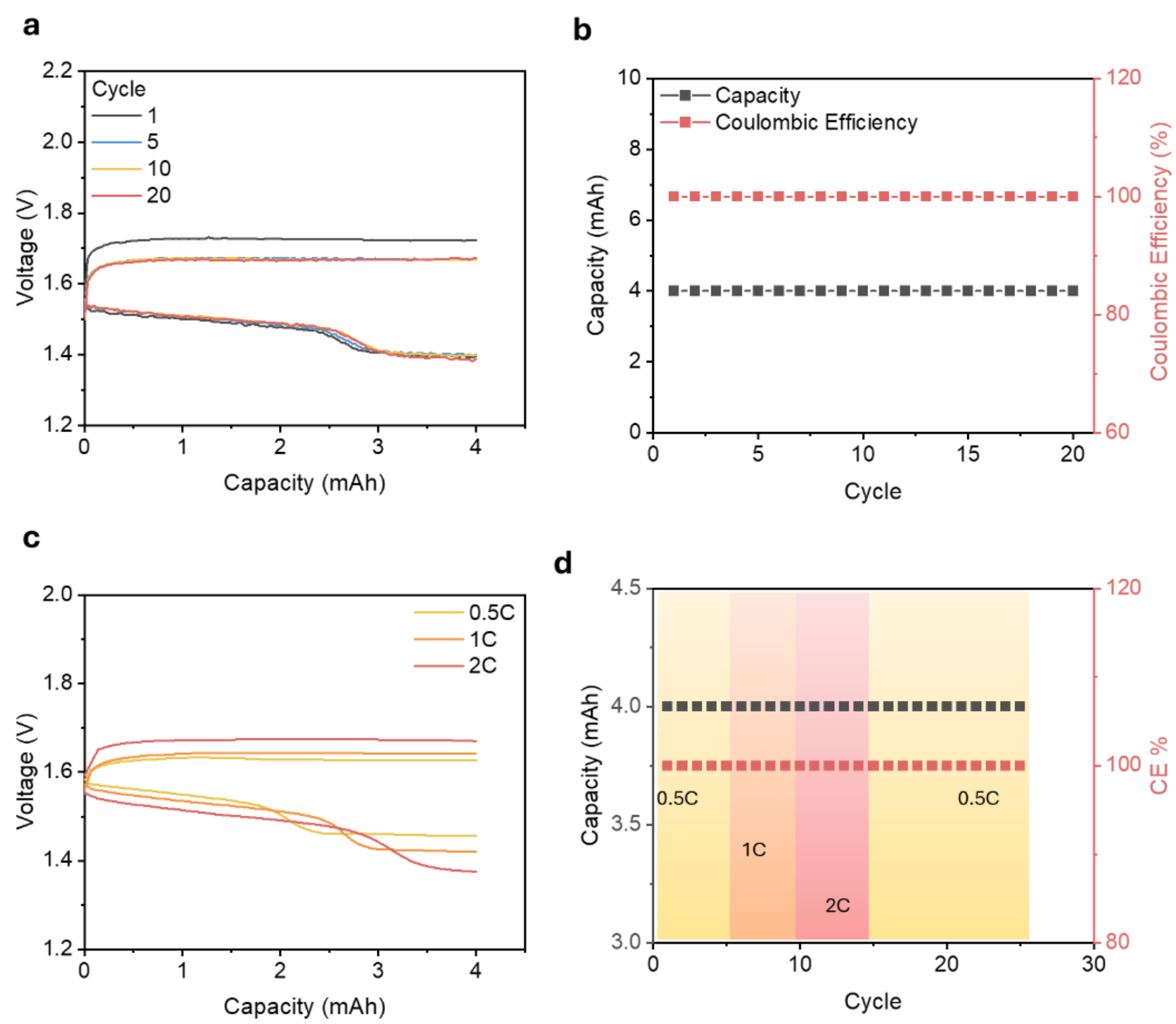

Fig. S30. (a) The voltage-capacity plot of the battery under 0.5 C-rate. (b) The cycling performance of the battery at charging and discharging rates of 0.5 C. (c) The voltage–capacity plot of the AgO-Zn flexible battery under different discharging C-rate. (d) The cycling performance of the battery at different charging and discharging C-rates.

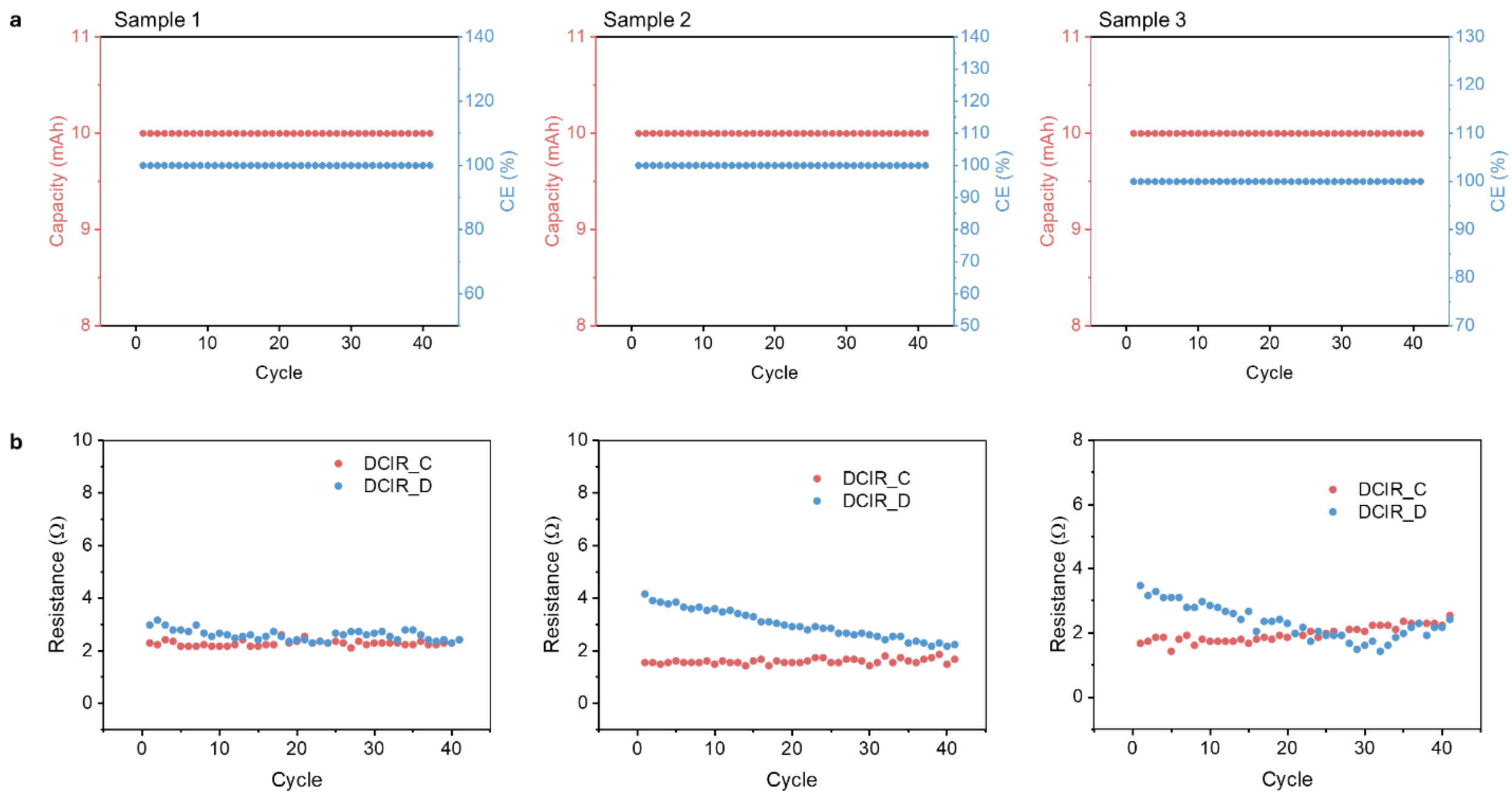

Fig. S31. a) The charging capacity and coulombic efficiency of two other battery samples at cycling rates of 0.5 C, in addition to the battery sample shown in Figure 4e. b) The DCIR of two other AgO-Zn battery samples at the C-rate of 0.5 C, in addition to the battery sample shown in Figure 4f.

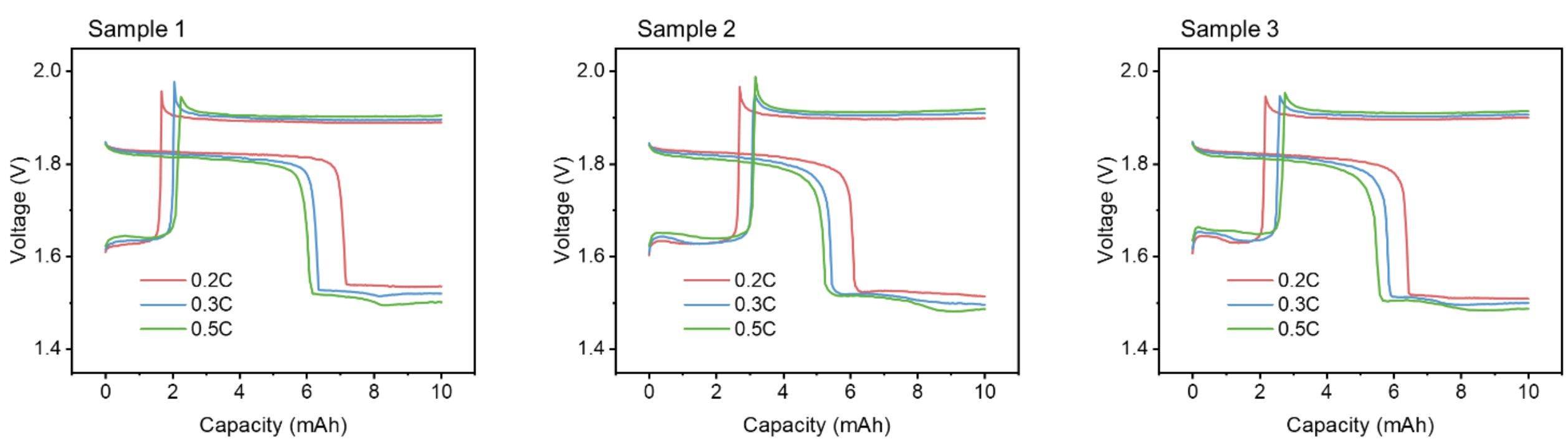

Fig. S32. The voltage-capacity plot of 3 different battery samples charging and discharging under different C-rates.

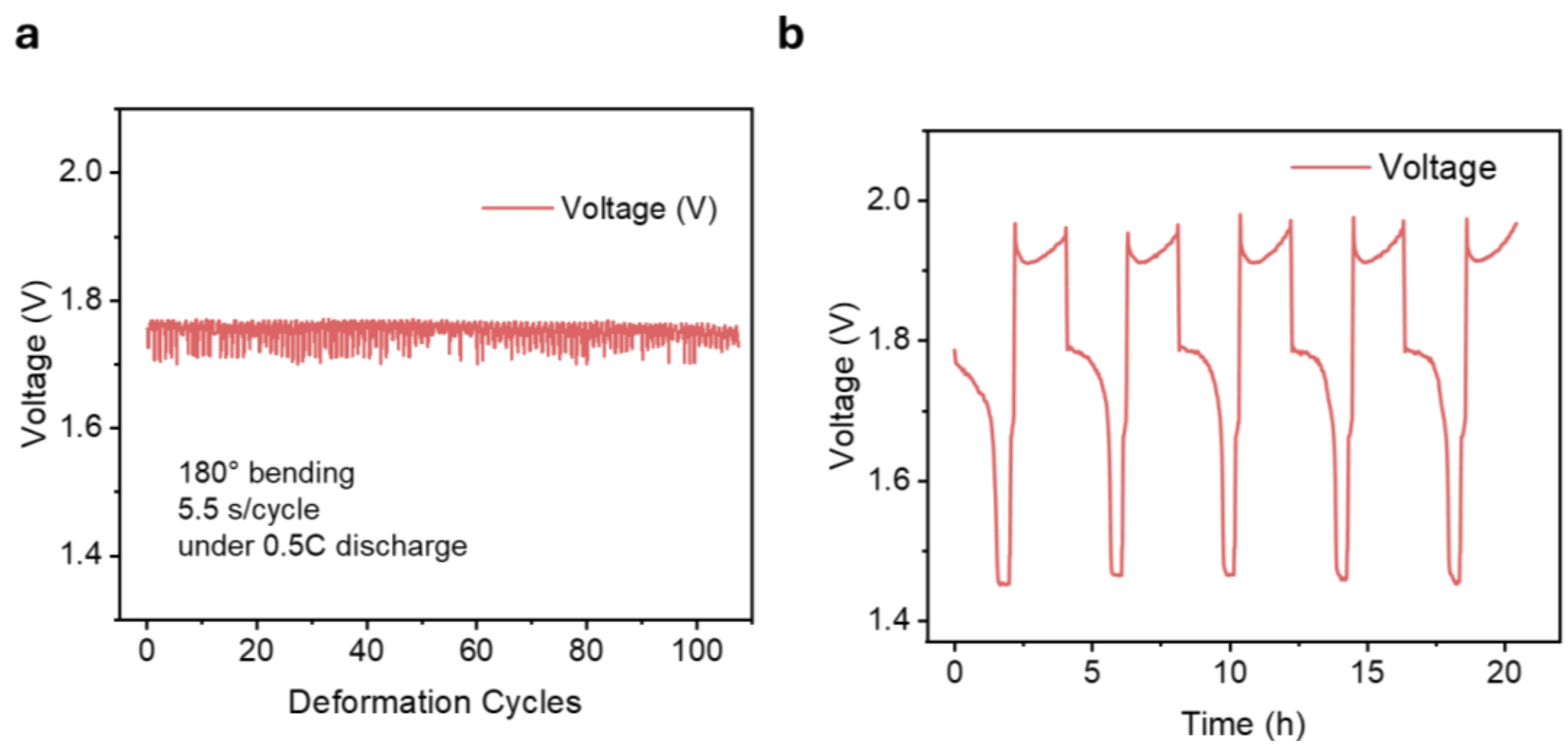

Fig. S33. (a) The voltage of AgO-Zn battery under 0.5C discharging intensity as well as under 180° bending. (b)The voltage-time plot of battery for 5 repeat charging and discharging cycles under 90° bending.

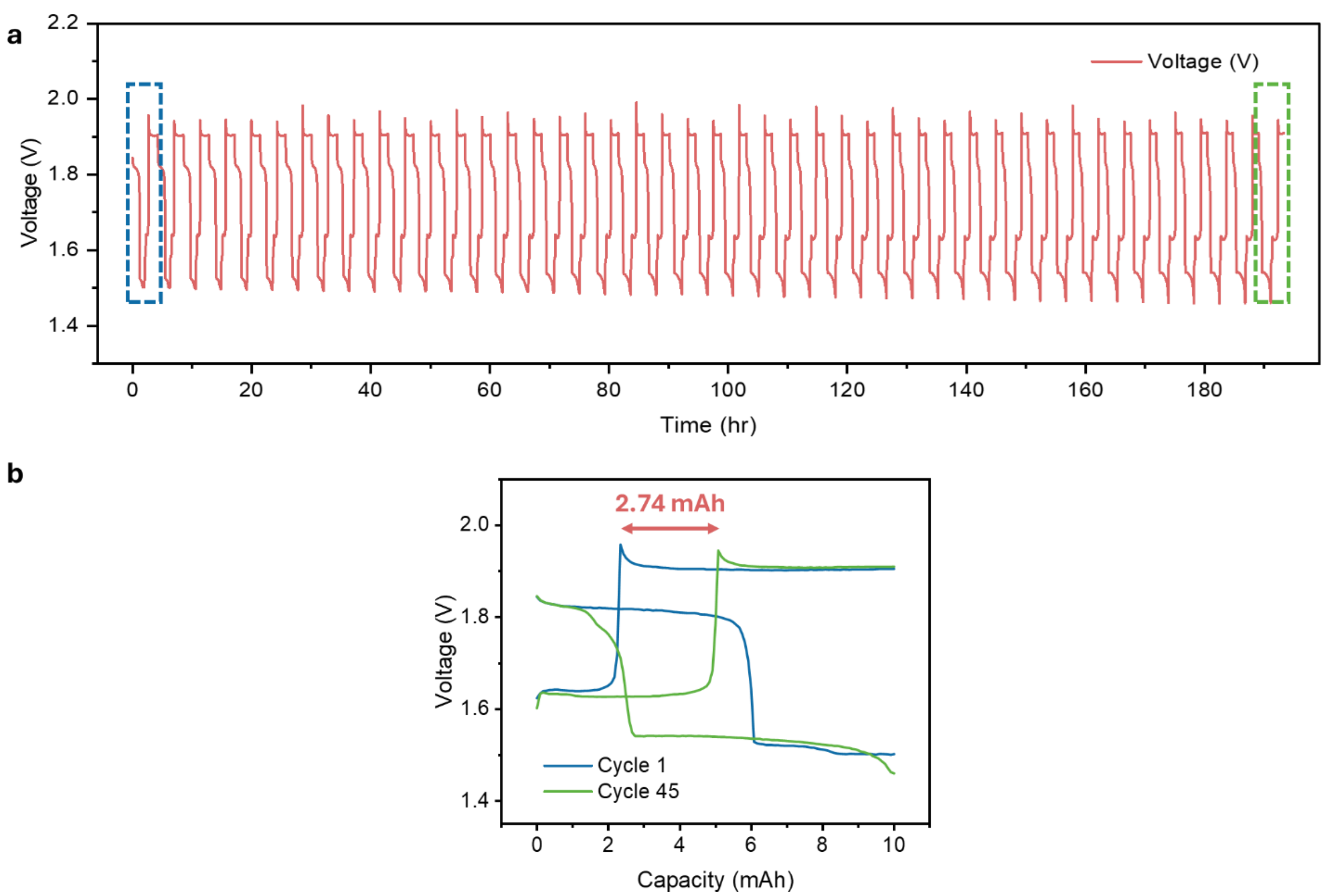

Fig. S34. Self-discharge behavior of the battery. (a) Voltage profile of the battery over 45 continuous cycles at 0.5C. The blue box indicates where the first cycle is, and the green box indicates the 45th cycle. (b) Charge-discharge curves of the first cycle and the 45th

cycle. The difference between the capacity at which the charging curves enter the second voltage platform is shown, which indicates the amount of charge lost due to self-discharge.

Supplementary Note 9: Calibration model to acquire blood concentration dynamics from sensor's $\boldsymbol{I}(\boldsymbol{t})$

The two-point calibration model (of $y(t) = ax(t) + b$ form) is developed by plotting minimum and maximum values of $\Delta I(t)$ vs $BG(t)$. The basis of this assumption is:

1. When $\Delta I(t) = 0$, $BG(t) = BG_f$
2. $SBG(t) \rightarrow BG(t)$ will give minimum mean absolute relative difference (MARD).

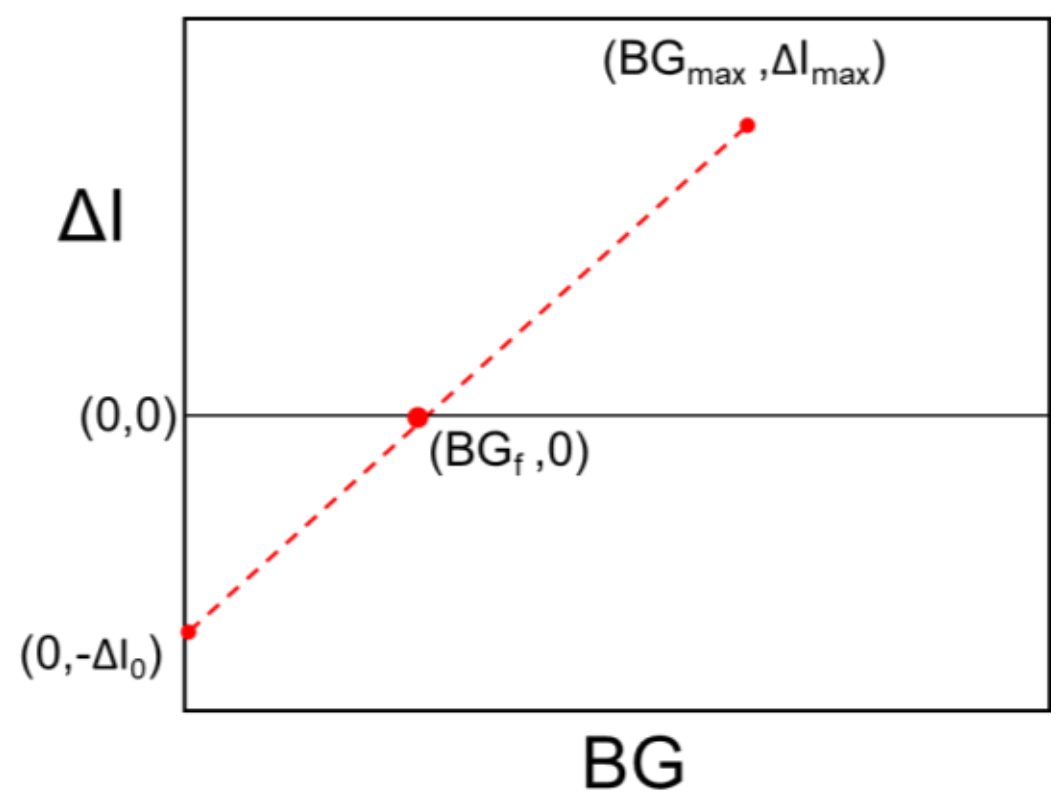

From the calibration curve, the corrected slope (a) and intercept (b) are given by:

$$a = \frac{\Delta I_{max}}{BG_{max} - BG_f}$$

$$b = -\Delta I_0$$

Therefore, $SBG\ (t) = x(t) = \frac{y(t)-b}{a} = \frac{I(t)-I_i+\Delta I_0}{a}$

After substituting all values, the final derived equation to convert $E(t)$ is:

$$\boldsymbol{SBG(t) = \left(\frac{I(t)-I_i+\Delta I_0}{\Delta I_{max}}\right)(BG_{max} - BG_f)}$$

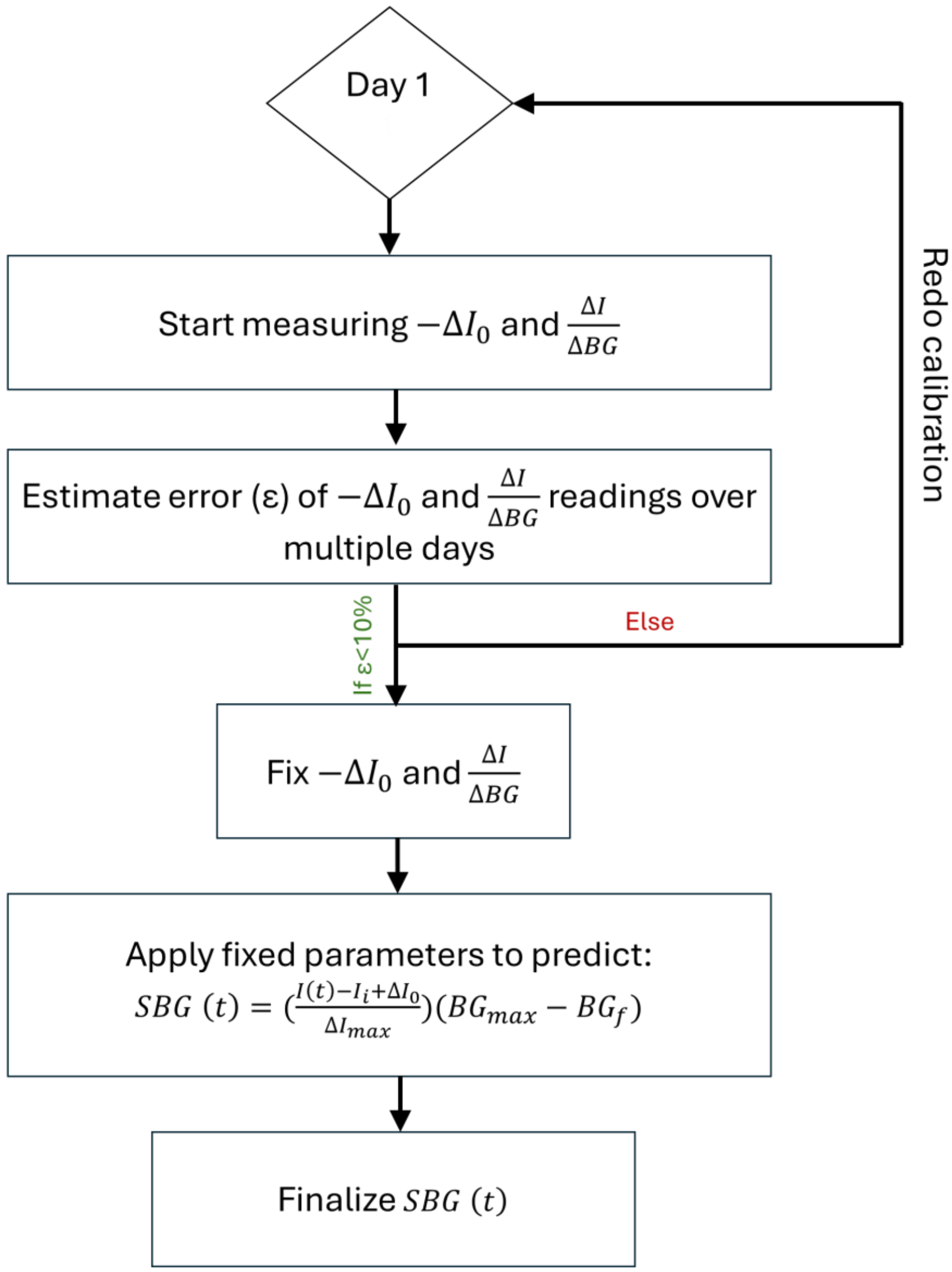

Fig. S35: Flow chart of personalized calibration protocol for glucose. The long-term stability of $-\Delta I_0$ and $\frac{\Delta I}{\Delta BG}$ under same stimulus determines the accuracy of $SBG\ (t)$ measurements.

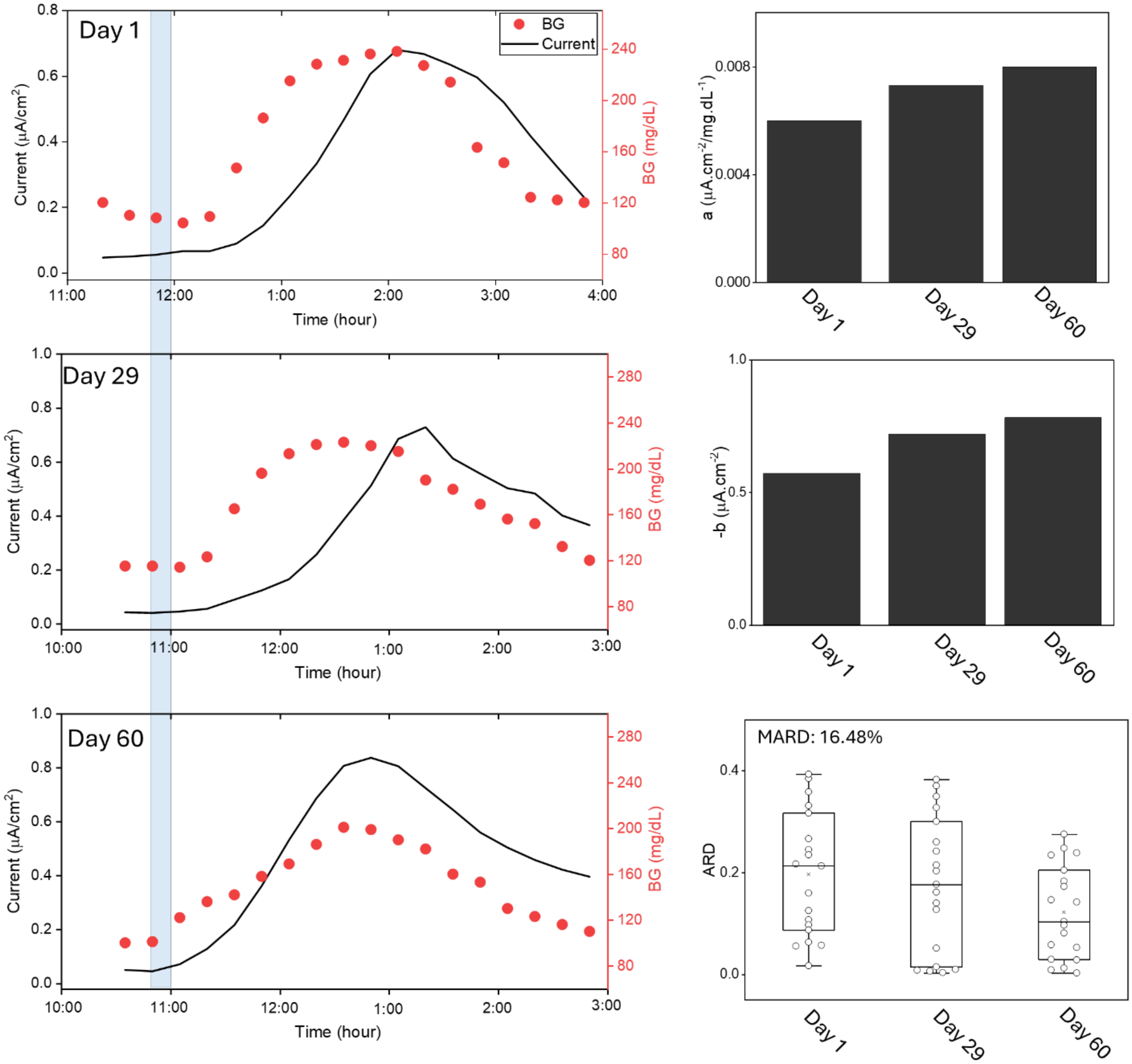

Fig. S36: Glucose profile of T1D subject over multiple days. Personalized coefficients a and b were evaluated on each day and their average ranged $0.007 \pm 0.001 \frac{\mu A.cm^{-2}}{mg.dL^{-1}}$ and $-0.69 \pm 0.107\ \mu A.cm^{-2}$, respectively. Using these values, the average MARD from three tests ranged ~16.48%. Blue zone: Meal intake timeline.

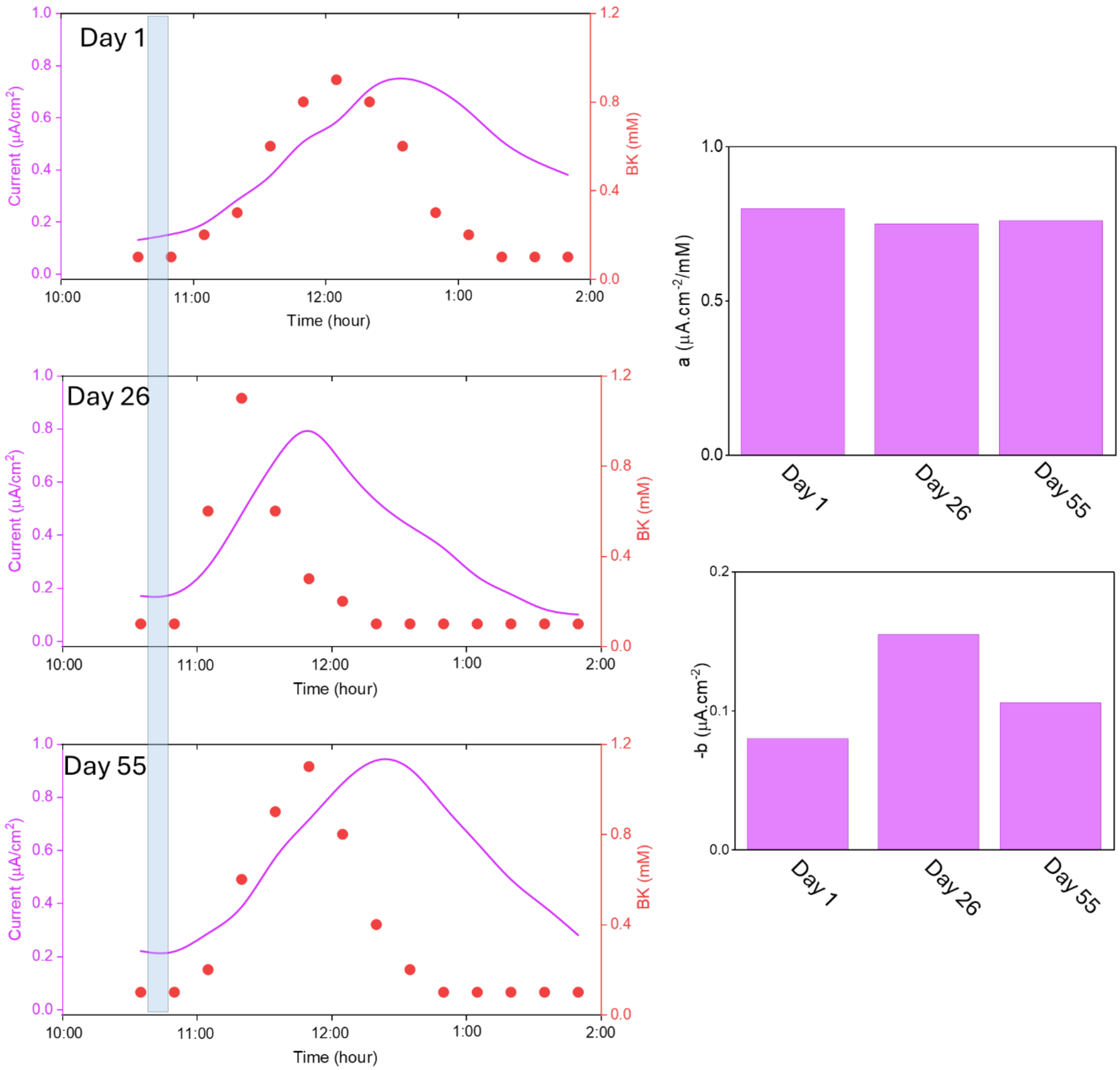

Fig. S37: Ketone profile of T1D subject over multiple days. Personalized coefficients a and b were evaluated on each day and their average ranged $0.074 \pm\ 0.027\ \frac{\mu A.cm^{-2}}{mg.dL^{-1}}$ and $-0.113 \pm\ 0.03\ \ \mu A.cm^{-2}$, respectively. Blue zone: Ketone drink intake timeline.

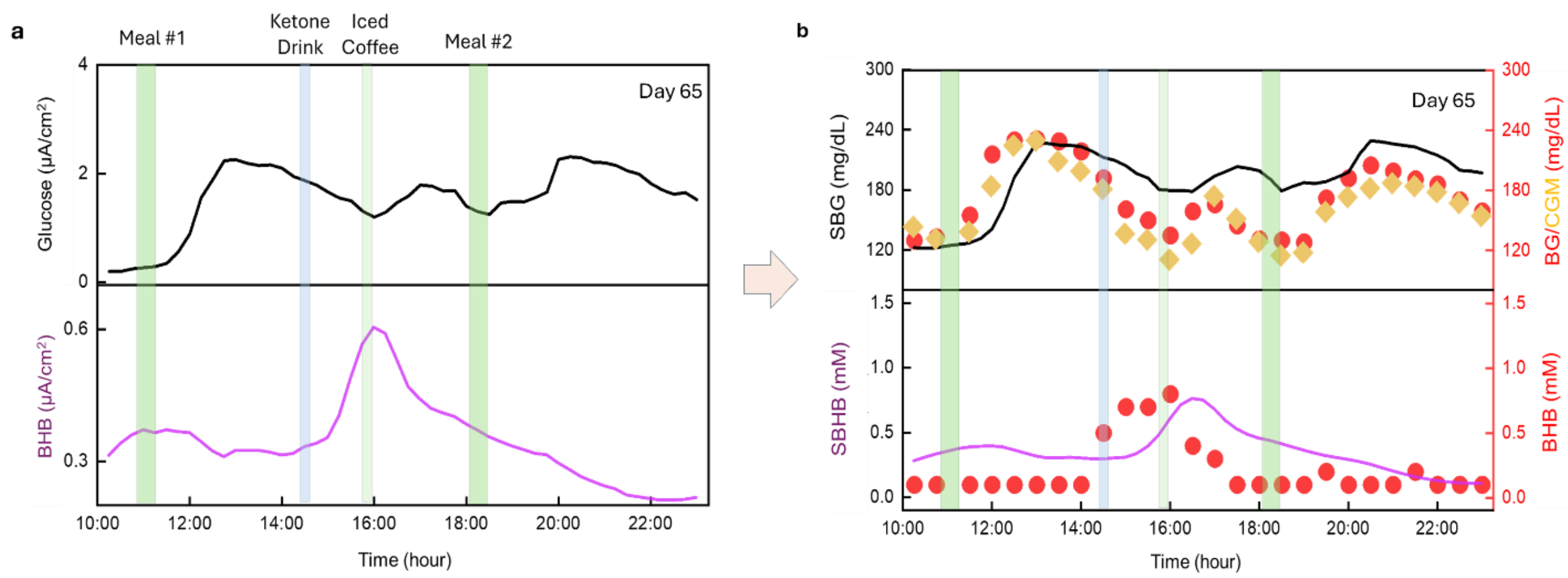

Fig. S38: Utilization of the personalized calibration protocol to convert current to concentration in a daily test. a) Personalized coefficients values from long-term calibration are plugged into the equation in Supplementary Note 9 to derive b) $SBG(t)$ and $SBHB(t)$.

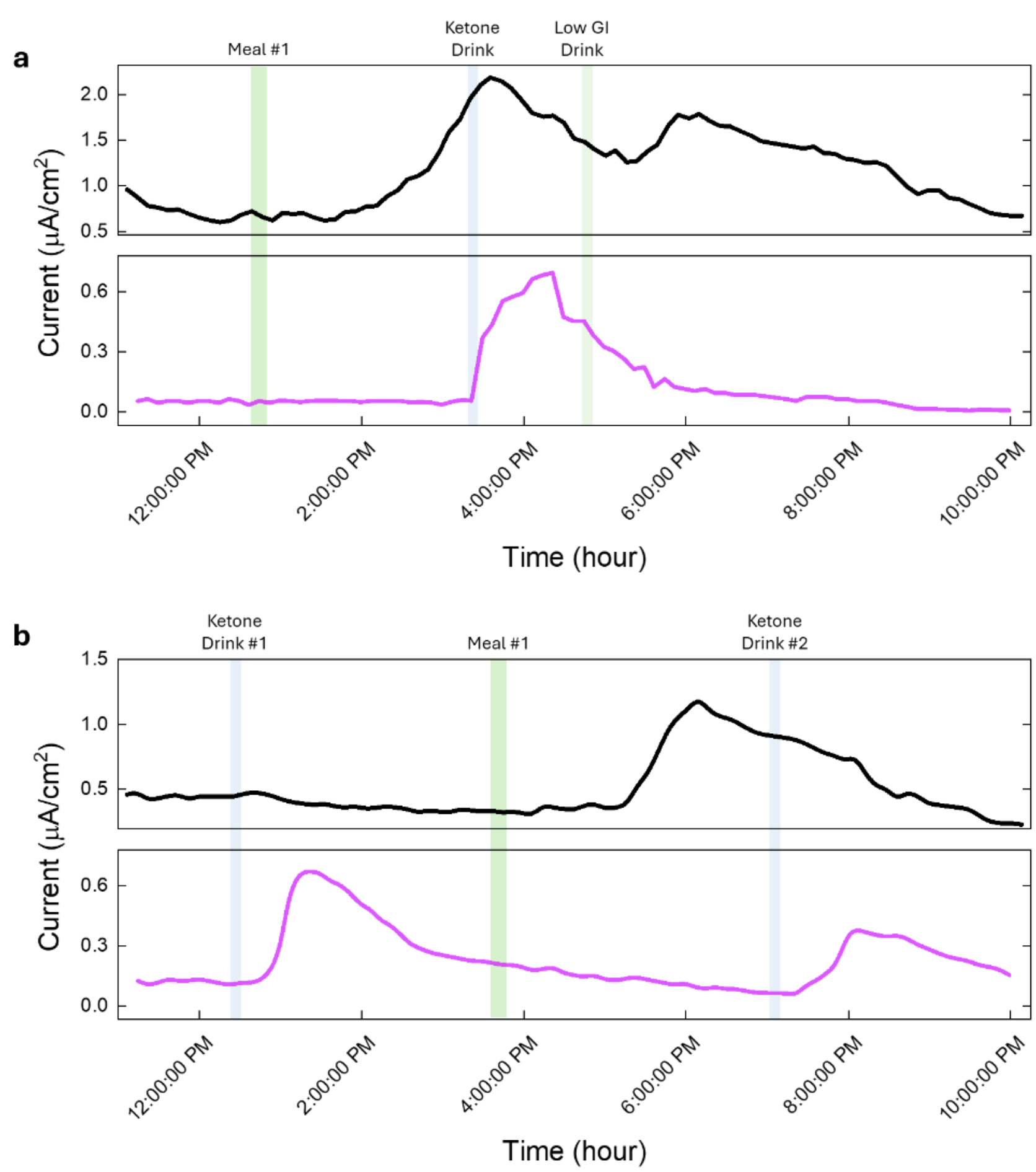

Fig. S39: Current profiles of glucose (black) and BHB (pink) from T1D subject related to Fig.5a i and ii. Black: glucose, Pink: Ketone.

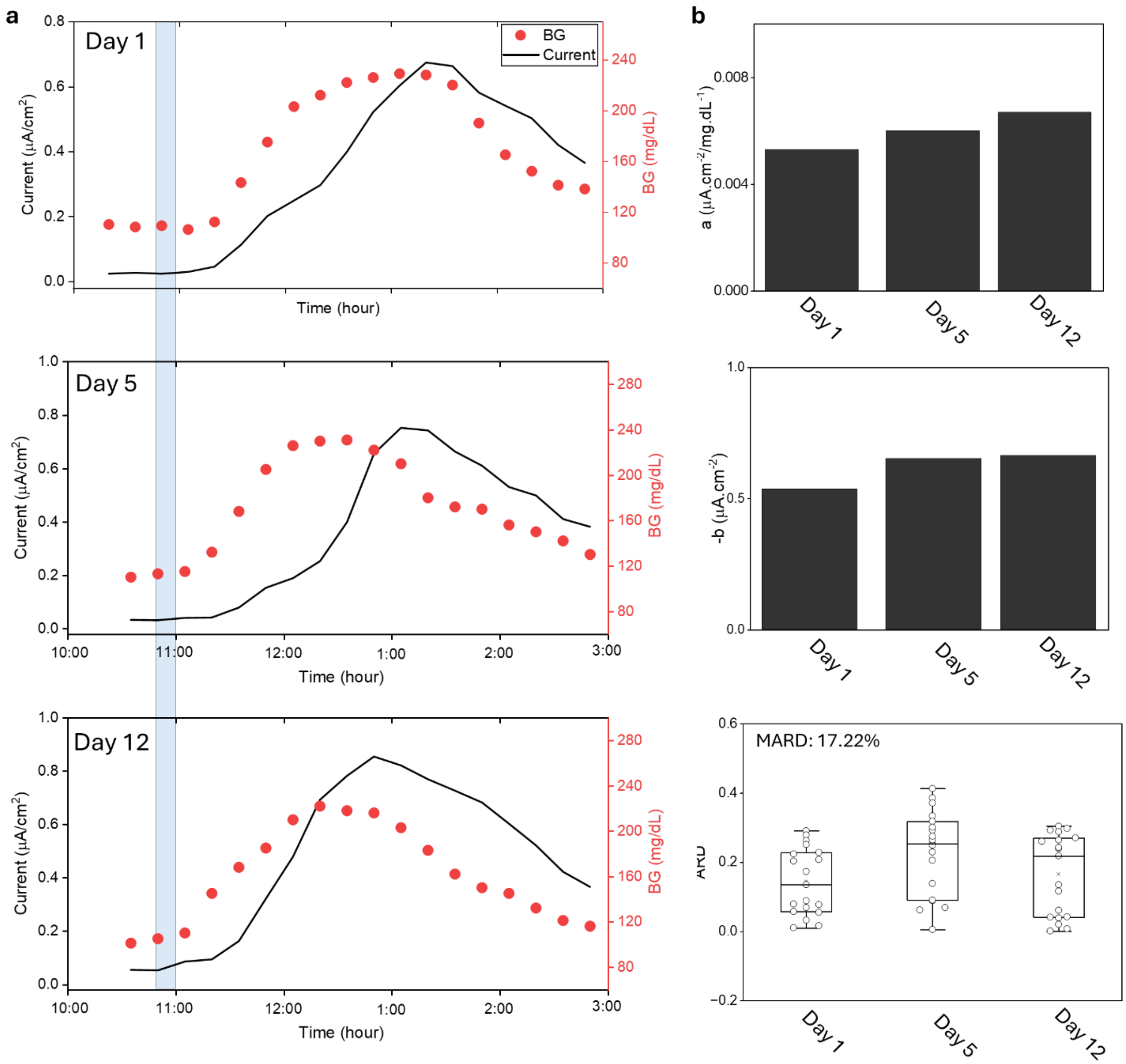

Fig. S40: Glucose profile of the second T1D subject over multiple days. Personalized coefficients a and b were evaluated on each day and their average ranged $0.006 \pm 0.0007\ \frac{\mu A.cm^{-2}}{mg.dL^{-1}}$ and $-0.62 \pm 0.07\ \mu A.cm^{-2}$, respectively. Using these values, the average MARD from three tests ranged ~17.22%. Blue zone: Meal intake timeline.

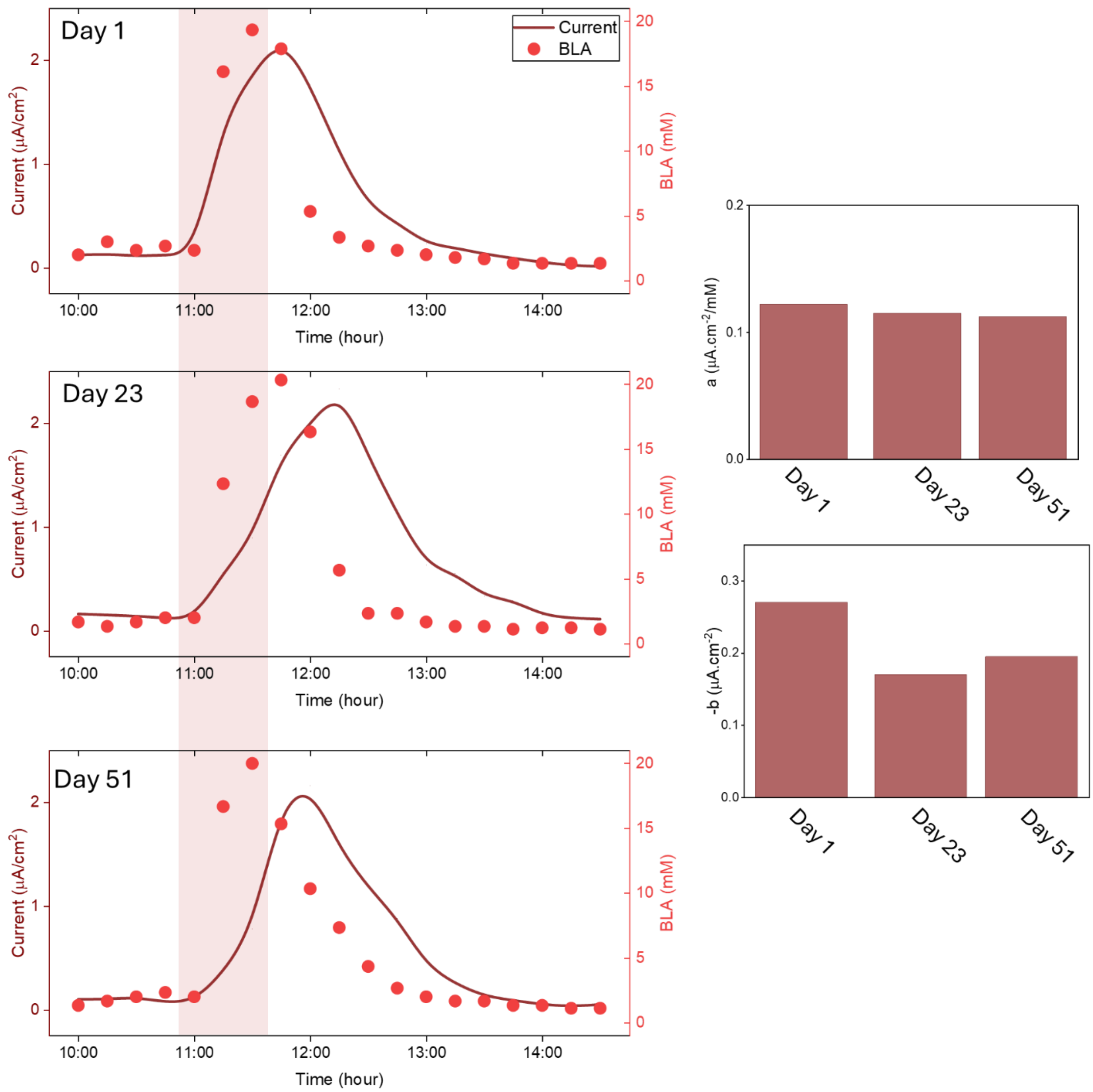

Fig. S41: Lactate profile of T1D subject over multiple days. Personalized coefficients a and b were evaluated on each day and their average ranged $0.116 \pm 0.005\ \frac{\mu A.cm^{-2}}{mM}$ and $-0.211 \pm 0.052\ \mu A.cm^{-2}$, respectively. Red zone: Exercising timeline.

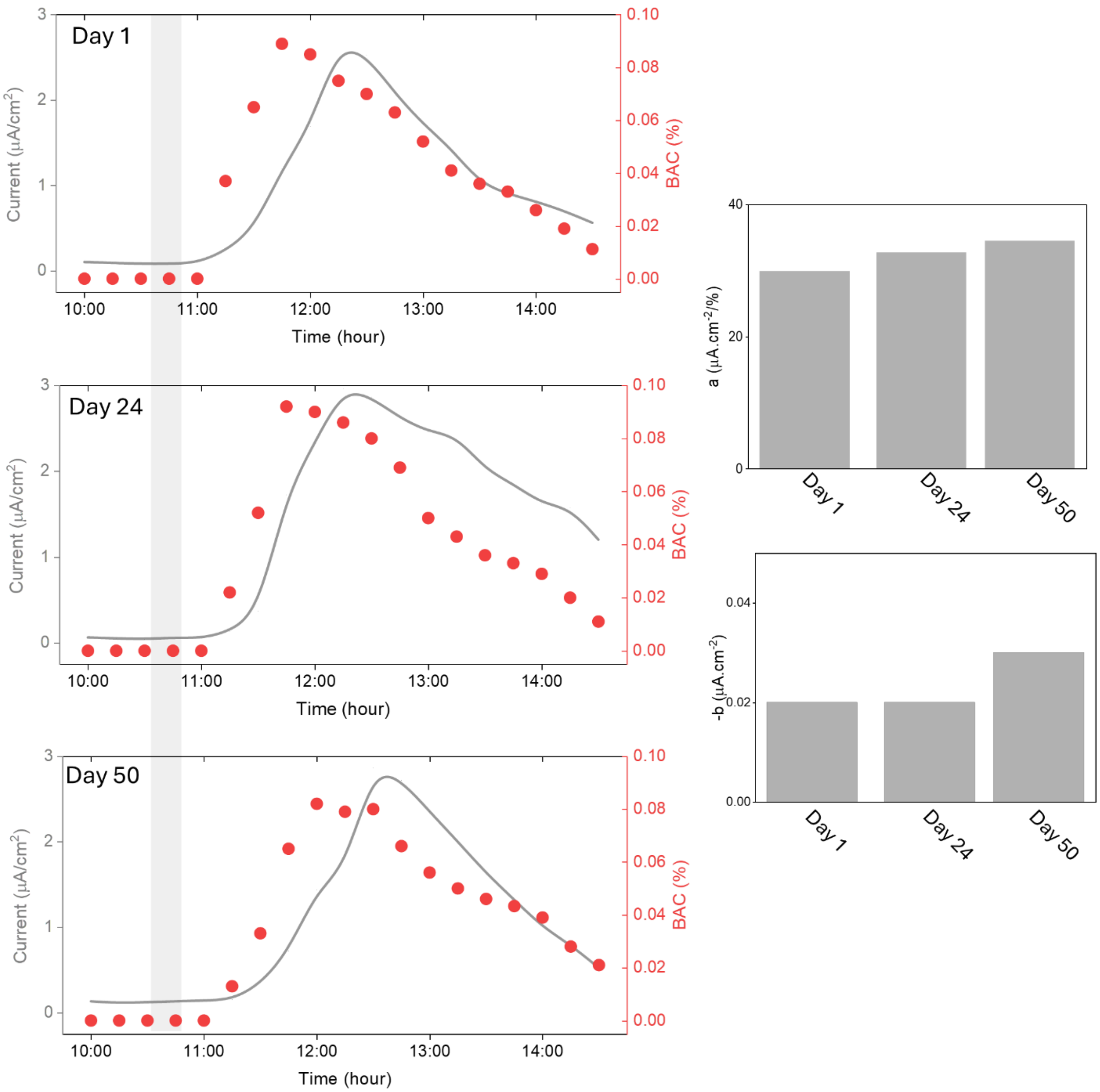

Fig. S42: Alcohol profile of T1D subject over multiple days. Personalized coefficients a and b were evaluated on each day and their average ranged $32.38 \pm 2.35 \frac{\mu A.cm^{-2}}{\%}$ and $-0.023 \pm 0.005\ \mu A.cm^{-2}$, respectively. Grey zone: Alcohol drink consumption timeline.

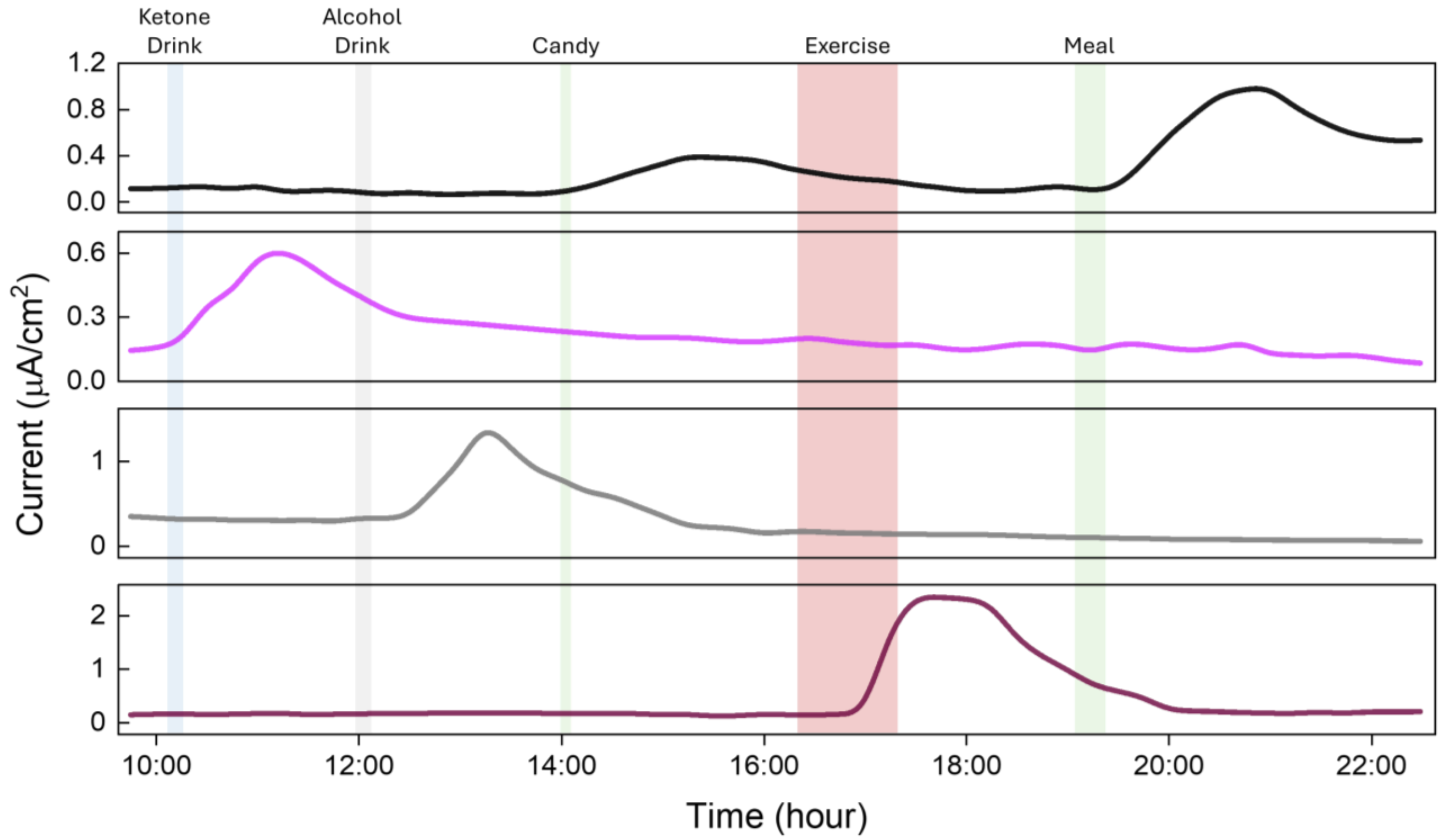

Fig. S43: Current profiles of glucose (black), BHB (pink), alcohol (grey) and lactate (brown) from T1D subject related to Fig.5b.

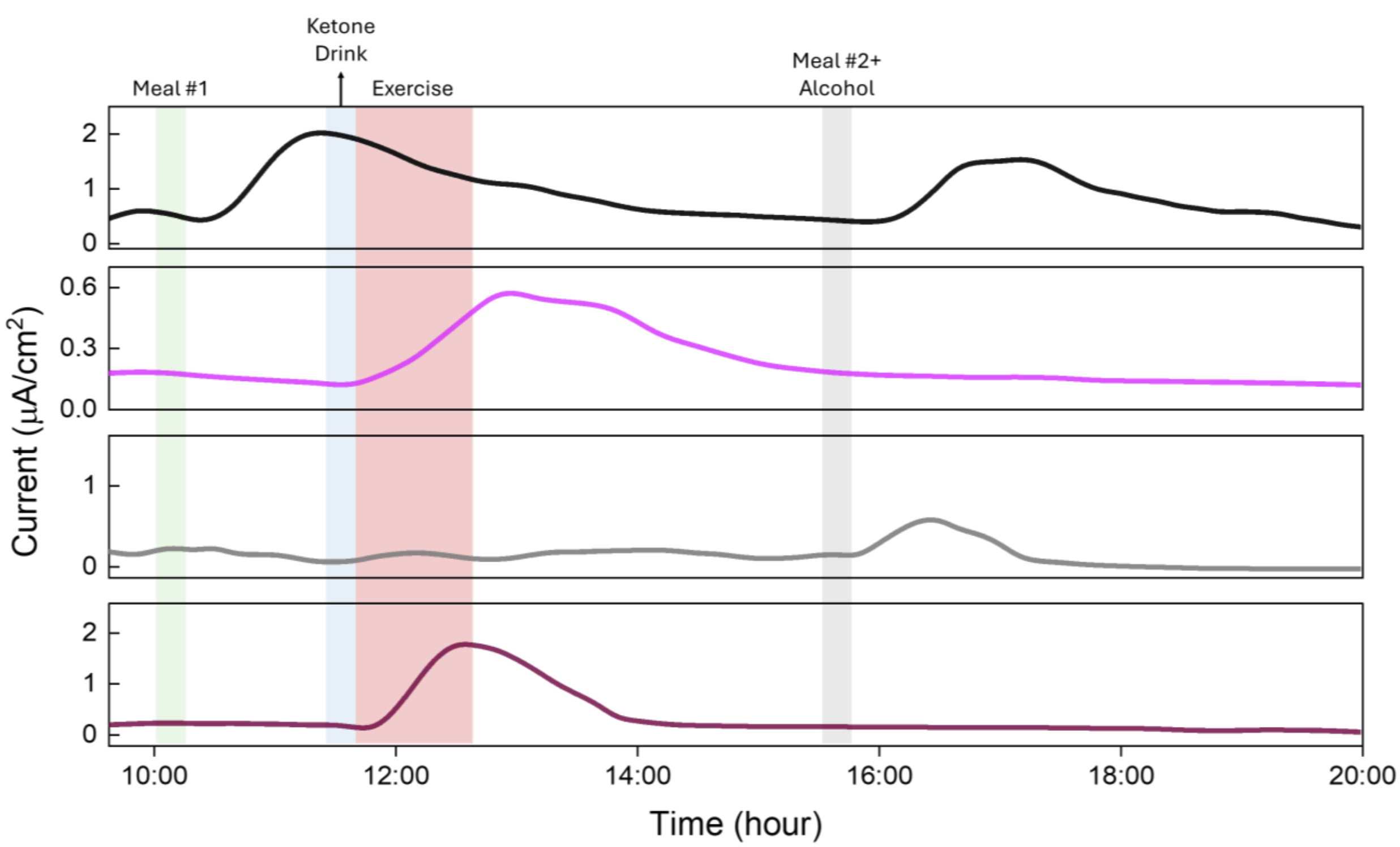

Fig. S44: Current profiles of glucose, BHB, alcohol and lactate from T1D subject related to Fig.5c.

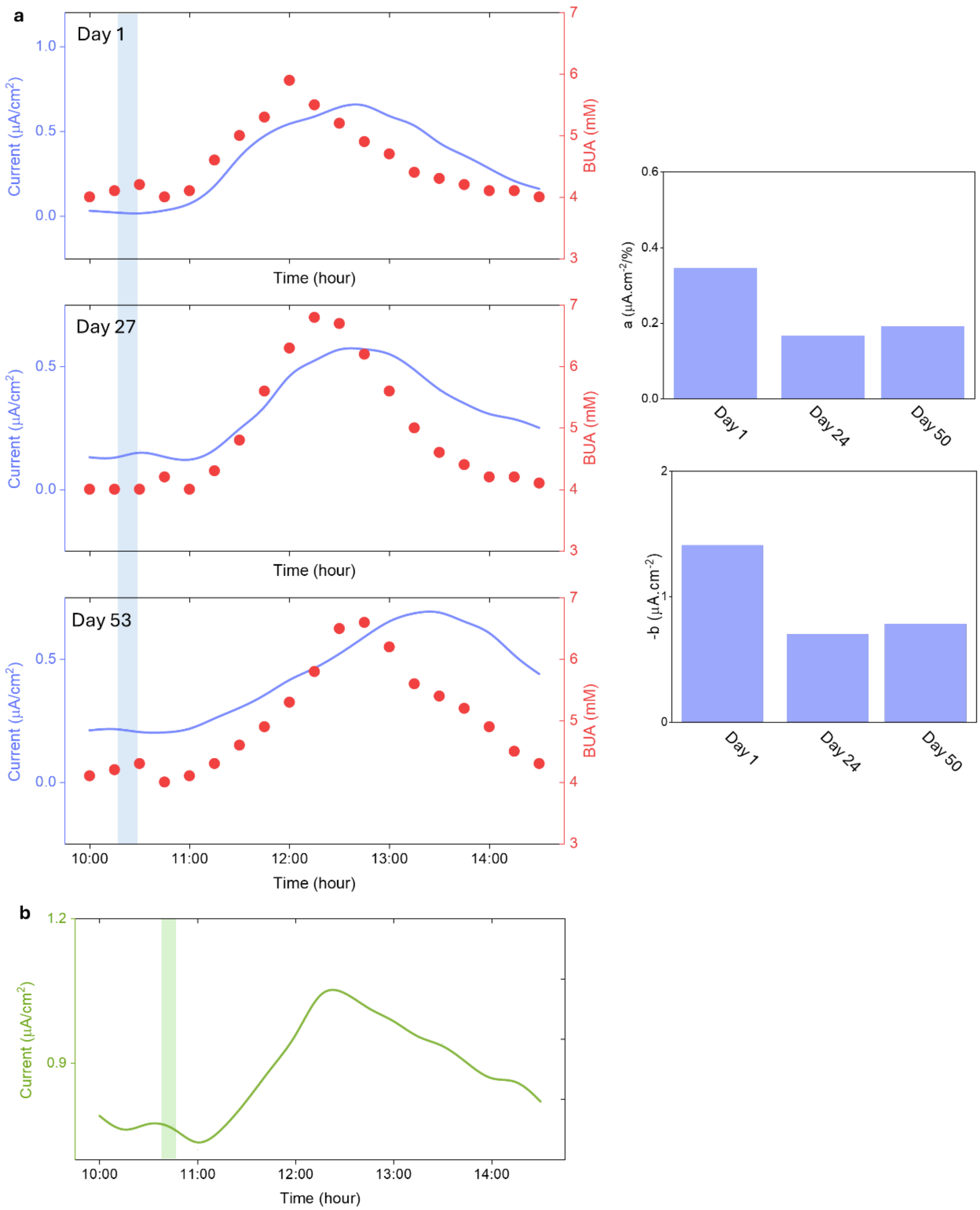

Fig. S45: UA (blue) and VC (green) profile of T1D subject over multiple days. Personalized coefficients a and b were evaluated on each day and their average ranged $0.235 \pm 0.097 \frac{\mu A.cm^{-2}}{mM}$ and $-0.95 \pm 0.36\ \mu A.cm^{-2}$, respectively for UA. Blue zone: Sardine consumption timeline. Green zone: Vit C drink consumption timeline.

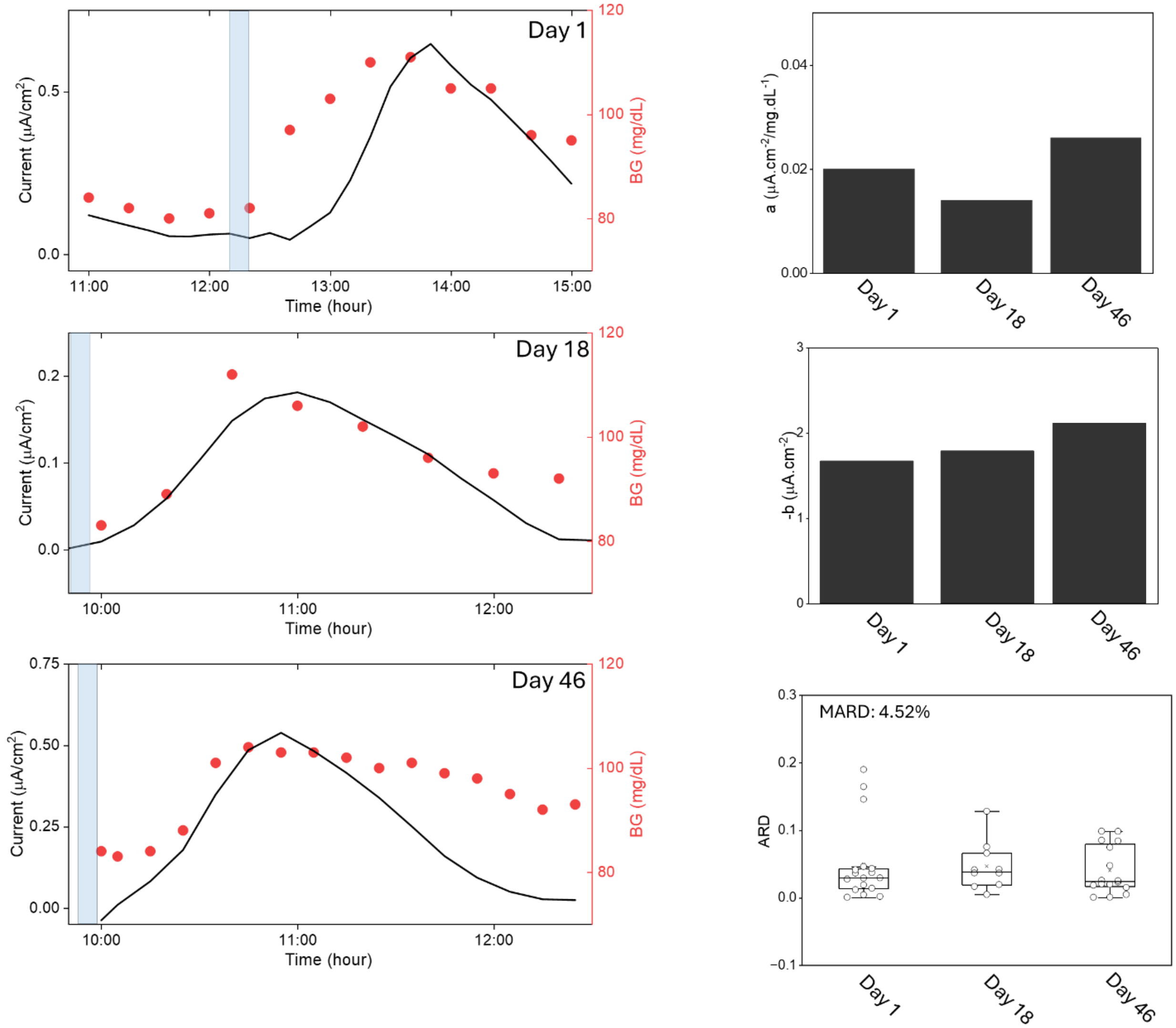

Fig. S46: Glucose profile of healthy subject over multiple days. Personalized coefficients a and b were evaluated on each day and their average ranged $0.02 \pm 0.006\ \frac{\mu A.cm^{-2}}{mg.dL^{-1}}$ and $-1.67 \pm 0.67\ \mu A.cm^{-2}$, respectively. Using these values, the average MARD from three tests ranged ~4.52 %. Blue zone: Meal intake timeline.

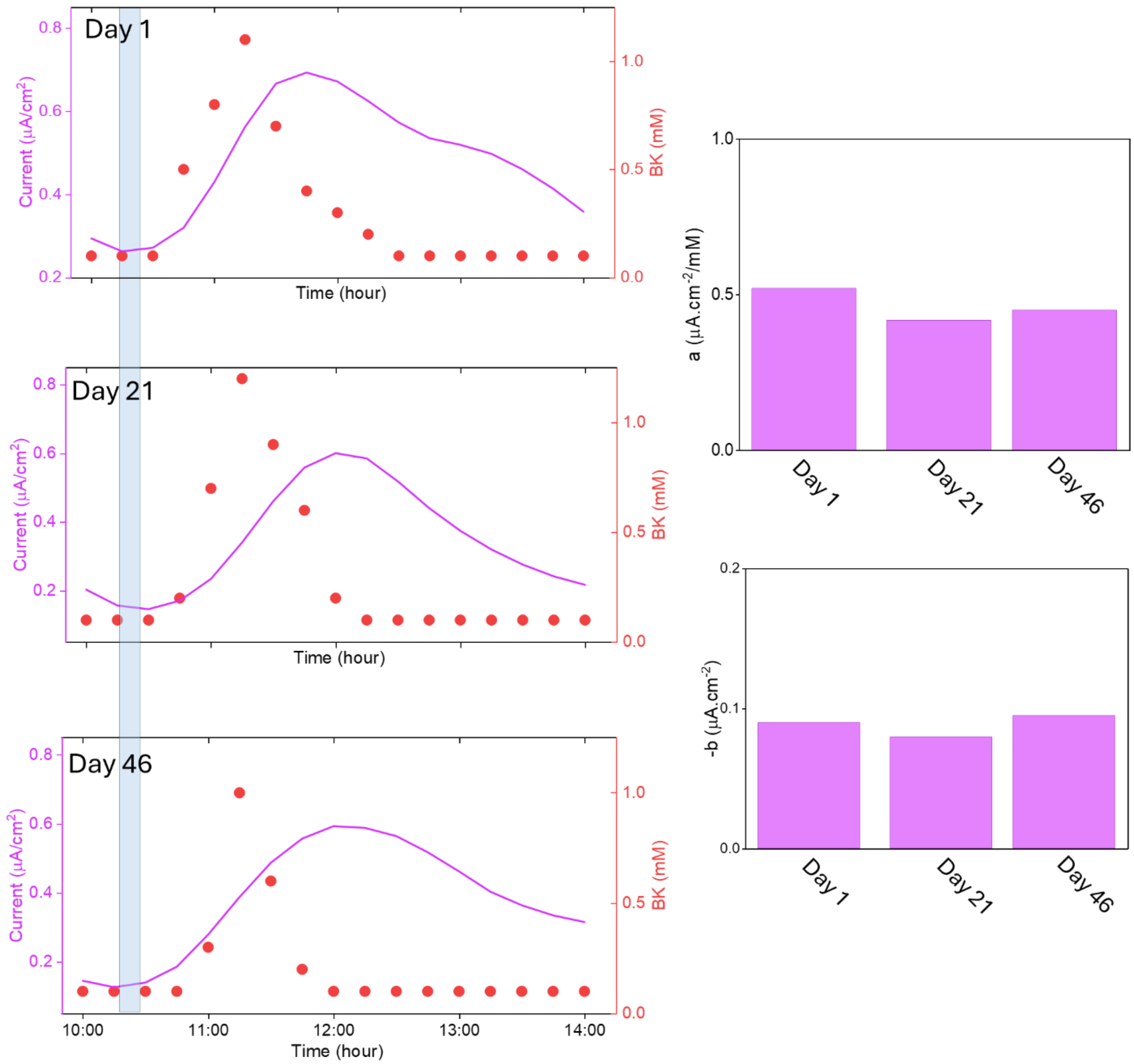

Fig. S47: BHB profile of healthy subject over multiple days. Personalized coefficients a and b were evaluated on each day and their average ranged $0.46 \pm 0.06\ \frac{\mu A.cm^{-2}}{mM}$ and $-0.08 \pm 0.008\ \mu A.cm^{-2}$, respectively. Blue zone: BHB intake timeline.

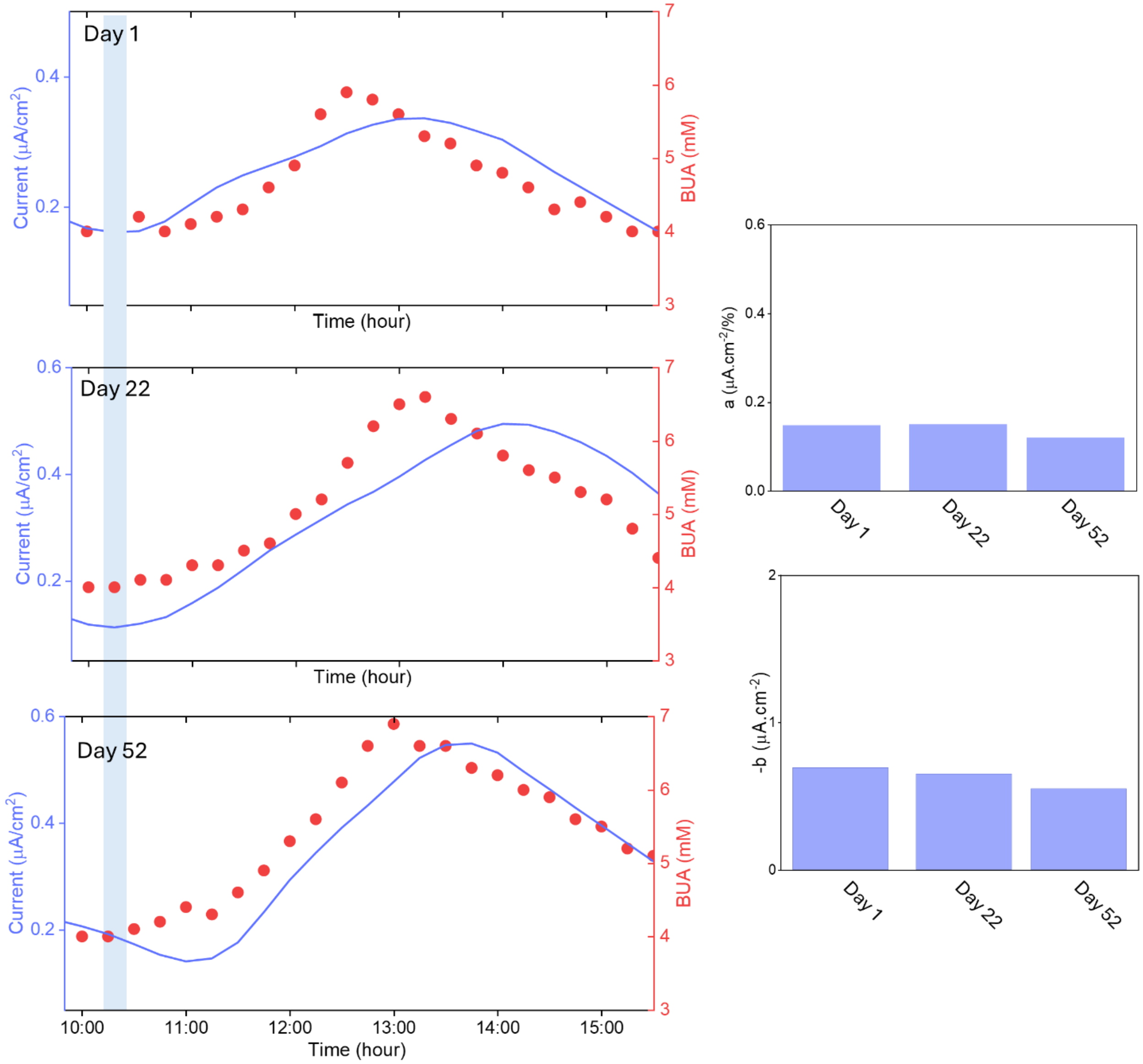

Fig. S48: UA profile of healthy subject over multiple days. Personalized coefficients a and b were evaluated on each day and their average ranged $0.13 \pm 0.012\ \frac{\mu A.cm^{-2}}{mM}$ and $-0.59 \pm 0.123\ \mu A.\,cm^{-2}$, respectively. Blue zone: Sardines intake timeline.

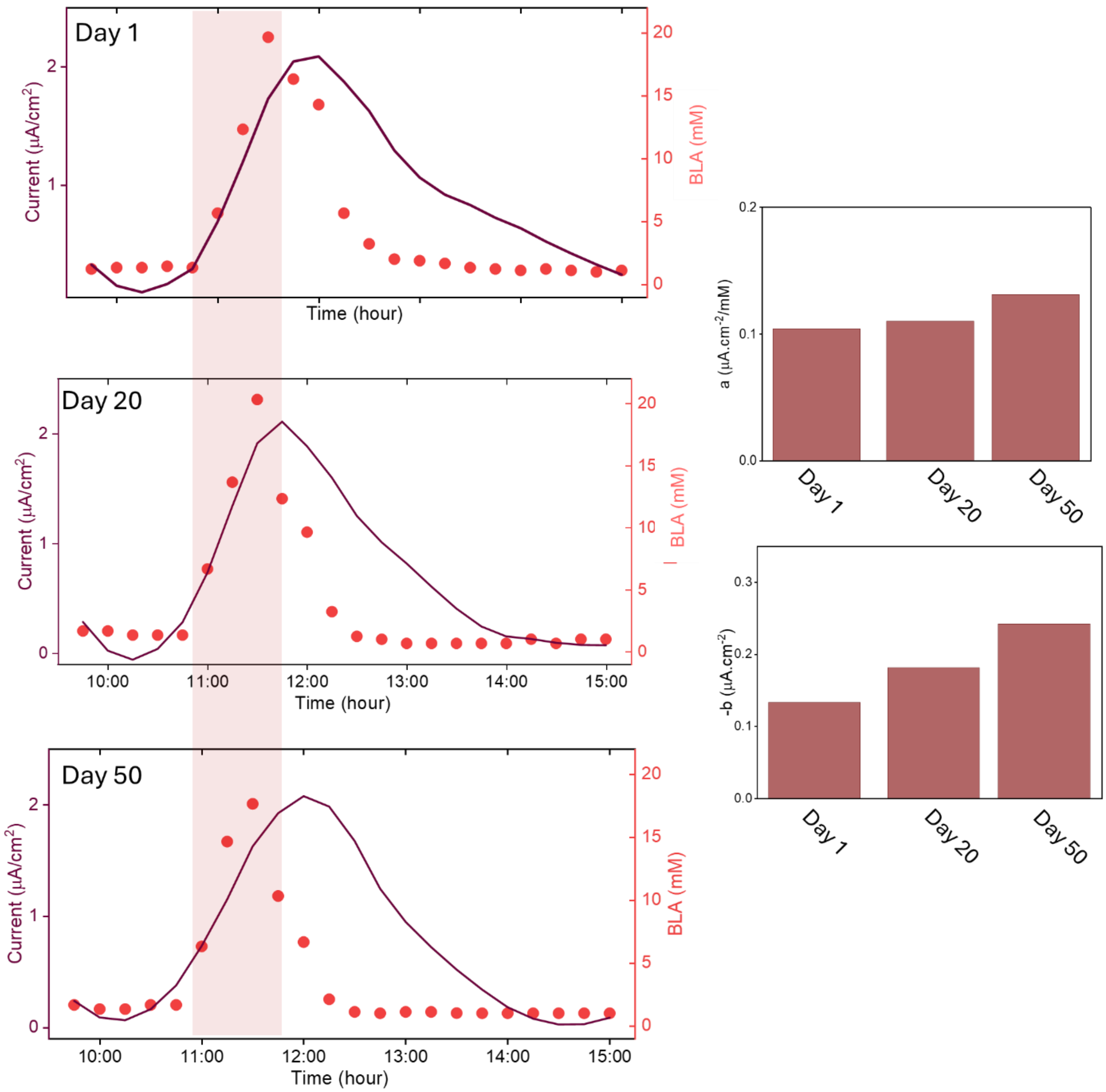

Fig. S49: Lactate profile of healthy subject over multiple days. Personalized coefficients a and b were evaluated on each day and their average ranged $0.11 \pm 0.014 \frac{\mu A.cm^{-2}}{mM}$ and $-0.18 \pm 0.054\ \mu A.cm^{-2}$, respectively. Blue zone: Exercise timeline.

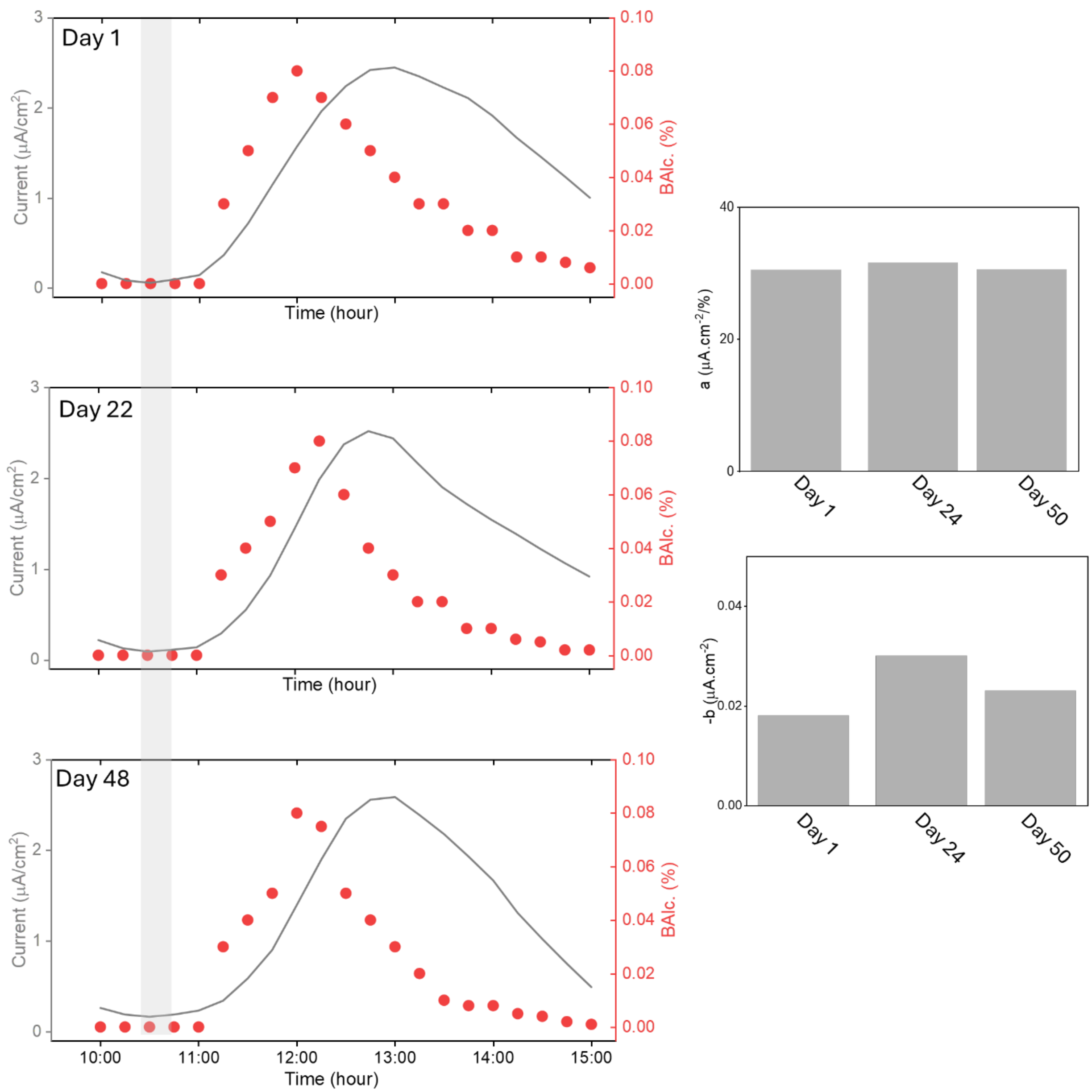

Fig. S50: Alcohol profile of healthy subject over multiple days. Personalized coefficients a and b were evaluated on each day and their average ranged $30.73 \pm 1.821\ \frac{\mu A.cm^{-2}}{\%}$ and $-0.02 \pm 0.017\ \mu A.cm^{-2}$, respectively. Blue zone: Alcohol drink intake timeline.

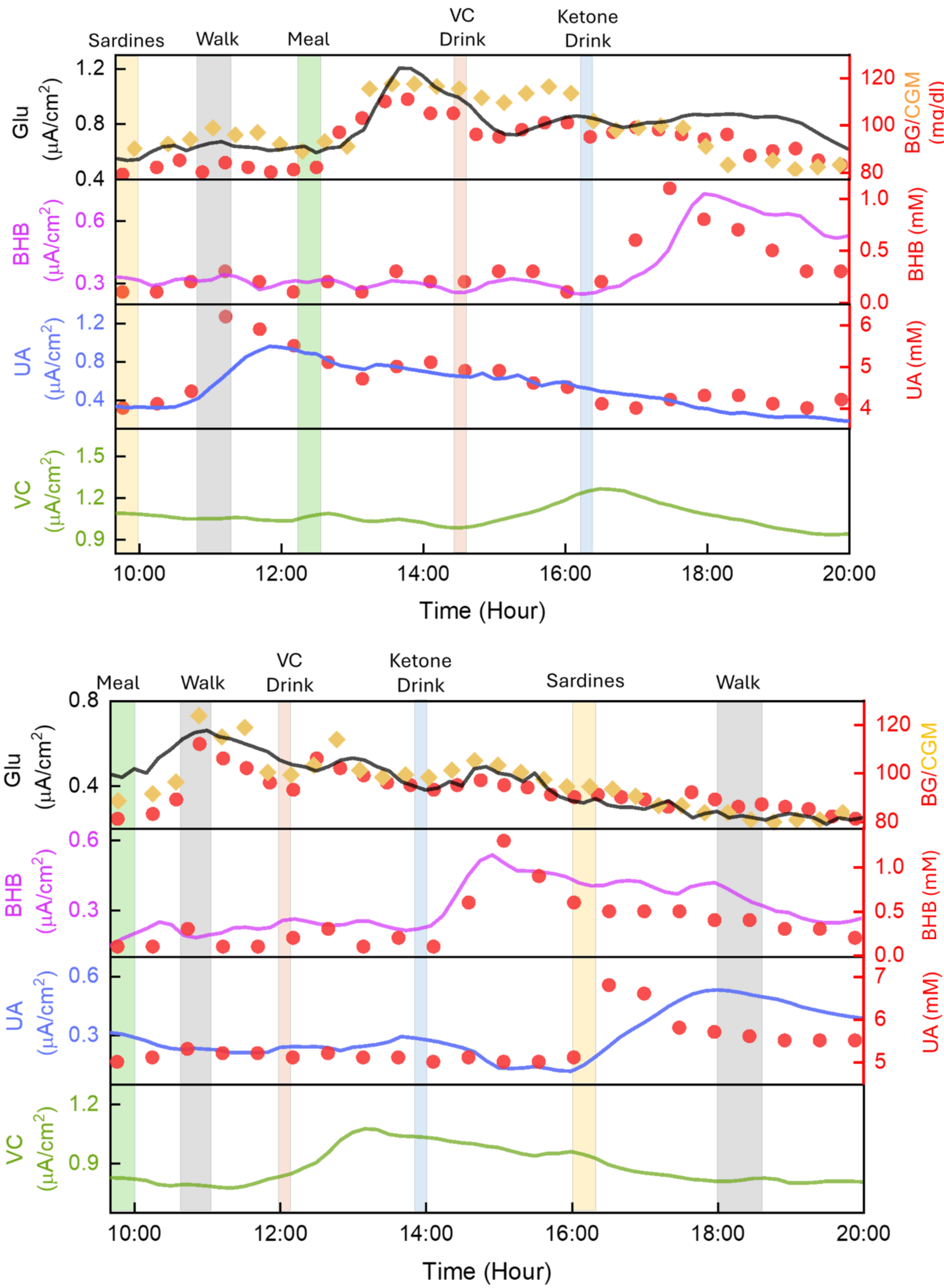

Fig. S51: Current profiles of glucose, AA, UA and BHB from T1D subject related to Fig.6a.

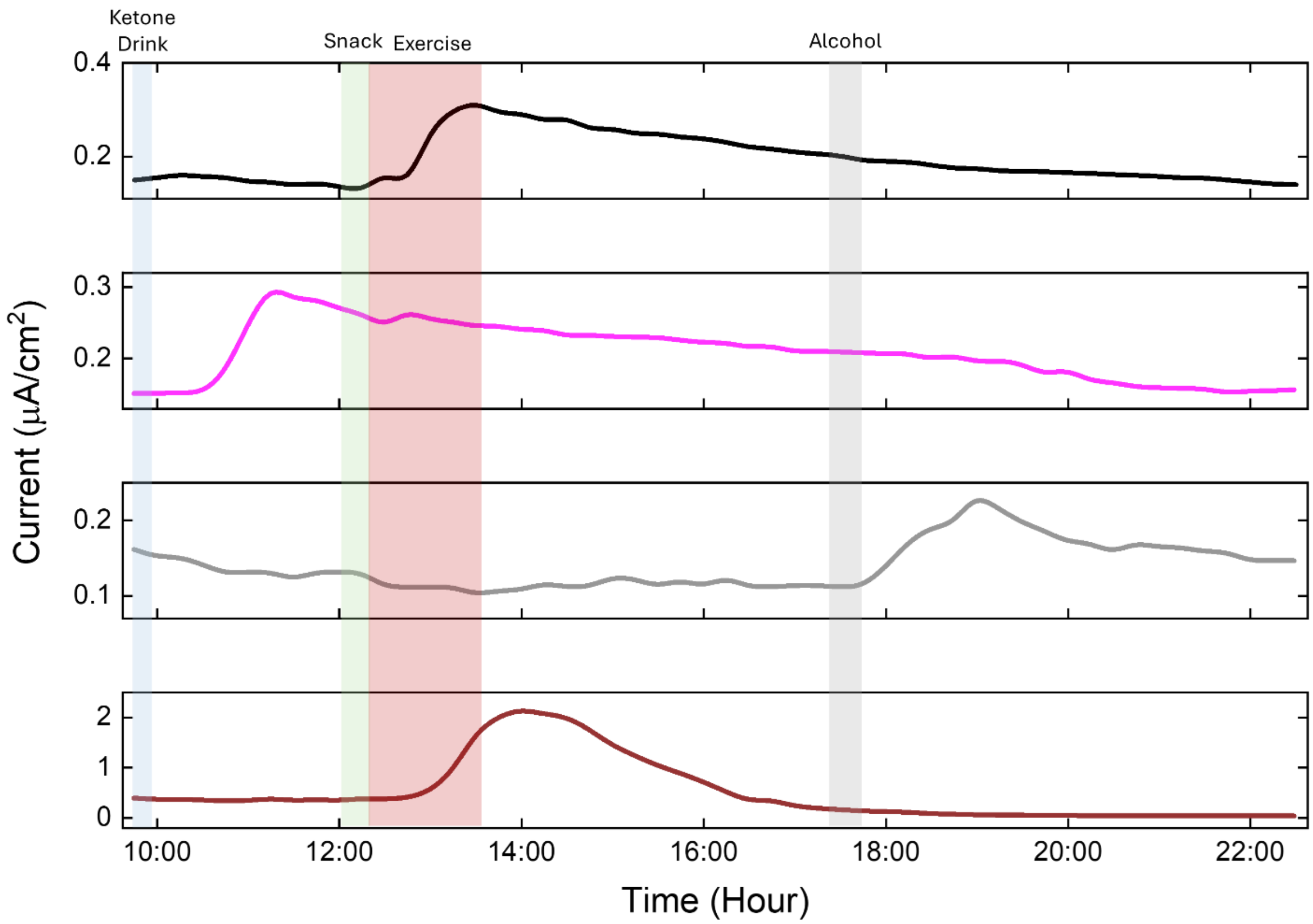

Fig. S52: Current profiles of glucose, BHB, alcohol and lactate from the healthy subject related to Fig. 6a.

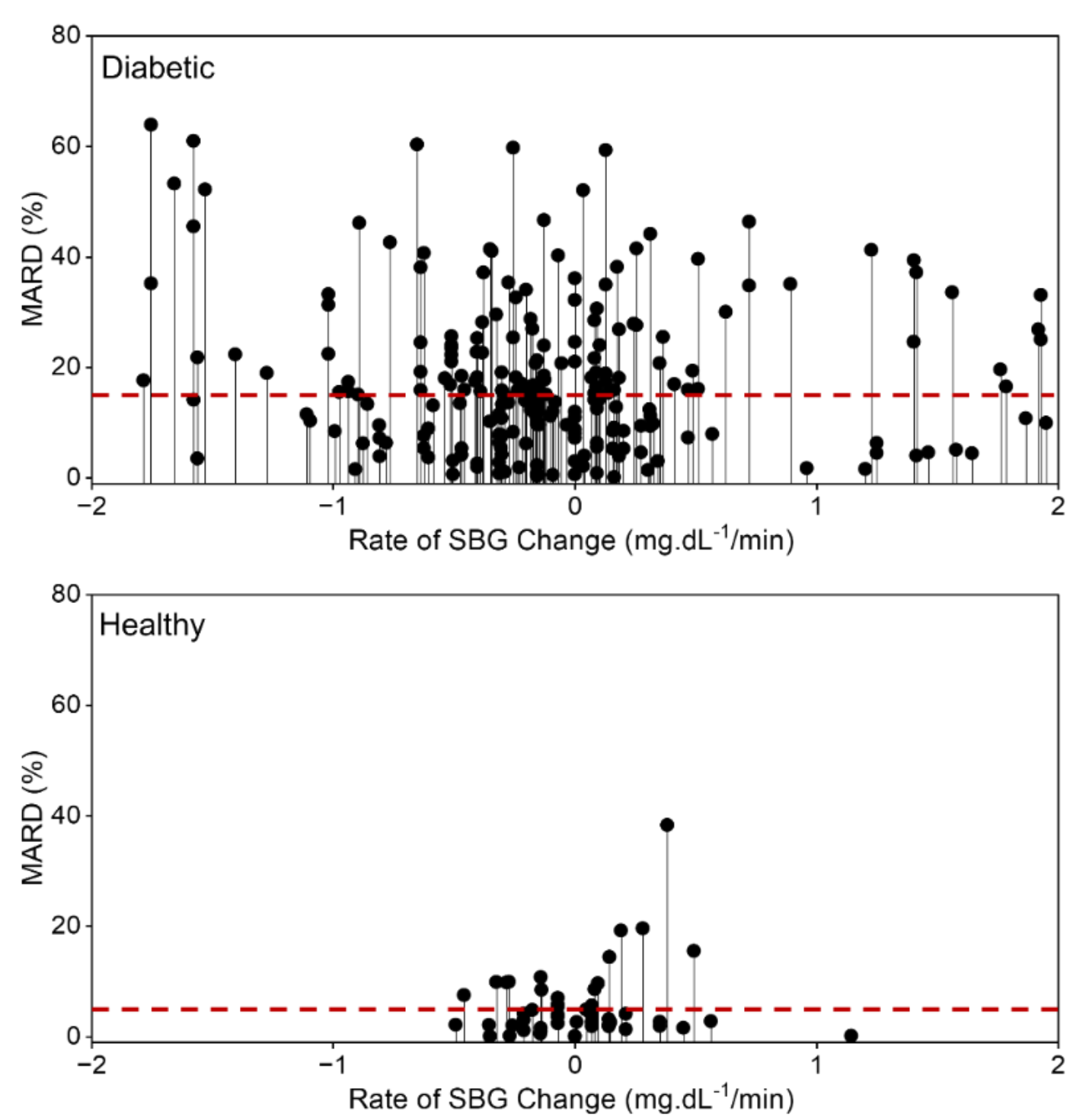

Fig. S53: Comparison of MARD vs. Rate of $SBG\ (t)$ change. For T1-D, majority of points with the lowest MARD lie when SBG change rate ranges from -1 to +0.5 $mg.dL^{-1}/min$. Higher MARD occurs under -1 to -2 $mg.dL^{-1}/min$ and +1 to +2 $mg.dL^{-1}/min$ range. The average MARD (red dashed line) throughout the whole range ranges ~16.80%. For healthy, majority of points with the lowest MARD lie when SBG change rate ranges from -0.5 to +0.2 $mg.dL^{-1}/min$. Higher MARD occurs under +0.2 to +0.5 $mg.dL^{-1}/min$. The average MARD throughout the whole range ranges ~ 3.33 %.

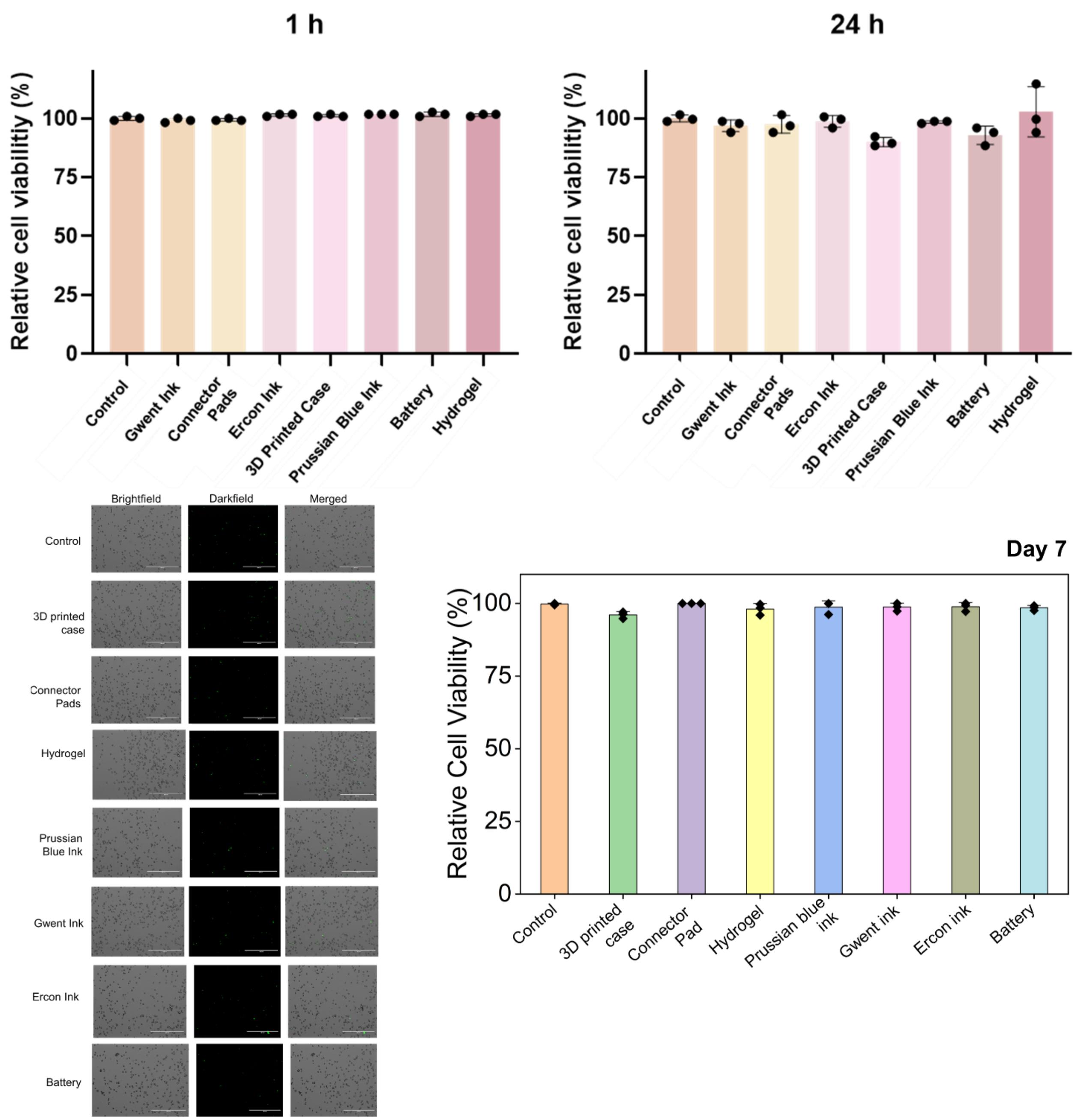

Fig. S54: Cytotoxicity test of CHARM's components with THP-1 cells. All components achieved a viability percentage above 90%, indicating fully safe to be used on skin. The THP-1 assay was intended as a preliminary general cytotoxicity/immune-compatibility screening rather than a definitive validation of skin safety. Cell viability studies are also shown till day 7, proving all components to be skin-safe for long-term usage. Bar denotes mean ± SD from n=3 data points (back dot).

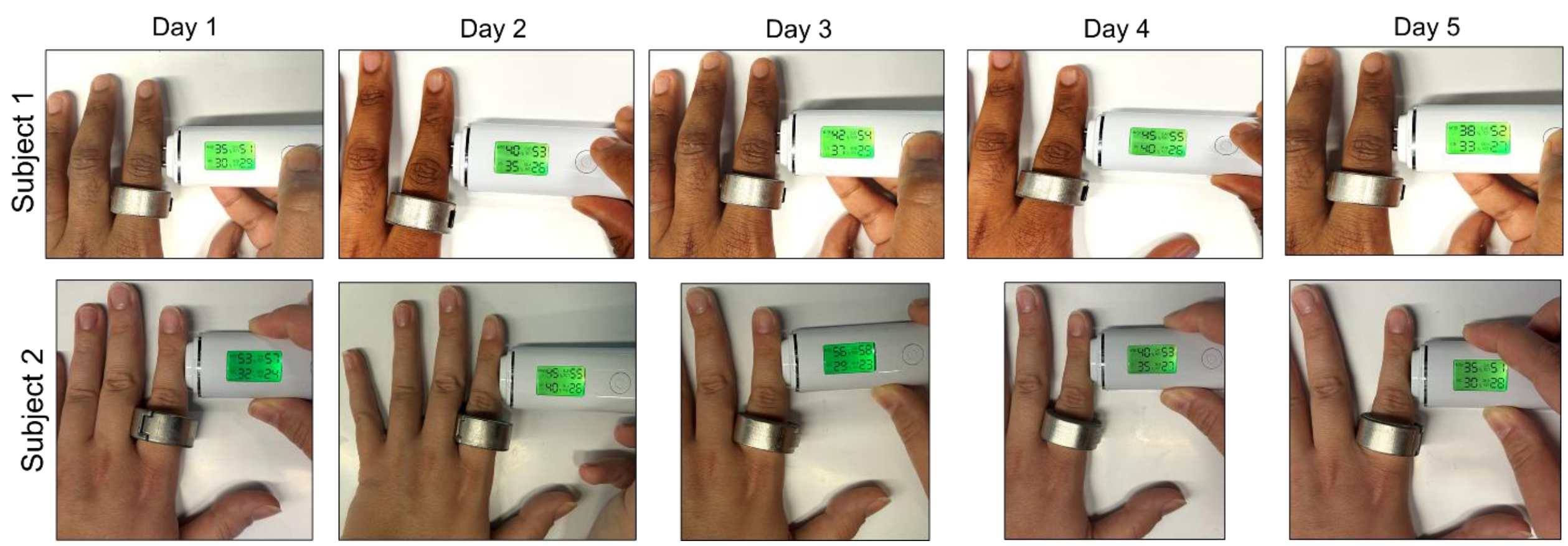

Fig. S55: Multiday on-skin tolerance test of CHARM. A commercial skin hydration testing device (EpixCare) was used. Readings in green zone for 5 days clearly justifies the long-term safety of our device. Left top: Water %, Right top: Elasticity, Left bottom: Oil %, Right bottom: Skin age.

References:

1. Tu, J. *et al.* A wireless patch for the monitoring of C-reactive protein in sweat. *Nat. Biomed. Eng.* **7**, 1293–1306 (2023).
2. Ye, C. *et al.* A wearable aptamer nanobiosensor for non-invasive female hormone monitoring. *Nat. Nanotechnol.* **19**, 330–337 (2024).
3. Fu, J. *et al.* Wearable ring sensor for monitoring biomarkers of atherosclerosis in sweat. *Talanta* **287**, 127608 (2025).
4. Razmjou, A., Liu, Q., Simon, G. P. & Wang, H. Bifunctional Polymer Hydrogel Layers As Forward Osmosis Draw Agents for Continuous Production of Fresh Water Using Solar Energy. *Environ. Sci. Technol.* **47**, 13160–13166 (2013).
5. Horkay, F., Tasaki, I. & Basser, P. J. Osmotic Swelling of Polyacrylate Hydrogels in Physiological Salt Solutions. *Biomacromolecules* **1**, 84–90 (2000).
6. Horkay, F. & Basser, P. J. Osmotic observations on chemically cross-linked DNA gels in physiological salt solutions. *Biomacromolecules* **5**, 232–237 (2004).
7. Baker, J. P., Hong, L. H., Blanch, H. W. & Prausnitz, J. M. Effect of Initial Total Monomer Concentration on the Swelling Behavior of Cationic Acrylamide-Based Hydrogels. *Macromolecules* **27**, 1446–1454 (1994).
8. Durmaz, S. & Okay, O. Acrylamide/2-acrylamido-2-methylpropane sulfonic acid sodium salt-based hydrogels: synthesis and characterization. *Polymer (Guildf).* **41**, 3693–3704 (2000).
9. Morales, D., Palleau, E., Dickey, M. D. & Velev, O. D. Electro-actuated hydrogel walkers with dual responsive legs. *Soft Matter* **10**, 1337–1348 (2014).
10. Li, X., Qin, H., Zhang, X. & Guo, Z. Triple-network hydrogels with high strength, low friction and self-healing by chemical-physical crosslinking. *J. Colloid Interface Sci.* **556**, 549–556 (2019).
11. Lin, M. *et al.* A fully integrated wearable ultrasound system to monitor deep tissues in moving subjects. *Nat. Biotechnol.* **42**, 448–457 (2024).
12. Katsamanis, F. & Raftopoulos, D. D. Determination of mechanical properties of human femoral cortical bone by the Hopkinson bar stress technique. *J. Biomech.* **23**, 1173–1184 (1990).
13. Schaumburg, F., Kler, P. A. & Berli, C. L. A. Numerical prototyping of lateral flow biosensors. *Sensors Actuators, B Chem.* **259**, 1099–1107 (2018).
14. Parvazinia, M., Nassehi, V., Wakeman, R. J. & Ghoreishy, M. H. R. Finite element modelling of flow through a porous medium between two parallel plates using the Brinkman equation. *Transp. Porous Media* **63**, 71–90 (2006).
15. Dickinson, E. J. F. & Wain, A. J. The Butler-Volmer equation in electrochemical theory: Origins, value, and practical application. *J. Electroanal. Chem.* **872**, 114145 (2020).
