# Supplementary material for: A fully integrated smart ring for daily biochemical monitoring
Source: Nat Commun. 2026 Jul 23;17:9003. doi: 10.1038/s41467-026-75980-z (PMC13507133; doi:10.1038/s41467-026-75980-z)
Supplement: Supplementary file 2 [file 41467_2026_75980_MOESM2_ESM.pdf]

Description of Additional Supplementary Files

Supplementary video -1. Real-time biomarker profile recording on phone with CHARM

Supplementary video – 2. von Mises Stress at hydrogel-finger interface
