# Supplementary material for: A fully integrated smart ring for daily biochemical monitoring
Source: Nat Commun. 2026 Jul 23;17:9003. doi: 10.1038/s41467-026-75980-z (PMC13507133; doi:10.1038/s41467-026-75980-z)
Supplement: Supplementary file 3 [file 41467_2026_75980_MOESM5_ESM.pdf]

nature portfolio

Corresponding author(s): Joseph Wang

Last updated by author(s): Jun 18, 2026

Reporting Summary

Nature Portfolio wishes to improve the reproducibility of the work that we publish. This form provides structure for consistency and transparency in reporting. For further information on Nature Portfolio policies, see our Editorial Policies and the Editorial Policy Checklist.

Statistics

For all statistical analyses, confirm that the following items are present in the figure legend, table legend, main text, or Methods section.

n/a	Confirmed	
☐	☒	The exact sample size (*n*) for each experimental group/condition, given as a discrete number and unit of measurement
☐	☒	A statement on whether measurements were taken from distinct samples or whether the same sample was measured repeatedly
☐	☒	The statistical test(s) used AND whether they are one- or two-sided<br>*Only common tests should be described solely by name; describe more complex techniques in the Methods section.*
☒	☐	A description of all covariates tested
☐	☒	A description of any assumptions or corrections, such as tests of normality and adjustment for multiple comparisons
☐	☒	A full description of the statistical parameters including central tendency (e.g. means) or other basic estimates (e.g. regression coefficient) AND variation (e.g. standard deviation) or associated estimates of uncertainty (e.g. confidence intervals)
☐	☒	For null hypothesis testing, the test statistic (e.g. *F*, *t*, *r*) with confidence intervals, effect sizes, degrees of freedom and *P* value noted<br>*Give P values as exact values whenever suitable.*
☒	☐	For Bayesian analysis, information on the choice of priors and Markov chain Monte Carlo settings
☒	☐	For hierarchical and complex designs, identification of the appropriate level for tests and full reporting of outcomes
☐	☒	Estimates of effect sizes (e.g. Cohen's *d*, Pearson's *r*), indicating how they were calculated

Our web collection on statistics for biologists contains articles on many of the points above.

Software and code

Policy information about availability of computer code

Data collection	Electrochemical data were collected using PalmSens4, EmStat3 and Autolab (PGSTAT101), and with the printed circuit board shown in the mansucript. Landt Instruments Battery Test Equipment was used for battery data collection. Initial circuit development and validation used the AD5940-EVAL (Analog Devices) and the Arduino NANO 33 BLE Rev2 (Arduino). The final custom circuit design used the NRF52840 Evaluation board (Nordic Semiconductor). The phone app was developed using node.js. All photos were taken using Nikon D3400 camera.
Data analysis	Software used in analysis include PSTrace 5.10, Nova 2.1 (Metrohm), ImageJ, Origin 2026, BLE Scanner, Arduino IDE 2.3.10, MATLAB R2024b, NRF Connect Desktop, and Microsoft Excel

For manuscripts utilizing custom algorithms or software that are central to the research but not yet described in published literature, software must be made available to editors and reviewers. We strongly encourage code deposition in a community repository (e.g. GitHub). See the Nature Portfolio guidelines for submitting code & software for further information.

Data

Policy information about availability of data

All manuscripts must include a data availability statement. This statement should provide the following information, where applicable:

- Accession codes, unique identifiers, or web links for publicly available datasets
- A description of any restrictions on data availability
- For clinical datasets or third party data, please ensure that the statement adheres to our policy

All data supporting the findings of this study are available within the article and its supplementary files. Any additional requests for information can be directed to and will be fulfilled by the corresponding authors. Source data are provided in this paper. All data needed to evaluate the conclusions in the paper are present in the paper or the Supplementary Information. Source data are provided in this paper.

Research involving human participants, their data, or biological material

Policy information about studies with human participants or human data. See also policy information about sex, gender (identity/presentation), and sexual orientation and race, ethnicity and racism.

Reporting on sex and gender	All in-vitro dye-based flow rate studies were performed on 4 subjects (2 male, 2 female)<br>Healthy on-body testing was performed on 1 male subject<br>Type 1 Diabetic on-body testing was performed on 2 subjects (1 male and 1 female)
Reporting on race, ethnicity, or other socially relevant groupings	In vitro dye tests: 2 Asian, 2 Hispanic<br>Healthy on-body testing: 1 Asian<br>Type 1 Diabetic on-body testing: 1 Asian, 1 Caucasian.
Population characteristics	Individuals with Type 1 diabetes have lived with the condition since they were young.
Recruitment	Recruitment of participants were conducted through word of mouth.
Ethics oversight	All testing protocol were strictly followed as per approved IRB (UCSD #130003).

Note that full information on the approval of the study protocol must also be provided in the manuscript.

Field-specific reporting

Please select the one below that is the best fit for your research. If you are not sure, read the appropriate sections before making your selection.

☒ Life sciences ☐ Behavioural & social sciences ☐ Ecological, evolutionary & environmental sciences

For a reference copy of the document with all sections, see nature.com/documents/nr-reporting-summary-flat.pdf

Life sciences study design

All studies must disclose on these points even when the disclosure is negative.

Sample size	All on-body studies were conducted on 7 subjects (healthy and Type 1 diabetic). No statistical analysis was used to determine total subject number as this is a purely exploratory proof-of-concept study. Sample sizes were selected based on prior comparable studies and were sufficient to assess measurement feasibility, reproducibility, and trends across repeated measurements.
Data exclusions	No data were excluded.
Replication	Experiments were repeated at least three-four times and experimental results were reproducible.
Randomization	Allocation of both healthy and type 1 diabetic subjects were randomized.
Blinding	Investigators were not blinded to group allocation during data collection and analysis. Blinding was not feasible as the study involved objective sweat biomarker measurements under predefined conditions. Potential bias was minimized using standardized protocols and predefined data-analysis procedures.

Reporting for specific materials, systems and methods

We require information from authors about some types of materials, experimental systems and methods used in many studies. Here, indicate whether each material, system or method listed is relevant to your study. If you are not sure if a list item applies to your research, read the appropriate section before selecting a response.

Materials & experimental systems

n/a	Involved in the study
☒	☐ Antibodies
☐	☒ Eukaryotic cell lines
☒	☐ Palaeontology and archaeology
☒	☐ Animals and other organisms
☒	☐ Clinical data
☒	☐ Dual use research of concern
☒	☐ Plants

Methods

n/a	Involved in the study
☒	☐ ChIP-seq
☒	☐ Flow cytometry
☒	☐ MRI-based neuroimaging

Eukaryotic cell lines

Policy information about cell lines and Sex and Gender in Research

Cell line source(s)	Human THP-1 monocytic cells were obtained from the American Type Culture Collection (ATCC, TIB-202), isolated from the peripheral blood of a male acute monocytic leukemia patient.
Authentication	THP-1 cells were obtained from a commercial cell bank and used without further genetic modification. No additional authentication was performed in-house.
Mycoplasma contamination	THP-1 cells were routinely tested and confirmed negative for mycoplasma contamination.
Commonly misidentified lines (See ICLAC register)	THP-1 cells were checked against the ICLAC Register of Misidentified Cell Lines, and no commonly misidentified cell lines were used in this study.

Plants

Seed stocks	N/A
Novel plant genotypes	N/A
Authentication	N/A
