# Supplementary material for: A fully integrated smart ring for daily biochemical monitoring
Source: Nat Commun. 2026 Jul 23;17:9003. doi: 10.1038/s41467-026-75980-z (PMC13507133; doi:10.1038/s41467-026-75980-z)
Supplement: Supplementary file 4 [file 41467_2026_75980_MOESM6_ESM.pdf]

nature portfolio

# Peer Review File

# A Fully Integrated Smart Ring for Daily Biochemical Monitoring

Corresponding Author: Professor Joseph Wang

**This file contains all reviewer reports in order by version, followed by all author rebuttals in order by version.**

Version 0:

Reviewer comments:

Reviewer #1

(Remarks to the Author)
This manuscript presents a significant advancement in wearable biochemical sensing by introducing CHARM, a fully integrated smart ring capable of continuous, real-time monitoring of multiple biomarkers from sweat. The device combines osmotic sweat extraction, flexible Zn-AgO batteries, miniaturized multichannel electronics, and electrochemical sensor arrays into a compact ring form factor. The validation in both healthy and type 1 diabetic subjects is particularly commendable. Below are several points that require clarification or additional data to enhance the manuscript's rigor.

1. In Figure 2f, while absolute signal changes at low flow rates appear small, the rate of change for glucose, lactate, and uric acid seems noticeable. Please clarify how flow rate interference was accounted for in the sensor readings.
2. In Figure 2g, the flux of each marker declines after reaching its peak. Please provide explanation for this behaviour.
3. The FPCB system uses sleep mode to conserve power. To ensure reliable real-time monitoring, could you demonstrate its Bluetooth automatic reconnection capability after waking from sleep?
4. Does the FPCB operate with multiple channels simultaneously? If so, it would be good to know whether crosstalk between channels has been evaluated.
5. In Figure 3d, a 10 MΩ shunt resistor is used for temporal multiplexing. It would strengthen the claim if you could provide quantitative comparisons showing that baseline drift, carryover, and sensitivity are indeed well controlled.
6. The two-point mapping from sweat current to SBG is highly empirical and needs stronger justification (e.g., under variable sweat rate) and a fully reproducible calibration protocol. The authors should also state whether calibration and evaluation days were strictly separated to avoid data leakage.
7. There should be a delay between sweat and blood measurements. It would be useful to see how this was handled—whether a correction method was applied or if the delay's impact on monitoring accuracy was assessed.
8. The data in Figures 5 and 6 appear to come from only one type 1 diabetic participant, though N = 355 measurements is reported. Including data from at least 3 different patients would significantly strengthen the clinical generalizability of the findings.
9. In Figure 6, the parameter a = 0.02 ± 0.006 shows a 30% variation. Could you clarify whether this variation is due to sensor differences or individual differences? Also, is there a significant difference in a between diabetic and non-diabetic participants? It would be good to demonstrate that, under the same a value, CHARM can distinguish between these groups.
10. The battery is a key innovation, but many key metrics (cycle life, charge-discharge curves, dendrite formation) are shown for n = 1 only. It would be helpful to include reproducibility data from at least three batteries. Currently, commercial batteries easily reach thousands of cycles or more. After multiple charge-discharge cycles of the proposed battery, will dendrites grow on the electrode? Please provide SEM images. Additionally, what safety measures are in place for overcharge/over-discharge conditions given the battery's small volume?
11. The authors claim that coefficients a and b remain stable up to day 60. It would be helpful to see day-by-day trajectories and clarify whether sensor replacement or recalibration occurred during this period. Given the ~10-hour consumable lifetime implied by the microfluidic design, validating a and b stability across different patch batches would also be valuable.

Reviewer #2

(Remarks to the Author)

The paper presents a system-level integration of biochemical sweat sensors into a ring comprising a printed battery, a flexible fluidic interface, and off-the-shelf electronics for sensor sampling and wireless connectivity. The application is certainly significant, and the complete system shows that biochemical monitoring can be achieved in a popular formfactor of smart rings. The in-vivo results and the long-term nutritional analysis show that the prototype has sufficient robustness for extended testing with reliable sensor data collection and readout. The osmotic perspiration approach improves the sweat collection, with detailed characterisation of each analyte sensor.

Overall, the work is commendable, with the wearable integration of multiple electrochemical sensors and their readout not being trivial. The motivation that electronics in a commercial ring are bulky needs to be further strengthened. In fact, electronics miniaturisation, for example through a system-on-chip (SoC), can reduce the complexity of the readout and communication circuitry. The use of flexible PCBs is certainly common in rigid-flex interconnects across consumer devices, such as between PCBs in a phone.

Please consider the following comments to be addressed:

- The key differentiators between the proposed system and other flexible electronics-based microfluidic patches should be explained more clearly. The improvement at a component level, in e.g. reliability and long-term stability, compared to a disposable patch, could be strengthened. Ideally, a table in the supplementary material could compare the ring-integrated system to patches such as:

Tu, J., Min, J., Song, Y. et al. A wireless patch for the monitoring of C-reactive protein in sweat. Nat. Biomed. Eng 7, 1293–1306 (2023). https://doi.org/10.1038/s41551-023-01059-5

- The batteries are highlighted as a key element of the work. The need for a printable and flexible battery could be made clearer. In particular, a capacity of 10 mAh can be achieved using off-the-shelf microbatteries (for example Seiko SII Micro Battery), which could be packaged within the final ring form factor. In such cases, the bendability could be achieved using the substrate and interconnects, similar to how the rigid ICs are integrated.

- While the battery is rechargeable and demonstrated over multiple cycles, the full system does not include a charging circuit. If this cannot be included, a discussion of the space constraints required for inductive charging, or through an additional contact in part of the exposed circuitry, should be discussed. There are several commercially available waterproof connectors which could be incorporated.

- The resistance of the prototype to wetness needs to be investigated. Commercial smart rings are waterproof (IP68 for Oura). To allow the sensing mechanism, the ring will not be easily encapsulated using existing approaches for water proofing by sealing the entire ring. Due to the fluidic sensing function, this ring is unlikely to require submersion, but might experience washing or spillages during the day.
The impact on both the fluidic channels and the PCBs should be discussed and experimentally evaluated.

- The RSSI shows the Bluetooth operation up to 3 m. This is a limited range where BLE connections can typically be maintained to 10s of metres. There is likely a strong impact of human body shadowing and detuning in the antenna, which need to be investigated further. For example, the line-of-sight vs non-line-of-sight scenarios, in particular, depending on the position of the finger relative to the hand (e.g. in a closed grip), or the data being transmitted through the body. The transmitted power level should also be included.
Ideally, the antenna properties related to this query should be characterised in more depth, though the RSSI level functional tests could suffice, once performed in the LoS/NLoS arrangements to show the performance limit. Chip antennas are highly dependent on device size, which the ring likely does not meet.

- Please clarify the data transmission details, for example, through firmware flow chart, explaining how the data is encoded, the time needed to transfer the packers, and also if any data processing takes place on the MCU itself, or if it is entirely done on the host PC or smartphone. Both an app and a PC are discussed in the manuscript or video; it will be good to discuss any challenges of using an app.

Minor corrections:

- Both the MCU and the AFE could operate around 3.6-3.8; it would be good to see the motiavtion for the buck converter and if it could potentially be removed.

- The ceramic filter mentioned does not perform impedance matching, as the antenna should be directly matched.

- For the IR pictures, please include an optical photograph to map the position of the device to the hand. The baseline temperature appears to be low for an on-skin circuit.

- Typo: "serail-wire"

Reviewer #3

(Remarks to the Author)
The manuscript presents an integrated wearable ring platform (CHARM) for multiplexed electrochemical enzymatic sensors in passive sweat captured and transported using hydrogel and paper microfluidic assembly driven by osmotic sweat extraction integrated with wireless electronics, powered by a flexible AgO-Zn battery. While the ring form factor represents an impressive engineering integration and is attractive from a translational standpoint given the growing adoption of smart rings for health monitoring, several aspects of the study raise significant concerns. In particular, the limited size of the human cohort, insufficient calibration and characterization of the microfluidic transport system, and incomplete evaluation of long-term sensor stability weaken the strength of the conclusions.
Additionally, the core sensing strategy and the hydrogel-mediated osmotic sweat extraction mechanism have been previously established by the authors in earlier work (see Comment 3). As such, the present manuscript primarily represents an integration of these previously reported elements into a ring-based platform with multiplexing, onboard electronics, and flexible power delivery. Addressing these concerns would significantly strengthen the manuscript and improve its suitability for publication.

Specific Comments:
1. The human validation cohort is extremely limited relative to the breadth of the manuscript's claims. The text refers broadly to testing in "one healthy and one type 1 diabetic individual," and the figures and supplementary information appear to confirm that the in vivo data are derived from repeated measurements from only two identifiable subjects: a single healthy participant and a single T1D participant. Repeated measurements from the same individuals cannot substitute for subject-level biological replication, particularly when the manuscript aims to demonstrate continuous monitoring of multiple chemical biomarkers in real time for at-risk populations.
2. In vitro experiments show 6 analytes whereas the on body tests only mention 4, excluding ascorbic acid (AA) and uric acid (UA). Please elaborate.
3. As a result, the applicability of the platform to diabetes management and diabetic ketoacidosis risk assessment is insufficiently supported by the presented data. The current dataset does not evaluate device performance across a meaningful glycaemic range, across multiple diabetic phenotypes, or under clinically relevant physiological conditions such as hypoglycaemia, rapid glycaemic excursions, or dehydration. Additional validation across a larger and more diverse cohort would be necessary to support the manuscript's translational claims.
4. The core sensing concept and hydrogel-mediated extraction strategy were already established in the authors' earlier work. Please see below:
a. Saha, Tamoghna, et al. "A passive perspiration inspired wearable platform for continuous glucose monitoring." Advanced Science 11.41 (2024): 2405518.
b. Yin, Lu, et al. "High performance printed AgO-Zn rechargeable battery for flexible electronics." Joule 5.1 (2021): 228-248.
5. The manuscript supports the ring site as a practical and wearable location with sufficient sweat access, but it does not establish that this site is biologically superior for biochemical sensing. Although the manuscript argues that the lower sweat gland density at the ring site leads to a reduced sweat flux that may prolong sampling by avoiding rapid channel saturation, it does not show that the ring site is inherently a better location for biochemical sensing. At present, the ring site appears to be a practical compromise rather than a biologically superior sensing site, and the authors should articulate this more clearly.
6. It is unclear what the maximum sweat storage capacity of the hydrogel component is. Similarly, the manuscript does not specify the total volume capacity of the combined hydrogel paper microfluidic assembly used for sweat collection and transport. In particular, it remains unclear how the system prevents previously absorbed ("old") sweat from mixing with newly generated sweat, which could potentially skew the real-time biochemical readouts.
7. Was the flow capacity of the hydrogel evaluated under varying environmental conditions such as relative humidity (RH) and temperature? It is also unclear whether swelling or drying behavior during conditions of elevated sweat production such as physical exertion, hot showers, or sauna-like environments was considered or simulated prior to the in vivo experiments. Clarifying whether these factors were assessed would help establish the robustness of the ring in practical use.
8. The manuscript uses 150 nL/min as both an experimentally inferred ring-site flow regime and a design/characterization condition, but does not sufficiently justify how representative this is across users and environments? Sweat rates vary substantially among individuals and across environmental conditions, and additional validation across a wider range of sweat production rates, including very low perspiration and higher flow regimes, would strengthen confidence in the sensing platform's robustness.
9. While the sensing region is positioned ~1.3 cm from the hydrogel inlet and the estimated analyte lag time is only ~2–3 minutes, the manuscript also claims ~10 hours of operation. In this context, it is unclear how the authors address potential drying of gel/evaporation of sweat during the extended useon-body, in changing ambient conditions, and intermittent real-world wear?

.
10. The manuscript describes the use of personalized calibration parameters and suggests that recalibration may be required if deviations occur. However, it is unclear how frequently users would detect such calibration drift in practice. While the authors report enzymatic sensor stability for up to 9 days, the manuscript claims that the calibration based on the current change ($\Delta I$) remains stable for up to 60 days. It is not clear whether the sensors are stable for 60 days and the manuscript does not sufficiently address how batch-to-batch sensor variability is managed when calibrating the CHARM platform?.
11. The cytotoxicity assessment is not well aligned with the intended exposure site of the ring. The biocompatibility is evaluated using THP-1 cells, which are more suitable for a broad immune/cytotoxicity screen than for skin-contact validation. For a wearable intended for prolonged skin interfacing, the authors should use skin-relevant models.
12. Also, it is recommended that the authors include residual PAAm monomer and EG leaching assessment, dermatology testing studies, and multi-day on-skin tolerance data. The current THP-1 assay is insufficient to support the statement that the components are "fully safe to be used on skin."

13. The manuscript does not clearly discuss battery depletion, disposal, or replacement strategies. It would be helpful to clarify whether the device is intended to be disposable, rechargeable, or modular, and whether components such as the battery or sensing elements can be replaced.

14. The manuscript does not clearly address the storage shelf life of the enzyme-based sensing module. It is unclear whether the reported 60-day stability refers to the operational stability of the complete device (hydrogel, microfluidics, enzymatic sensors, and electronics) or only to the storage stability of individual components. In either case, the authors should specify the storage conditions required to maintain hydrogel functionality, enzyme activity, and battery performance, including potential self-discharge. The authors should also clarify whether the reported long-term stability reflects pre-use shelf life, on-body operational stability, or short-term benchtop sensor performance.

15. The nomenclature for the analytes should be maintained consistent throughout the manuscript. (for example, Ascorbic Acid and Vitamin C)

Version 1:

Reviewer comments:

Reviewer #1

(Remarks to the Author)
The reviewer appreciates the authors' effort to address the comments, but the previous comment on calibration remains inadequately addressed or explained.

With the introduction of both slope (a) and intercept (b), the sensor readings no longer directly reflect absolute sweat chemical concentrations nor the magnitude of dynamic changes. Without multiple calibrations against a standardized device (e.g., a blood glucose meter), it is unclear how users can obtain accurate readings.

Across Figures S36–S50, the values of a and b vary considerably for all sensors. Even within a single subject, these calibration parameters show significant variation — for example, the pronounced variation in b-value in Figure S50. Without recalibration against a standard device each time to obtain updated a and b values, the resulting concentration errors would likely be substantial. The authors attribute this variation to individual differences but provide no data excluding sensor variability. Does sensor variability have no influence on the final readings? If users perform multiple calibrations, regardless of a and b variation, can accurate readings still be guaranteed? How would users determine which a or b value to use in daily practice?

The reviewer requests that the authors add a clear calibration methodology and explanation to the manuscript to guide readers in fully understanding the procedure. Please highlight the changes; otherwise, it is extremely difficult to locate them when none are marked, especially when mixed with different bolded texts.

Reviewer #2

(Remarks to the Author)
Thank you for addressing the comments in detail and providing the requested additional experimental data. I believe the work is now suitable for publication. Please also make the following minor correction, related to the BLE performance:

The measured communication range and RSSI show that the antenna gain is extremely low (below -25 dBi without obstruction). This is likely due to the limitations of a chip antenna on a very constrained layout, while typically requiring large devices.

In Line 294, the manuscript reads:
"The RF path includes an integrated ceramic low-pass filter for harmonic suppression and out-of-band noise filtering, complemented by a compact ceramic antenna for efficient transmission"

I recommend that the phrase "efficient transmission" is removed, as the antenna/RF front end has higher losses than typical (which understandably does not hinder the application).

Reviewer #3

(Remarks to the Author)
The authors have addressed all my concerns and the manuscript can be accepted in the present form.

Version 2:

Reviewer comments:

Reviewer #1

(Remarks to the Author)
In this case, I support its publication.

**Open Access** This Peer Review File is licensed under a Creative Commons Attribution 4.0 International License, which permits use, sharing, adaptation, distribution and reproduction in any medium or format, as long as you give appropriate credit to the original author(s) and the source, provide a link to the Creative Commons license, and indicate if changes were made.
In cases where reviewers are anonymous, credit should be given to 'Anonymous Referee' and the source.
The images or other third party material in this Peer Review File are included in the article's Creative Commons license, unless indicated otherwise in a credit line to the material. If material is not included in the article's Creative Commons license and your intended use is not permitted by statutory regulation or exceeds the permitted use, you will need to obtain permission directly from the copyright holder.
To view a copy of this license, visit https://creativecommons.org/licenses/by/4.0/

## Responses to Reviewer Comments

**Reviewer #1:** *This manuscript presents a significant advancement in wearable biochemical sensing by introducing CHARM, a fully integrated smart ring capable of continuous, real-time monitoring of multiple biomarkers from sweat. The device combines osmotic sweat extraction, flexible Zn-AgO batteries, miniaturized multichannel electronics, and electrochemical sensor arrays into a compact ring form factor. The validation in both healthy and type 1 diabetic subjects is particularly commendable. Below are several points that require clarification or additional data to enhance the manuscript's rigor.*

**General Response:** We thank the Reviewer for considering our work commendable and as a significant advancement in wearable biosensing. Please find the responses for your comments below.

*1. In Figure 2f, while absolute signal changes at low flow rates appear small, the rate of change for glucose, lactate, and uric acid seems noticeable. Please clarify how flow rate interference was accounted for in the sensor readings.*

**Response:** We thank the Reviewer for this question. While we agree that signal changes for glucose, lactate and uric acid seem noticeable between 50-150 nl/min, the net change in this zone ranges ~0.09, 1.1, and 0.1 $\mu A/cm^2$ for glucose, lactate, and uric acid, respectively. The magnitude of this change is notably lower compared to when the flow rate exceeds 500 nL/min, under which these values range ~0.9, 5, and 0.6 $\mu A/cm^2$ for glucose, lactate, and uric acid, respectively. Existing standard deviations also verify the accuracy of our results.

We would also like to respectfully point out that the effect of flow rate on the sensor response is already demonstrated in **Fig. 2h** in the initial submission, where negligible changes were observed in the sensor response from the ring location of the finger. Overall, our results suggest that sweat withdrawn via osmosis tends to minimize the sweat rate effects on the sensor response.

*2. In Figure 2g, the flux of each marker declines after reaching its peak. Please provide explanation for this behavior.*

**Response:** We thank the Reviewer for pointing this out. In our FEA flow simulations, we primarily ramp the inlet concentration from a lower (C1) to a higher value (C2) and then back to the same lower value (C1). Hence, we observe a decreasing trend in the flux profile. A sentence mentioning this has been added on **page 9** and to the caption of **Fig. S14.**

***3.*** *The FPCB system uses sleep mode to conserve power. To ensure reliable real-time monitoring, could you demonstrate its Bluetooth automatic reconnection capability after waking from sleep?*

**Response:** We thank the Reviewer for this helpful suggestion. To assess Bluetooth automatic reconnection after wake-from-sleep, we conducted an additional repeated sleep–wake experiment while simultaneously recording the power-consumption trace and receiver-side serial communication log over 11 consecutive cycles. For this test, the four sensing channels were replaced with 10 MΩ resistive loads under a 500 mV excitation, and an accelerated timing protocol was used in which each channel was sequentially measured for 5 s, followed by data transmission and a 1 min sleep period. This accelerated protocol was used solely to enable repeated reconnection testing within a practical timeframe; the Bluetooth wake-up and reconnection procedure remains unchanged from the normal operating mode in the manuscript. In every cycle, the power profile verified wake-up from sleep and return to active operation, while the receiver log confirmed automatic rediscovery, reconnection, successful TX notification subscription, and resumed decoded data reception without user intervention. Together, these results demonstrate reliable automatic Bluetooth reconnection and restoration of real-time wireless monitoring after sleep. The results have been added to **Fig. S21** and a related discussion is added to **page 11**.

4. *Does the FPCB operate with multiple channels simultaneously? If so, it would be good to know whether crosstalk between channels has been evaluated.*

**Response:** We thank the Reviewer for raising this valuable question. We agree the crosstalk between channels to be a valid concern. However, our F-PCB channels operate sequentially to detect the analytes. Scan on each channel is conducted for 60 seconds. The combined sequential operation of all four channels are shown in **Fig. S23** and **Fig. S24** for peroxide and multiple biomarkers (glucose, VC, UA, and BHB) sensing, respectively. We would also like to respectfully state that the sequential functioning of our FPCB was already mentioned on **page 11** of the original submission.

*5. In Figure 3d, a 10 MΩ shunt resistor is used for temporal multiplexing. It would strengthen the claim if you could provide quantitative comparisons showing that baseline drift, carryover, and sensitivity are indeed well controlled.*

**Response:** We thank the Reviewer for this suggestion. We conducted additional measurements by monitoring the nodal voltages (working electrode and counter electrode) across a sensing channel using an oscilloscope. The experiment was performed both with and without 10 MΩ resistors. The results show that, with the 10 MΩ resistor, the voltage across the idle sensor remains effectively at 0 V, whereas without the resistor, a non-zero voltage (~1.3 V) is observed, indicating leakage current through the

sensor. These findings demonstrate that the 10 MΩ resistor effectively stabilizes and balances the sensor voltage during idle conditions. The data has been added as supplementary **Fig. S20** in the revised version.

*6. The two-point mapping from sweat current to SBG is highly empirical and needs stronger justification (e.g., under variable sweat rate) and a fully reproducible calibration protocol. The authors should also state whether calibration and evaluation days were strictly separated to avoid data leakage.*

**Response:** We thank the Reviewer for raising this valuable concern. We would like to respectfully highlight that sweat withdrawal via osmosis has proven to minimize sweat rate fluctuations on the sensor response. This is evident from the on-body trends in Fig. 2h and also from our previous studies on fingertip glucose sensing (https://advanced.onlinelibrary.wiley.com/doi/full/10.1002/advs.202405518). As a result, our calibration model does not include a sweat rate dependency factor.

The reproducibility of the calibration model across different biomarkers is supported by the negligible variation in personalized calibration parameters (a, b) observed across independent tests conducted over multiple days (**Fig. S36,37,40-42,46-50**). This demonstrates that our model reliably captures the subject's health status under the same stimulus across repeated tests over multiple days. A statement highlighting these points has been added to **page 15** of the revised version for better clarification.

*7. There should be a delay between sweat and blood measurements. It would be useful to see how this was handled—whether a correction method was applied or if the delay's impact on monitoring accuracy was assessed*.

**Response:** We thank the Reviewer for raising this valuable question. We agree about the time delay aspect between blood and sweat peaks. We did observe this delay in all our on-body trials in **Fig. 5** and **Fig. 6**. However, we have not accounted for the time-lag correction in the prediction of $SBG(t)$, and would like to respectfully state about mentioning this already on **page 21-22** (third paragraph in the Discussion section). We also mention how we can address this issue in our system. In general, the purpose of this study was to solely evaluate the biochemical performance of our integrated ring at the ring site of the finger and examine its correlation to blood.

***8.** The data in Figures 5 and 6 appear to come from only one type 1 diabetic participant, though N = 355 measurements is reported. Including data from at least 3 different patients would significantly strengthen the clinical generalizability of the findings.*

**Response:** We thank the Reviewer for this question. Although data from only one diabetic subject was presented, we would also like to respectfully point out that these 355 measurements were collected over an extended period of 2 months, as per our calibration

protocol. In response, we tested CHARM on another diabetic subject (**Fig. S40**), whose calibration stability has been tested for ~2 weeks. Accordingly, all measurements have been added to **Fig. 6bi** in the revised version with a modified figure caption.

*9. In Figure 6, the parameter a = 0.02 ± 0.006 shows a 30% variation. Could you clarify whether this variation is due to sensor differences or individual differences? Also, is there a significant difference in the difference between diabetic and non-diabetic participants? It would be good to demonstrate that, under the same a value, CHARM can distinguish between these groups.*

**Response:** We thank the Reviewer for pointing this out. We believe that this variation arises from individual differences, as the calibration stability was validated through discrete tests conducted on three random days over a two-month period. As glucose levels are influenced by daily lifestyle, nutrition, circadian rhythm, and medications, we believe that such fluctuations can inevitably appear on sensor readings on a daily basis.

There is significant difference between the $a$ and $b$ values of healthy vs. diabetic subjects. From **Fig. S36** and **Fig. S46**, it is seen that magnitude of $a$ for healthy subject ranges ~2.8X higher than the diabetic subject. This arises because blood glucose (BG) changes are greater in individuals with diabetes, which consequently lowers *a*, as $\Delta BG$ appears in its denominator. Hence, CHARM can distinguish these two groups. A statement mentioning this has been added to **page 19** in the revised version.

***10.** The battery is a key innovation, but many key metrics (cycle life, charge-discharge curves, dendrite formation) are shown for n = 1 only. It would be helpful to include reproducibility data from at least three batteries. Currently, commercial batteries easily reach thousands of cycles or more. After multiple charge-discharge cycles of the proposed battery, will dendrites grow on the electrode? Please provide SEM images. Additionally, what safety measures are in place for overcharge/over-discharge conditions given the battery's small volume?*

**Response:** We would like to thank the Reviewer for this helpful suggestion. The cycle life and charge-discharge curve data for three batteries have been added in **Fig. S31** and **Fig. S32** in the revised supplemental version. The SEM images of the anode separator after 20 cycles for three batteries are added in **Fig. S28** and discussed in the corresponding section (on **page 13**) in the main text. Safety measures include a cut-off voltage of 1.2 V for discharging steps and 2.05 V for charging steps for single batteries. The cut-off voltages are doubled for two batteries connected in-series.

*11. The authors claim that coefficients a and b remain stable up to day 60. It would be helpful to see day-by-day trajectories and clarify whether sensor replacement or recalibration occurred during this period. Given the ~10-hour consumable lifetime implied*

*by the microfluidic design, validating a and b stability across different patch batches would also be valuable.*

**Response:** We thank the Reviewer for this useful comment. We would like to respectfully mention that as our goal was to assess the long-term stability of the calibration parameters, we conducted sporadic measurements across multiple days. The negligible differences between the parameters measured on day 1 and day 60 indicate that no recalibration was necessary during this period. Thus, we believe that measuring *a* and *b* on a day-by-day basis would not provide significant additional insights and not effectively justify the assessment of long-term stability. Moreover, we would like to respectfully point out that, since *a* and *b* were evaluated on different days using a new patch each time, inter-batch variability was inherently accounted. Negligible changes in *a* and *b* till 60 days further indicates the inter-batch variability to be an insignificant influencing factor. A statement mentioning this has been added to **page 15** in the revised version.

## Reviewer #2:

*The paper presents a system-level integration of biochemical sweat sensors into a ring comprising a printed battery, a flexible fluidic interface, and off-the-shelf electronics for sensor sampling and wireless connectivity. The application is certainly significant, and the complete system shows that biochemical monitoring can be achieved in a popular formfactor of smart rings. The in-vivo results and the long-term nutritional analysis show that the prototype has sufficient robustness for extended testing with reliable sensor data collection and readout. The osmotic perspiration approach improves the sweat collection, with detailed characterization of each analyte sensor.*

*Overall, the work is commendable, with the wearable integration of multiple electrochemical sensors and their readout not being trivial. The motivation that electronics in a commercial ring are bulky needs to be further strengthened. In fact, electronics miniaturization, for example through a system-on-chip (SoC), can reduce the complexity of the readout and communication circuitry. The use of flexible PCBs is certainly common in rigid-flex interconnects across consumer devices, such as between PCBs in a phone.*

*Please consider the following comments to be addressed:*

**General Response:** We thank the Reviewer for considering our ring device to be robust and reliable, and for considering our overall work to be significant and commendable. Please find the responses for your comments below.

**1.** *The key differentiators between the proposed system and other flexible electronics-based microfluidic patches should be explained more clearly. The improvement at a*

*component level, in e.g. reliability and long-term stability, compared to a disposable patch, could be strengthened. Ideally, a table in the supplementary material could compare the ring-integrated system to patches such as:*

*Tu, J., Min, J., Song, Y. et al. A wireless patch for the monitoring of C-reactive protein in sweat. Nat. Biomed. Eng 7, 1293–1306 (2023). https://doi.org/10.1038/s41551-023-01059-5*

**Response:** We thank the Reviewer for raising this important question. We would like to respectfully point out that as our device is an integrated ring which represents a completely different formfactor compared to traditional epidermal microfluidic patches. Key differentiators for electronics and microfluidics are thus presented here against devices tested only at the ring location. The following table presents the detailed comparison and has been added as **Table S1** in the revised supplemental information. The suggested manuscript has been cited in revised version as **Ref #28.**

| Component | Parameter | This work | vs. Tu et al., | vs. Cui et al. | vs. Fu et al. |
|---|---|---|---|---|---|
| **Form factor** | N/A | Fully integrated true ring | Flexible patch | Flexible patch wrapped at ring location | Flexible patch wrapped at ring location |
| **Microfluidics** | Material | Paper | PDMS | PDMS | PDMS |
| | Accommodating volume | ~90 µL Sensing volume: 5 µL | ~ 21 µL | ~7 µL | Not reported |
| | Size | 8 cm length 3D stackable | ~ 2 cm length – No 3D stackable | ~1.5 cm length – No 3D stackable | ~1.5 cm length – No 3D stackable |
| | Inlet | 5 mm diameter | 0.2 mm diameter | 5 mm diameter | 6 mm diameter |
| | Mixing chamber | No | 0.8 mm diameter | No | No |
| | Outlet | 7 mm diameter | 0.1 mm diameter | Not reported | 0.5 mm diameter |
| **Battery** | Form factor | Printed flexible battery | Coin cell | Coin cell | Coin cell |
| | Voltage | 3.6 V | 3.8 V | 3.8 V | 3.8 V |
| | Capacity | 10 mAh | 8 mAh | 8 mAh | 8 mAh |
| **Sensors** | Biomarker | Glucose, lactate, ketone, alcohol, uric acid, | C-reactive protein, pH, sweat rate | Estradiol, pH, sweat rate | C-reactive protein, cholesterol, |

| | | | | | |
|---|---|---|---|---|---|
| | | ascorbic acid | | | potassium, sweat rate |
| **Sweat Access** | Technique | Osmotic (passive) | Iontophoresis (active) | Iontophoresis (active) | Iontophoresis (active) |
| | Sweat rate | 0.1-0.2 µL/min | 0.5-3.5 µL/min | Not reported | Not reported |
| **Operation** | Sensing duration | ~12 hours | 60s scans | 10s discrete scans over multiple days | Discrete scans |

**References:**


a. Tu, J. *et al.* A wireless patch for the monitoring of C-reactive protein in sweat. *Nat. Biomed. Eng.* **7**, 1293–1306 (2023).
b. Ye, C. *et al.* A wearable aptamer nanobiosensor for non-invasive female hormone monitoring. *Nat. Nanotechnol.* **19**, 330–337 (2024).
c. Fu, J. *et al.* Wearable ring sensor for monitoring biomarkers of atherosclerosis in sweat. *Talanta* **287**, 127608 (2025).


**2.** *The batteries are highlighted as a key element of the work. The need for a printable and flexible battery could be made clearer. In particular, a capacity of 10 mAh can be achieved using off-the-shelf microbatteries (for example Seiko SII Micro Battery), which could be packaged within the final ring form factor. In such cases, the bendability could be achieved using the substrate and interconnects, similar to how the rigid ICs are integrated.*

**Response:** We thank the Reviewer for this valuable suggestion and agree that integrating a rigid battery with flexible interconnects could also have been a viable approach for our system. However, since CHARM's battery casing is curved to conform to the finger, we designed the battery to match this curvature, ensuring proper fit, conformability, and adaptability to varying finger sizes. Hence, we used screen printed batteries made with stretchable inks and interconnects. A sentence mentioning this has been added on **page 12** in the revised manuscript version.

**3.** *While the battery is rechargeable and demonstrated over multiple cycles, the full system does not include a charging circuit. If this cannot be included, a discussion of the space constraints required for inductive charging, or through an additional contact in part of the exposed circuitry, should be discussed. There are several commercially available waterproof connectors which could be incorporated.*

**Response:** We thank the Reviewer for this important comment. We acknowledge that the current prototype does not integrate an on-board charging circuit, as the primary objective of this work is to demonstrate the system concept rather than a fully integrated commercial device. As the Reviewer suggested, recharging could be implemented

through external electrical contacts, such as exposed pads or compact waterproof connectors, or via inductive wireless charging. These approaches introduce additional requirements for space and integration within the ring form factor and will be valuable directions for future development. This has been added to **page 22** in the revised version.

4. *The resistance of the prototype to wetness needs to be investigated. Commercial smart rings are waterproof (IP68 for Oura). To allow the sensing mechanism, the ring will not be easily encapsulated using existing approaches for water proofing by sealing the entire ring. Due to the fluidic sensing function, this ring is unlikely to require submersion but might experience washing or spillages during the day. The impact on both the fluidic channels and the PCBs should be discussed and experimentally evaluated.*

**Response:** We thank the Reviewer for raising this important aspect regarding encapsulation. We would like to respectfully point out that the fluidic channel in our device is sandwiched between two SEBS layers, thereby preventing external water interactions (**Fig. 1**). Overall, the primary goal of this work was to demonstrate that system-level integration of sensors, batteries, and electronics can be achieved within a miniaturized ring form factor to capture continuous biochemical information from the skin. Optimization of the ring for commercial relevance will be addressed in future work.

In future versions, the electronics and PCB traces can be protected using a thin elastomer coating, such as PDMS or Ecoflex. Alternatively, the electronics compartment can be locally sealed with epoxy, a common encapsulant for electronics. These approaches would improve resistance to incidental wetting, washing, and splashing while preserving the fluidic sensing function. Additionally, transitioning from a hinge-lock design to a fully enclosed concentric configuration—where all components are arranged along the inner circular shell and sealed within a tight elastomeric cover—can further improve water resistance. We have clarified and discussed these limitations on **page 22** in the revised manuscript.

**5.** *The RSSI shows the Bluetooth operation up to 3 m. This is a limited range where BLE connections can typically be maintained to 10s of meters. There is likely a strong impact of human body shadowing and detuning in the antenna, which need to be investigated further. For example, the line-of-sight vs non-line-of-sight scenarios, in particular, depending on the position of the finger relative to the hand (e.g. in a closed grip), or the data being transmitted through the body. The transmitted power level should also be included.*
*Ideally, the antenna properties related to this query should be characterised in more depth, though the RSSI level functional tests could suffice, once performed in the LoS/NLoS arrangements to show the performance limit. Chip antennas are highly dependent on device size, which the ring likely does not meet.*

**Response:** We thank the Reviewer for this insightful comment. We agree that the compact ring form factor, antenna integration, packaging, and body proximity can substantially affect BLE performance. In the original manuscript, the RSSI measurement was performed using the fully packaged ring worn on the body, representing the final use condition, where BLE communication was maintained up to approximately 3 m. To further evaluate the wireless performance limit, we performed additional functional RSSI measurements. Under unobstructed LoS conditions, the bare circuit maintained BLE communication up to approximately 14 m, with RSSI decreasing from approximately −43 dBm at 0 m to −93 dBm at 14 m. When the electronics and chip antenna were fully enclosed within the ring casing but tested off-body, the communication range decreased to approximately 5 m, with RSSI reaching approximately −95 dBm. These results show a progressive reduction in communication range from the bare circuit, to the packaged off-body ring, and finally to the packaged on-body ring, indicating that the 3 m range reflects the practical operating limit of the final wearable configuration rather than the intrinsic range of the BLE radio. As NLoS RSSI measurements strongly depend on the obstruction geometry, material, position, antenna orientation, body posture, and surrounding multipath conditions, we used device-relevant configurations rather than an arbitrary external obstruction. These measurements isolate the effects of packaging and body proximity, which are the dominant practical sources of attenuation, antenna detuning, tissue absorption, and body shadowing in the proposed ring system. The BLE transmission power was set to the wireless module's default level of 0 dBm. We have added these RSSI measurements and transmission power information to the revised manuscript. These related discussions have been mentioned on **page 12** in the revised manuscript version.

**6.** *Please clarify the data transmission details, for example, through firmware flow chart, explaining how the data is encoded, the time needed to transfer the packers, and also if any data processing takes place on the MCU itself, or if it is entirely done on the host PC or smartphone. Both an app and a PC are discussed in the manuscript or video; it will be good to discuss any challenges of using an app.*

**Response:** We thank the Reviewer for this valuable comment. To clarify the wireless data transmission scheme, we have included a new firmware flowchart in the revised manuscript that specifically details the BLE communication protocol (**Fig. S21**). In this process, the acquired data are first packetized on the MCU, and each wake-up event generates a batch of packets that is then sequentially transmitted to the host device via BLE. The total time required for BLE reconnection and packet transfer is approximately 2–10 s, with variation mainly due to the device discovery and connection-establishment stages. Such variability is inherent to BLE communication and can be further affected by the complex electromagnetic environment in the laboratory, including nearby Wi-Fi and Bluetooth activity. No digital signal processing is performed on the MCU; the MCU is used

only for acquisition, temporary buffering, and wireless transmission, whereas all data decoding, visualization, and further processing are carried out on the host PC. This design reduces the MCU's active time, thereby improving power efficiency. A related sentence has been added to **page 10** in the revise manuscript.

**Minor corrections:**

**7.** *Both the MCU and the AFE could operate around 3.6-3.8; it would be good to see the motivation for the buck converter and if it could potentially be removed.*

**Response:** We thank the Reviewer for this insightful comment. Although both the MCU and AFE can operate directly from the battery voltage range, direct battery operation would expose the system to a continuously varying supply voltage during discharge, which may affect the stability of low-current chronoamperometric measurements. In addition, operating the digital circuitry at a lower regulated voltage can reduce power consumption. To evaluate this effect, we performed chronoamperometric measurements using a controlled resistive load. A 500 mV bias was applied across an axial resistor with a measured resistance of 10.24 MΩ, corresponding to an expected current of approximately 0.0488 μA. Within the nominal operating range, the measured mean current remained similar at 2.8, 3.3, and 3.8 V. However, the standard deviation was lowest at 3.3 V, reaching 0.0036 μA compared with 0.0041 μA at 2.8 V and 0.0057 μA at 3.8 V. At 2.3 V, which is outside the recommended operating range, the mean current decreased to 0.0477 μA, and the standard deviation increased to 0.0247 μA, indicating degraded measurement reliability. These results support retaining the buck converter to provide a stable 3.3 V rail, improve measurement stability, avoid undervoltage operation, and reduce digital power consumption relative to operation at the higher battery voltage. We have clarified this motivation and added the supply-voltage characterization results to the revised manuscript.

These new results and related discussion have been added to **page 9** and **Table S2** in the revised manuscript and supplemental version, respectively.

**8.** *The ceramic filter mentioned does not perform impedance matching, as the antenna should be directly matched.*

**Response:** We thank the reviewer for this important clarification. We agree that antenna impedance matching should be achieved through a dedicated matching network, and that the ceramic filter itself is not intended to perform that function. In our design, the ceramic filter is used for harmonic suppression and out-of-band noise filtering. We have revised the manuscript to fix the confusion. This has been clarified on **page 11** in the revised manuscript.

**9.** *For the IR pictures, please include an optical photograph to map the position of the device to the hand. The baseline temperature appears to be low for an on-skin circuit*.

**Response:** We thank the reviewer for this useful suggestion. We have added an optical photograph alongside the IR images to clearly indicate the device's position on the hand (**Fig. 3h** inset). We also note that the measurements were performed using a 3D-printed casing with the same dimensions as the ring, thereby reducing direct thermal coupling to the skin and resulting in a lower apparent baseline temperature. This information is now presented on **page 10** in the revised manuscript version.

10. *Typo: "serail-wire"*

**Response:** We thank the Reviewer for this comment. This typo has been corrected.

## Reviewer #3:

*The manuscript presents an integrated wearable ring platform (CHARM) for multiplexed electrochemical enzymatic sensors in passive sweat captured and transported using hydrogel and paper microfluidic assembly driven by osmotic sweat extraction integrated with wireless electronics, powered by a flexible AgO-Zn battery. While the ring form factor represents an impressive engineering integration and is attractive from a translational standpoint given the growing adoption of smart rings for health monitoring, several aspects of the study raise significant concerns. In particular, the limited size of the human cohort, insufficient calibration and characterization of the microfluidic transport system, and incomplete evaluation of long-term sensor stability weaken the strength of the conclusions.*

*Additionally, the core sensing strategy and the hydrogel-mediated osmotic sweat extraction mechanism have been previously established by the authors in earlier work (see Comment 3). As such, the present manuscript primarily represents an integration of these previously reported elements into a ring-based platform with multiplexing, onboard electronics, and flexible power delivery. Addressing these concerns would significantly strengthen the manuscript and improve its suitability for publication.*

**General Response:** We thank the Reviewer for considering our ring device to be an impressive engineering integration and to be translative in the field of smart rings. Please find the responses for your comments below.

**Specific Comments:**

**1.** *The human validation cohort is extremely limited relative to the breadth of the manuscript's claims. The text refers broadly to testing in "one healthy and one type 1 diabetic individual," and the figures and supplementary information appear to confirm*

*that the in vivo data are derived from repeated measurements from only two identifiable subjects: a single healthy participant and a single T1D participant. Repeated measurements from the same individuals cannot substitute for subject-level biological replication, particularly when the manuscript aims to demonstrate continuous monitoring of multiple chemical biomarkers in real time for at-risk populations.*

**Response:** We thank the Reviewer for raising this valuable point. While we agree that the device was tested on only one healthy and one diabetic participant, we would like to respectfully point out that the biomarker prediction analysis is based on 50–60 days of analysis. We would also like to respectfully point out that repetitive testing on additional healthy individuals would not yield new biomedical insights, but would primarily serve to further validate the device, which has already been extensively demonstrated in **Figs. 5 and 6**. Nevertheless, we are including additional glucose data from testing on a new diabetic subject to **Fig. S40**, with corresponding data points added to **Fig. 6b i**.

2. *In vitro experiments show 6 analytes whereas the on-body tests only mention 4, excluding ascorbic acid (AA) and uric acid (UA). Please elaborate.*

**Response:** We thank the Reviewer for raising this concern. We would like to respectfully clarify that the biomarker combinations chosen in this study focused on two health domains: diabetes and nutrition. For diabetes, biomarkers that are proven to dynamically affect insulin resistance on a daily basis include glucose, ketone, lactate and alcohol, while glucose, ketone, uric acid, and ascorbic acid also hold nutritional significance. Hence, we have chosen a combination of four for testing each condition. Moreover, since all sensors share a single reference electrode, we anticipate that sensitivity will also be compromised on the fifth and sixth electrode due to high IR drop. A sentence highlighting this has been added to **page 4** in the revised manuscript.

3. *As a result, the applicability of the platform to diabetes management and diabetic ketoacidosis risk assessment is insufficiently supported by the presented data. The current dataset does not evaluate device performance across a meaningful glycemic range, across multiple diabetic phenotypes, or under clinically relevant physiological conditions such as hypoglycemia, rapid glycemic excursions, or dehydration. Additional validation across a larger and more diverse cohort would be necessary to support the manuscript's translational claims.*

**Response:** We thank the Reviewer for the follow up question. While we appreciate the suggestion for testing on versatile diabetic cohorts, we would like to respectfully mention that this was not conducted in our current study due to restrictions of our IRB protocol. However, we do intend to do such tests in the future, and so a related sentence has already been added on **page 22** in the revised version.

**4.** *The core sensing concept and hydrogel-mediated extraction strategy were already established in the authors' earlier work. Please see below:*
*a. Saha, Tamoghna, et al. "A passive perspiration inspired wearable platform for continuous glucose monitoring." Advanced Science 11.41 (2024): 2405518.*
*b. Yin, Lu, et al. "High performance printed AgO-Zn rechargeable battery for flexible electronics." Joule 5.1 (2021): 228-248.*

**Response:** We thank the Reviewer for pointing this out. While we agree that the core concepts were previously individually published, this work integrates them for the first time into a synergistic platform for collective wearable biosensing. This makes our work novel in the following ways:

a. The Adv. Sc. paper focuses on fingertip glucose sensing through osmosis. However, in this work, we deploy the concept at the ring finger location for the first time. This allows us to demonstrate the feasibility of passive sweat extraction and sensing at this site and reveals that sweat gland density at the ring location is lower than at the fingertip. We believe such physiological insights are important for the wearable sweat sensing community, which would not be accessible through iontophoresis, since it relies on chemically induced sweating. We have already mentioned these aspects on **page 8** in the original submission. Moreover, given that the ring location is more practical than the fingertip, biochemical sensing at this site also provides meaningful information, thereby extending the monitoring of biomarkers beyond the forearm, where such measurements have traditionally been performed.

b. i) The Joule paper focuses on the fabrication and electrochemical performance of the AgO-Zn battery. However, this battery chemistry has not previously been deployed in a wearable form factor to power multiple chronoamperometric electrochemical sensors. This functionality is enabled by our FPCB's efficient power management and is already mentioned on **page 4** in the original version.

ii) The battery demonstrated by Lu et al. used lead oxide coating on the AgO particles. Since lead oxide is toxic to human body, we eliminated it in our battery system, making it completely free of toxins.

iii) The anode of the battery demonstrated by Lu et al. used a powder composed of Zn powder and $Bi_2O_3$ in 9:1 weight ratio. We further optimized the formula by introducing ZnO into the composition of the anode. This change suppresses the $H_2$ evolution reaction at the anode and thus increases the stability of the anode.

A related sentence highlighting these aspects have been added on **page 27** in the revised version.

**5.** *The manuscript supports the ring site as a practical and wearable location with sufficient sweat access, but it does not establish that this site is biologically superior for biochemical sensing. Although the manuscript argues that the lower sweat gland density at the ring site leads to a reduced sweat flux that may prolong sampling by avoiding rapid channel saturation, it does not show that the ring site is inherently a better location for biochemical sensing. At present, the ring site appears to be a practical compromise rather than a biologically superior sensing site, and the authors should articulate this more clearly.*

**Response:** We thank the Reviewer for this valuable comment. We would like to respectfully clarify that our goal here was not to establish the ring location as superior but rather demonstrate its feasibility for conducting passive sweat measurements at such location. Based on our findings, we are certain that human fingertip remains a superior location for sweat access due to its high sweat gland density possession, which was already mentioned on **pages 8, 15, 21** in the original submission. However, as the fingertip is not a practical site for daily sensing, we believe the ring site's location as a more compliant attractive alternative. A statement highlighting this has been added on **page 8** in the revised manuscript.

**6.** *It is unclear what the maximum sweat storage capacity of the hydrogel component is. Similarly, the manuscript does not specify the total volume capacity of the combined hydrogel paper microfluidic assembly used for sweat collection and transport. In particular, it remains unclear how the system prevents previously absorbed ("old") sweat from mixing with newly generated sweat, which could potentially skew the real-time biochemical readouts.*

**Response:** We thank the Reviewer for this important question. We would like to respectfully note that the hydrogel–paper microfluidic assembly is designed to limit hydrogel swelling while still promoting osmotic sweat collection. This minimizes excessive biomarker collection in the hydrogel, allowing a greater fraction to flow through the channel and be detected by the sensor. This has also been proven this in some of our previous publications: https://pubs.acs.org/doi/abs/10.1021/acsami.0c22730, https://pubs.acs.org/doi/abs/10.1021/acssensors.2c00830, and https://advanced.onlinelibrary.wiley.com/doi/full/10.1002/advs.202405518. A statement clarifying this has been added to **page 4** in the revised manuscript.

Moreover, the calculated weight swelling ratio ($q_p$) ranges ~2.6 for the PVA-PAAm EG soaked gels, indicating that the hydrogels can ideally swell to approximately 260% of their original weight (**page 8** supplemental information). This level of swelling is sufficient for effective sweat collection from the skin. The total volumetric capacity of the paper channel is mentioned on **page 7** in the revised supplemental version. From **Fig. 2b** and **S4**, since

flow saturates after ~8-9 hours, total volume sampled in the channel will range~ 65-70 μL's.

The capillary force of the paper channel prevents the intermixing of old sweat with new. This has been clarified on **page 8** in the revised manuscript version.

7. *Was the flow capacity of the hydrogel evaluated under varying environmental conditions such as relative humidity (RH) and temperature? It is also unclear whether swelling or drying behavior during conditions of elevated sweat production such as physical exertion, hot showers, or sauna-like environments was considered or simulated prior to the in vivo experiments. Clarifying whether these factors were assessed would help establish the robustness of the ring in practical use.*

**Response:** We thank the Reviewer for this valuable question. We believe that RH and temperature will not affect the flow due to the following two reasons: a) Osmosis is a colligative property, which makes it only be dependent on the solute-solvent chemical coefficient difference. Thus, the solute-solvent chemical potential difference between the hydrogel and skin will only dictate the net accumulated sweat. A sentence clarifying this has been added to **page 8** in the revised manuscript version. b) The hydrogel stays well sealed within the circular chamber, preventing exposure to external humidity and temperature variations.

The osmotic effect is minimized with exercise due to flow of active sweat. This has been proven in our previous publications:
https://pubs.acs.org/doi/abs/10.1021/acsami.0c22730,
https://pubs.acs.org/doi/abs/10.1021/acssensors.2c00830
https://advanced.onlinelibrary.wiley.com/doi/full/10.1002/advs.202405518.

Accordingly, in all on-body studies involving lactate testing, we introduce the next biomarker only after at least 2.5–4 hours following the lactate peak. This minimizes the influence of exercise-induced sweat and allows osmotic collection to resume. A sentence clarifying this point has been added to **page 17,19** in the revised manuscript version.

8. *The manuscript uses 150 nL/min as both an experimentally inferred ring-site flow regime and a design/characterization condition, but does not sufficiently justify how representative this is across users and environments? Sweat rates vary substantially among individuals and across environmental conditions, and additional validation across a wider range of sweat production rates, including very low perspiration and higher flow regimes, would strengthen confidence in the sensing platform's robustness.*

**Response:** We thank the Reviewer for this valuable question. While we agree with the Reviewer about the inter-subject sweat rate effects, we have tried our best to addresses these issues in our work. Please check out our response below:

a. Osmosis is a colligative property; therefore, we believe that the sweat extraction rate to remain independent of environmental conditions. Moreover, the hydrogel is fully sealed within its slot inside the ring, limiting exposure to the external environment.
b. We would like to respectfully point out that in **Fig. 2b** of the original version, we have already shown inter-subject sweat rate variations from four subjects. The values range from ~80–170 nL/min, and we attribute these variations to inter-subject differences rather than environmental factors, as osmosis is a colligative property.
c. We have ensured that the sensor response remains independent of exercise-induced sweat and is primarily influenced by only osmotically derived sweat, in all our on-body trials (**Fig. 5 b,c, 6 a iii**).
d. Calibration curves, like in **Fig. 2f,** can be generated for multiple concentrations at the ring flow rate and incorporated as a correction factor in the sensor response to account for unexpected flow rate changes.

Overall, we believe that these measures will certainly enable intersubject variations in sweat rate under different stimuli to be effectively accounted for in the sensor outputs.

**9.** *While the sensing region is positioned ~1.3 cm from the hydrogel inlet and the estimated analyte lag time is only ~2–3 minutes, the manuscript also claims ~10 hours of operation. In this context, it is unclear how the authors address potential drying of gel/evaporation of sweat during the extended useon-body, in changing ambient conditions, and intermittent real-world wear?*

**Response**: We thank the Reviewer for this important question. We would like to respectfully point out that the hydrogel is unlikely to dry out, as it is well sealed within its slot in the 3D-printed structure. Moreover, because the hydrogel is treated with pure ethylene glycol (a non-volatile solvent), dehydration is unlikely, as replacing the full glycol with sweat would require a significant amount of time. Additionally, the collected sweat is not expected to evaporate, as the channel is fully enclosed between two SEBS layers. Related mentions have been added to **page 4** in the revised manuscript and supplementary information, respectively.

**10.** *The manuscript describes the use of personalized calibration parameters and suggests that recalibration may be required if deviations occur. However, it is unclear how frequently users would detect such calibration drift in practice. While the authors report enzymatic sensor stability for up to 9 days, the manuscript claims that the calibration based on the current change (ΔI) remains stable for up to 60 days. It is not clear whether the sensors are stable for 60 days and the manuscript does not sufficiently address how batch-to-batch sensor variability is managed when calibrating the CHARM platform?*

**Response**: We thank the Reviewer for raising this extremely important point. We agree that in our work we have shown sensor stability and calibration as two independent factors. **Fig. S6-S11** shows in-vitro stability of the sensors for a single concentration spike, while **Fig. S36,37,40-42,46-50** shows the stability of the calibration model tested on the same subject under similar conditions with different sensors. The former validates sensor performance, while the latter demonstrates the effectiveness of our sweat sampling technique and sensor combination for accurately capturing the health signatures. Multiple discrete measurements over a 60-day period help establishing the long-term stability of the personalized calibration parameter values with greater confidence.

We understand that the sensor patch would need to be replaced within 9–10 days, if a single sensor is used for continuous sensing. The goal of our study was to demonstrate multi-biomarker monitoring using a ring platform and to establish a method for predicting blood biomarker concentrations from sweat-derived sensor current values. Specifically, we aimed to evaluate how accurately the ring measurements correlate with blood readings during continuous wear over a 10–12-hour period per day. This work therefore focuses on validating the short-term accuracy of the ring system, while long-term performance (continuously over multiple days) and stability will be addressed in future studies. We have added relevant sentences on **page 15** in the revised manuscript version to clarify this.

We would also like to respectfully mention that in-vitro RSD% of every sensors are already shown in **Fig. S6-S11**, demonstrating the inter-batch variability.

**11.** *The cytotoxicity assessment is not well aligned with the intended exposure site of the ring. The biocompatibility is evaluated using THP-1 cells, which are more suitable for a broad immune/cytotoxicity screen than for skin-contact validation. For a wearable intended for prolonged skin interfacing, the authors should use skin-relevant models.*

**Response**: We thank the Reviewer for this important comment. We agree that THP-1 cells are not skin-specific and therefore do not fully recapitulate the biology of prolonged skin contact. In this study, the THP-1 assay was intended as a preliminary general cytotoxicity/immune-compatibility screen rather than a definitive validation of skin safety. A statement highlighting this has also been added to **page 52** on the revised supplemental version. We would also like to respectfully mention that similar assays have also been used in our previous studies: https://www.nature.com/articles/s41928-024-01236-7.

**12.** *Also, it is recommended that the authors include residual PAAm monomer and EG leaching assessment, dermatology testing studies, and multi-day on-skin tolerance data. The current THP-1 assay is insufficient to support the statement that the components are "fully safe to be used on skin."*

**Response**: We thank the Reviewer for this question. We would like to respectfully point out that, in our device design, only the circular portion of the paper channel, the PCB, and the connector pad are in contact with the skin, while all other components are fully sealed. We have clarified this on **page 5** of the revised manuscript.

To support the dermatological tolerance studies, we have also added pictures of skin age, moisture, oil content, and elasticity measurements obtained from two subjects using a commercial device during the continuous wear of ring for 5 days. This data is presented in **Fig. S53** in the revised supplemental version. Readings in the green zone highlight that PAAm monomer and EG have insignificant effect on the skin properties from extended wear.

**13.** *The manuscript does not clearly discuss battery depletion, disposal, or replacement strategies. It would be helpful to clarify whether the device is intended to be disposable, rechargeable, or modular, and whether components such as the battery or sensing elements can be replaced.*

**Response:** We thank the Reviewer for this valuable question. Overall, CHARM is currently a modular system, where the sensor-fluidic assembly is disposable, the battery is rechargeable, and the electronics in reusable. However, in future work, we aim to design the device such that the sensor–fluidic components can be easily replaced, and the battery can be conveniently recharged by the user.

**14.** *The manuscript does not clearly address the storage shelf life of the enzyme-based sensing module. It is unclear whether the reported 60-day stability refers to the operational stability of the complete device (hydrogel, microfluidics, enzymatic sensors, and electronics) or only to the storage stability of individual components. In either case, the authors should specify the storage conditions required to maintain hydrogel functionality, enzyme activity, and battery performance, including potential self-discharge. The authors should also clarify whether the reported long-term stability reflects pre-use shelf life, on-body operational stability, or short-term benchtop sensor performance.*

**Response**: We thank the Reviewer for this question. The current storage shelf life of the sensors is 9-10 days, as shown in **Fig. S6-S11**. The hydrogel storage shelf life is ~14 days. The long-term stability (over 60 days) reflects the consistency of the personalized calibration parameters (a, b), measured on the same individual under similar stimuli using a new sensor for each measurement. A related sentence has been added to **page 23** in the revised manuscript. The self-discharging performance of the battery is shown in **Fig. S33,** and its related text is presented on **page 14** in the revised version. Overall, we observe ~2.74 mAh drop after running 45 continuous cycles for 8 days.

**15.** The nomenclature for the analytes should be maintained consistently throughout the manuscript. (for example, Ascorbic Acid and Vitamin C).

**Response**: We thank the Reviewer for mentioning this. It has been corrected in the revised manuscript.

## Responses to Reviewer Comments

**Reviewer #1:** *The reviewer appreciates the authors' effort to address the comments, but the previous comment on calibration remains inadequately addressed or explained.*

*With the introduction of both slope (a) and intercept (b), the sensor readings no longer directly reflect absolute sweat chemical concentrations nor the magnitude of dynamic changes. Without multiple calibrations against a standardized device (e.g., a blood glucose meter), it is unclear how users can obtain accurate readings.*

*Across Figures S36–S50, the values of a and b vary considerably for all sensors. Even within a single subject, these calibration parameters show significant variation — for example, the pronounced variation in b-value in Figure S50. Without recalibration against a standard device each time to obtain updated a and b values, the resulting concentration errors would likely be substantial. The authors attribute this variation to individual differences but provide no data excluding sensor variability. Does sensor variability have no influence on the final readings? If users perform multiple calibrations, regardless of a and b variation, can accurate readings still be guaranteed? How would users determine which a or b value to use in daily practice?*

*The reviewer requests that the authors add a clear calibration methodology and explanation to the manuscript to guide readers in fully understanding the procedure. Please highlight the changes; otherwise, it is extremely difficult to locate them when none are marked, especially when mixed with different bolded texts.*

**Response:** We thank the Reviewer for this important question regarding our calibration model. Our detailed response regarding its rationale and execution strategy is provided below:

a. We would like to respectfully clarify that our calibration equation is based on an empirical approach, where coefficients *a* and *b* are determined using the sensor-measured current and the corresponding biomarker concentration in blood (page 35, SI). These coefficients are then used to convert the current values into concentration values using the $SBG(t)$ equation. Thus, all blood biomarker concentrations are "*empirically predicted*" from the sensor current response. We strongly believe that this approach is feasible for obtaining a non-invasive estimate of blood biomarker concentration, particularly because i) the flux between the sensor surface and the channel changes continuously due to continuous sweat flow, hence the in-vitro calibration slope cannot be directly used to convert the current to concentration; ii) The model derives the coefficients from the overall maximum signal change observed under a given condition for each individual.

Moreover, *a* primarily governs the overall trend of $SBG(t)$, as blood concentration is involved in its denominator. In contrast, *b* incorporates the baseline current, which is generally low prior to any stimulus. To further simplify and make it easier, we have expanded the $SBG(t)$ equation into a $y = ax + b$ form (on **page 35** in SI), to help readers understand how $a$ and $b$ are being used to derive $SBG\ (t)$.

b. In this work, we are deriving $a$ and $b$ values based on the maximum change in blood concentration. This logic specifically applies to glucose, as we spike it in subjects with multiple stimuluses, but the maximum change was only observed through meal. For other analytes, a single stimulus was used to elevate the levels.

c. We would like to point out that the values of $a$ and $b$ are expected to vary between each biomarker. This is because both $a$ and $b$ are derived from the sensor current response, which is dependent on the biomarker concentration, sensor composition, and on the reaction kinetics of the enzymatic reaction.

d. We agree that these empirically derived coefficients may vary across repeated measurements on different days. This can happen from normal biological fluctuations, physiological state, and environmental factors effects between the periods of measurements. Consequently, the derived concentrations can deviate from the true concentrations measured using gold-standard techniques. Hence, we check the stability of both $a$ and $b$ over multiple days under the same conditions for each individual. **Fig. S35** shows the calibration strategy that every individual should follow in daily practice. Basically, if measurements of both $a$ and $b$ over consecutive days deviate by >10%, the subject can recalibrate the values by measuring again the respective biomarker concentration in blood using commercial meters. This approach will help improve the prediction accuracy.

e. Sensor variability is not expected to have a substantial impact on the derived *a* and $b$ values because these parameters are determined separately for each measurement day using a freshly prepared sensor. Thus, the sensor is in its maximum operating efficiency. Hence, if any deviations occur in the value of $a$ and $b$, it is most likely to arise from individual-specific effects, rather than sensor-related variability. Our proposed approach aims to evaluate the long-term stability of these parameters (demonstrated here till ~ 2months), before finalizing them to enable blood-calibration free estimation of respective blood biomarker concentrations from sweat currents. However, we agree that increasing the measurement frequency could provide a closer tracking of short-term fluctuations in these values. In contrast, we believe that long-term measurements are better suited to capture overall trends and average out the variations arising from the potential factors

listed in (c). For the accuracy of system, we would like to respectfully point out the low standard deviation of $a$ and $b$ values across all testing regimes in **Fig. S36,37,40-50**).

We have included these detailed explanations on **pages 15-16,22** (highlighted) to provide readers with a clearer understanding of how the calibration process works. Overall, these coefficients can be stored and updated in real time through a smartphone app linked to CHARM; here, we demonstrate this strategy using representative cases.

Please note that value of $-b$ in **Fig. S50** Day 1 was previously a typo and has now been corrected to $0.018\ \frac{\mu A}{cm^2}$. This is evident from the similarity between sensor current and BAC% trends across Days 1, 22, and 48, which was already previously reported.

**Reviewer #2:** *Thank you for addressing the comments in detail and providing the requested additional experimental data. I believe the work is now suitable for publication. Please also make the following minor correction, related to the BLE performance:*

*The measured communication range and RSSI show that the antenna gain is extremely low (below -25 dBi without obstruction). This is likely due to the limitations of a chip antenna on a very constrained layout, while typically requiring large devices.*

*In Line 294, the manuscript reads:*
*"The RF path includes an integrated ceramic low-pass filter for harmonic suppression and out-of-band noise filtering, complemented by a compact ceramic antenna for efficient transmission"*
*I recommend that the phrase "efficient transmission" is removed, as the antenna/RF front end has higher losses than typical (which understandably does not hinder the application).*

**Response:** We thank the Reviewer for this useful suggestion. This has now been addressed in **page 11** of the revised manuscript version.

**Reviewer #3:** *The authors have addressed all my concerns, and the manuscript can be accepted in the present form.*

**Response:** We thank the Reviewer for acknowledging us having addressed all previously raised questions and for considering the manuscript suitable for publication in its current form.
